# Precision hematology: Navigating the evolution of diagnostic classifications in the era of globalized medicine

**DOI:** 10.1002/hem3.65

**Published:** 2024-04-04

**Authors:** Elizabeth Macintyre, Konstanze Döhner, Kirsten Grønbæk, Martin Dreyling, Brian Huntly, Antonio Almeida, John Gribben, for the EHA Board

**Affiliations:** ^1^ Laboratory of Onco‐Hematology, Necker‐Enfants Malades Hospital Assistance Publique‐Hôpitaux de Paris (AP‐HP) Paris France; ^2^ Université Paris Cité, CNRS, INSERM U1151 Institut Necker Enfants Malades (INEM) Paris France; ^3^ Department of Internal Medicine III University Hospital of Ulm Ulm Germany; ^4^ Department of Hematology, Rigshospitalet Copenhagen University Hospital Copenhagen Denmark; ^5^ Department of Clinical Medicine, Faculty of Health and Medical Sciences University of Copenhagen Copenhagen Denmark; ^6^ Biotech Research and Innovation Center (BRIC) University of Copenhagen Copenhagen Denmark; ^7^ Medizinische Klinik III, Klinikum der Universität LMU München Germany; ^8^ Department of Haematology, University of Cambridge Cambridge Stem Cell Institute Cambridge UK; ^9^ Department of Hematology Hospital da Luz Lisboa Portugal; ^10^ Faculdade de Medicina Universidade Catolica Portuguesa Lisboa Portugal; ^11^ Center for Haemato‐Oncology, Barts Cancer Institute Queen Mary University of London London UK

In the era of precision medicine, hematology stands at the forefront of medical disciplines. Indeed, our enduring commitment to employing molecular techniques for disease characterization, coupled with their application in individual patient management at diagnosis and during treatment monitoring, places hematology in a pivotal position. We continue to seamlessly integrate an ever‐expanding array of diagnostic techniques, developed and crafted by a variety of medical and scientific specialists. These experts have collaborated in a multidisciplinary fashion to define and communicate the most appropriate diagnosis and treatment for patients with hematological cancers and nonmalignant disorders. However, this increasingly demands progressively specialized training tracks and skill sets.

The archetypical hematologist, who historically personally saw the patient, biopsied the bone marrow or even spleen, stained the cells, and then made a morphological/cytological diagnosis before starting and accompanying treatment, has become a relic of the past. At a minimum, achieving precision diagnosis of tumors of hematopoietic and lymphoid tissues requires hematologists (diagnostic and therapeutic), pathologists, geneticists, biomedical scientists/technicians/engineers, and radiologists/nuclear medicine specialists.

In any community, rules and regulations are imperative for peaceful, constructive coexistence. These regulations must be clear, concise, and up‐to‐date and then be communicated to and understood by all actors involved in their execution. This is a formidable challenge in a globalized world with exponentially evolving technological and informatic/data capabilities. Classifications must be provided by experts, who should be free of conflict of interest and capable of providing guidelines for the treatment of all patients across a gradient of increasingly disparate access to health.

The World Health Organization (WHO) tumor classification system, available at https://www.iarc.who.int, has long served as the accepted standard reference. This classification has undergone multiple updates over the years, reflecting our evolving knowledge of tumor biology and advancements in diagnostic and therapeutic approaches. At a more specialized level, national and international medical specialist societies have become important actors, initially in organizing scientific meetings and supporting research, and subsequently expanding into education, training, mentorship, advocacy, and support for postgraduate practice, including guideline production/endorsement. Indeed, in the latest survey of European Hematology Association (EHA) members, with replies from 4966 individuals in six continents, on the EHA services they appreciate, guidelines came second on the list, just after high‐quality education. Collaboration between WHO/IARC (International Agency for Research on Cancers) and specialist societies, therefore, remains crucial.

In 2008, the collaborative effort of the European Association for Haematopathology (EAHP), the Society for Hematopathology (SH), and the WHO/IARC led to the release of the fourth edition of the WHO Classification of Tumors of Hematopoietic and Lymphoid Tissues (WHO‐HAEM4). Oversight was provided by an eight‐person steering committee composed of members from all three. A clinical advisory committee (CAC) comprising hematologists, pathologists, oncologists, and geneticists was convened to provide insights and reach a consensus on the proposed contents. In 2017, a major update to the 4th edition (WHO‐HAEM4R) involved many of the original editors as well as additional senior advisors with expertise in myeloid neoplasms and molecular/cytogenetic issues. As in previous editions, a CAC was convened to contribute to the WHO‐HAEM4R and the final volume had more than 200 contributors from 24 countries.

Significant developments since 2017 necessitated a further update. Following new governance rules by the WHO/IARC to develop the WHO 5th edition, an editorial board of *standing* members as well as *expert* members was appointed by the IARC. This board established the draft classification (table of contents) and assigned multidisciplinary authorship groups including hematologists, oncologists, pathologists, radio‐oncologists, and geneticists. The resulting classification was presented in two companion manuscripts in 2022 covering the classification of myeloid and histiocytic/dendritic neoplasms^1^ and the classification of lymphoid neoplasms,^2^ as well as a beta v2 version released online at https://shop.iarc.fr/products/annual-subscription?variant=29448046608432.

However, dissatisfaction with the process used to develop the 5th edition was expressed by the executive committees of the EAHP and SH. Concerns were raised about the lack of oversight from the SH and EAHP and a failure to follow a formal CAC process. To address this, a CAC endorsed by SH and EAHP, comprising an international group of pathologists, clinicians, and scientists separate from the WHO, was convened to update the WHO 2017 classification. This resulted in special reports in *Blood* in 2022 and 2023 describing the International Consensus Classification (ICC) of myeloid neoplasms, acute leukemias, and mature lymphoid neoplasms,^3^, ^4^, ^5^ and a monographic issue of *Virchows Archives* in January 2023.^6^

Under the guidance of the EHA board and with the input of experts in the field, two documents were prepared to shed light on the differences between the new classifications. One document describes the updates to the classification of mature lymphoid neoplasms (File S1), while the second document describes the updates to the classification of myeloid neoplasms and acute leukemias (File S2).

These documents highlight to what extent the two new classifications developed in 2022 have changed compared with the WHO‐HAEM4R (referred to in this paper and supplements as the WHO 2017 blue book). The changes range from minor to significant. Examples of myeloid malignancies include the definition of accelerated phase CML criteria in ICC but not in WHO 2022. In MDS, both classifications introduce *TP53* mutation as a separate entity, albeit with minor differences in precise molecular/cytogenetic criteria. The ICC introduced an MDS/AML category in the presence of 10%–19% blasts, whereas these are referred to as MDS‐Increased Blasts 2 (IB2) in WHO 2022. *SF3B1* mutation is now included in both classifications as a diagnostic criterion for what was previously “refractory anemia with ring sideroblasts,” along with the omission of the number of dysplastic lineages in the classification of low blast count MDS. Minor changes include the addition of more precise genetic subgroups to several malignancies, such as eosinophilia, and a reduction in the level of the absolute monocyte count to 0.5 × 10^9^/L for the diagnosis of CMML in both classifications, but with maintained criteria for associated cytopenias only in ICC 2022. For the lymphoid malignancies, examples of differences include large B‐cell lymphoma with *IRF4* rearrangement, which is classified with follicular lymphoma by ICC and with large cell lymphoma in WHO 2022, or B‐cell prolymphocytic leukemia (PLL) which is not listed as a separate entity in WHO 2022 but grouped with splenic B‐cell lymphoma. There are also differences in the classification of splenic B‐cell lymphoma, non‐IgM Lymphoplasmacytic Lymphoma (LPL), and MGUS subgroups.

Divergences in academic medical communities generating innovative understanding of hematopoietic and lymphoid cancers and their management are normal and healthy. On Darwinian principles, evolution requires cross‐fertilization, and no classification can be written in stone. However, divergent classifications pose challenges for those who practice hematology in varied settings. It is incumbent upon our multidisciplinary community to capitalize on these divergences in order to optimize and standardize the diagnosis and monitoring of hematopoietic and lymphoid cancers. Indeed, this is already happening. Constructive discussion in the public domain through controlled feedback is welcome, whether this occurs through classical communication via medical journals,^7^, ^8^ appropriate online channels, or conferences. Such discussions aim to transform apparent divergence into enlightened understanding, addressing issues such as the place of chromosome banding patterns or morphological karyotyping in the era of molecular diagnostics.^9^, ^10^

Priority should be given to biomarkers of theranostic or prognostic significance. It is essential to acknowledge that we do not know the optimal biomarkers of the future and also that the best outcome of a biomarker for poor prognosis is its loss of predictive value following improved therapeutic intervention.

The EHA board chose to highlight significant differences between the new classifications compared with the WHO 2017 blue book, aiming to contribute to fruitful reconvergence, in order to facilitate daily practice for hematologists and improve service to our patients with blood disorders. From the standpoint of guidelines/regulatory/standards, classifications must navigate between “perpetual motion” and optimizing the integration of appropriate innovations, a field known as implementation science or the “know‐do” gap. While it is hoped that artificial intelligence and automated learning will aid in this process, it is clear that bringing relevant innovation to patients will require clear communication between regulators, the diagnostic manufacturing sector, academics, clinicians, scientists, and patients.

## AUTHOR CONTRIBUTIONS

All authors were involved in the writing of the manuscript, more specifically the editorial (Elizabeth Macintyre and John Gribben), the myeloid supplement (Konstanze Döhner, Kirsten Grønbæk, Brian Huntly, Antonio Almeida), and the lymphoid supplement (Elizabeth Macintyre, Martin Dreyling, John Gribben). We thank Elias Campos and German Ott for providing constructive comments.

## CONFLICT OF INTEREST STATEMENT

E. Macintyre has received honoraria from Servier. K. Döhner has received research support from Novartis, Celgene/BMS, Astellas, Agios, Abbvie, and JAZZ; has received honoraria from Novartis and GSK; and has served on the Advisory Board for Novartis, Celgene/BMS, Abbvie, GSK, AOP, and MSD. K. Grønbæk has received research support from Janssen Pharma; and has served on the Advisory Board for GSK, Evaxion, and Nanexa. M. Dreyling has received institutional research support from Abbvie, Bayer, BMS/Celgene, Gilead/Kite, Janssen, Lilly, and Roche; has received speaker honoraria from AstraZeneca, Beigene, Gilead/Kite, Janssen, Lilly, Novartis, and Roche; and has served on the Scientific Advisory Board for Abbvie, AstraZeneca, Beigene, BMS/Celgene, Gilead/Kite, Janssen, Lilly/Loxo, Novartis, and Roche. B. Huntly has performed consultancy for Exscientia, Stelexis Therapeutics, and Appollo Therapeutics; has received research funding from Astra‐Zeneca and Sysmex. A. Almeida has served as a speaker for Abbvie, BMS, Janssen, and Novartis; has served on the Advisory Board of Abbvie, BMS, and Novartis. J. Gribben has received research funding from Astra Zeneca and Janssen; honoraria from Amgen, Gilead, and Astra Zeneca; and research funding to the Institute for clinical trials from Janssen, Genmab, and Abbvie.

## FUNDING

This research received no funding.

## Supporting information


**Supplement 1**: Updates to the classification of mature lymphoid neoplasms: an overview of changes since the WHO 2017 Blue Book.

**Supplement 2**: Updates to the classification of myeloid neoplasms and acute leukaemias: an overview of changes since the WHO 2017 Blue Book.
