# Supplementary material for: Precision hematology: Navigating the evolution of diagnostic classifications in the era of globalized medicine
Source: Hemasphere. 2024 Apr 4;8(4):e65. doi: 10.1002/hem3.65 (PMC10993146; doi:10.1002/hem3.65)
Supplement: Supplementary file 1 — Supplement 1: Updates to the classification of mature lymphoid neoplasms: an overview of changes since the WHO 2017 Blue Book. [file HEM3-8-e65-s001.docx]

**Updates to the classification of mature lymphoid neoplasms:**

**an overview of changes since the WHO 2017 Blue Book**

Contents

[1. Introduction 2](#_Toc149060742)

[2. Mature B cell neoplasms 4](#_Toc149060743)

[2.1 Categorisation 4](#_Toc149060744)

[2.2 Pre-neoplastic and neoplastic small lymphocytic proliferations 12](#_Toc149060745)

[2.3 Splenic B cell lymphomas and leukaemias 14](#_Toc149060749)

[2.4 Lymphoplasmacytic lymphoma (LPL) and Waldenström macroglobulinaemia 15](#_Toc149060750)

[2.5 Marginal zone lymphomas (MZLs) 16](#_Toc149060751)

[2.6 Follicular lymphoma (FL) 16](#_Toc149060753)

[2.7 Primary cutaneous follicle centre lymphoma 17](#_Toc149060755)

[2.8 Transformations of indolent B-cell lymphomas 18](#_Toc149060756)

[2.9 Mantle cell lymphoma (MCL) 18](#_Toc149060757)

[2.10 Large B-cell lymphomas 18](#_Toc149060758)

[2.11 Burkitt lymphoma (BL) 21](#_Toc149060767)

[2.12 (KSHV/) HHV-8-associated lymphoproliferative disorders 21](#_Toc149060768)

[2.13 Lymphoid proliferations and lymphomas associated with immune deficiency and dysregulation 21](#_Toc149060769)

[2.14 Monoclonal gammopathies 22](#_Toc149060771)

[2.15 Heavy chain diseases 23](#_Toc149060776)

[2.16 Plasma cell neoplasms 23](#_Toc149060777)

[2.17 Monoclonal immunoglobulin (Ig) deposition diseases 24](#_Toc149060781)

[3. Hodgkin lymphomas 25](#_Toc149060784)

[3.1 Categorisation 25](#_Toc149060785)

[3.2 Nodular lymphocyte predominant Hodgkin lymphoma (NLPHL) 26](#_Toc149060786)

[3.3 Classic Hodgkin lymphoma (CHL) 26](#_Toc149060787)

[4. Mature T- and NK-cell neoplasms 27](#_Toc149060788)

[4.1 Categorisation 27](#_Toc149060789)

[4.2 Mature T-cell and NK-cell leukaemias 31](#_Toc149060790)

[4.3 EBV-positive T-cell and NK-cell lymphoproliferative diseases / lymphoid proliferations and lymphomas of childhood 33](#_Toc149060797)

[4.4 EBV-positive NK/T-cell lymphomas 34](#_Toc149060801)

[4.5 Intestinal T-cell and NK-cell lymphoid proliferations and lymphomas / T and NK-cell lymphomas and lymphoproliferative disorders of the gastrointestinal tract 34](#_Toc149060804)

[4.6 Hepatosplenic T-cell lymphoma 35](#_Toc149060808)

[4.7 Primary cutaneous T-cell lymphoproliferative disorders and lymphomas 35](#_Toc149060809)

[4.8 Peripheral T-cell lymphoma, not otherwise specified (PTCL, NOS) 36](#_Toc149060812)

[4.9 Nodal lymphomas of T follicular helper (TFH) cell origin 36](#_Toc149060813)

[4.10 Anaplastic large cell lymphoma (ALCL) 36](#_Toc149060814)

[5. Immunodeficiency-associated lymphoproliferative disorders 37](#_Toc149060818)

[5.1 Categorisation 37](#_Toc149060819)

[5.2 ICC 2022 classification updates 39](#_Toc149060820)

[5.3 WHO 2022 framework and nomenclature changes 39](#_Toc149060821)

[6. Histiocytic and dendritic cell neoplasms 40](#_Toc149060822)

[6.1 Categorisation 40](#_Toc149060823)

[6.2 Inflammatory pseudotumour-like follicular/fibroblastic dendritic cell sarcoma 42](#_Toc149060824)

[6.3 ALK-positive histiocytosis 43](#_Toc149060825)

[6.4 Rosai–Dorfman(–Destombes) disease 43](#_Toc149060826)

[6.5 Other histiocytic and dendritic cell neoplasms 43](#_Toc149060827)

[7. Conclusion 43](#_Toc149060828)

[8. Acknowledgements 44](#_Toc149060829)

# Introduction

The classification of tumours of haematopoietic and lymphoid tissues is multifaceted and holds a crucial role in the management of patients with haematological malignancies. The World Health Organization (WHO) classification system has long served as an accepted standard reference and has undergone multiple updates over the years to incorporate our evolving knowledge of tumour biology and advancements in diagnostic and therapeutic approaches.

In 2008, the fourth edition of the WHO Classification of Tumours of Hematopoietic and Lymphoid Tissues (WHO-HAEM4) was released as a collaborative effort between the European Association for Haematopathology (EAHP), the Society for Hematopathology (SH) and the WHO International Agency for Research on Cancer (IARC). This 4th edition was overseen by an eight-person steering committee composed of members from the three societies. A clinical advisory committee (CAC) made up of haematologists, pathologists, oncologists and geneticists was convened to provide insights and reach consensus on the proposed contents. In 2017, a major update to the 4th edition (WHO-HAEM4R) involved many of the original editors as well as additional senior advisors with expertise in myeloid neoplasms and molecular/cytogenetic issues. A CAC was convened to contribute to the WHO-HAEM4R as per previous editions. The final volume had more than 200 contributors from 24 countries.

Significant developments since 2017 resulted in a need for a further update. Following the establishment of new governance rules by the WHO/IARC, to develop the WHO 5th edition, an editorial board of *standing* members as well as *expert* members was appointed by the IARC for their leadership and expertise relevant to a particular volume. The editorial board established the draft classification (table of contents) and assigned multidisciplinary authorship groups including haematologists, oncologists, pathologists, radio-oncologists and geneticists. A clinical advisory board meeting was held and the resulting classification was presented in two companion manuscripts released in 2022 covering the classification of myeloid and histiocytic/dendritic neoplasms and the classification of lymphoid neoplasms, as well as a beta v2 version released online.

However, the executive committees of the EAHP and SH expressed dissatisfaction with the process used to develop the 5th edition. There were concerns raised about the lack of oversight from the SH and EAHP and a failure to follow a formal CAC process, as had been done before. To address this, a CAC comprising an international group of pathologists, clinicians and scientists, endorsed by SH and EAHP, separate from the WHO, was convened to update the WHO 2017 classification. This resulted in four special reports in *Blood* in 2022 describing the International Consensus classification (ICC) of myeloid neoplasms, acute leukaemias and mature lymphoid neoplasms, and a monographic issue of *Virchows Archives* in January 2023.

The aim of this document is to highlight to which extent the two new classifications developed in 2022 have changed compared with the WHO-HAEM4R (referred to in this paper as the WHO 2017 blue book). This document will focus on updates to the classification of mature lymphoid neoplasms, and therefore will compare and contrast the 5th edition of the World Health Organization Classification of Haematolymphoid Tumours: Lymphoid Neoplasms review article in *Leukaemia* (WHO 2022)* and the International Consensus Classification of Mature Lymphoid Neoplasms: a report from the Clinical Advisory Committee in *Blood* (ICC 2022)†.

The following five subsections are included below:

- Mature B-cell neoplasms
- Hodgkin lymphomas
- Mature T- and NK-cell neoplasms
- Immunodeficiency-associated lymphoproliferative disorders
- Histiocytic and dendritic cell neoplasms

Tumour-like lesions with B-cell and T-cell predominance and precursor B- and T-cell neoplasms are not covered in this document because they are outside the scope of this review. These entities are covered in the WHO 2022 classification; precursor B-cell neoplasms are covered in the ICC 2022 classification within the myeloid and acute leukaemias paper.

One general update since 2017 implemented in the WHO 2022 classification is that there are no provisional entities as these, by definition, lack sufficient evidence for forming an entity. The ICC 2022 classification maintains the concept of provisional entities for those that have distinct features but not enough evidence to be distinguished from related disorders.

* In some cases, the 5th edition (beta v2) available online was used to provide additional information [last accessed 30 October 2023].

† In some cases, the 2023 monographic issue of *Virchows Archives* was used to provide additional information.

# Mature B-cell neoplasms

Mature B-cell neoplasms are a group of heterogeneous diseases of the B-lymphatic system that share similar disease courses and treatment paradigms. Many patients with a mature B- cell neoplasm will have a good prognosis; however, some disorders are more aggressive than others.

## 2.1 Categorisation

Most of the mature B-cell neoplasm entities from the WHO 2017 blue book are included within the ICC 2022 and WHO 2022 classifications, but new types have been identified and added to the 2022 classification documents. The differences between each document are listed in Table 1.

Table 1. Mature B-cell neoplasm entities

| **ICC 2022** | **WHO 2017** | **WHO 2022** |
| --- | --- | --- |
| **Pre-neoplastic and neoplastic small lymphocytic proliferations** | | |
| Chronic lymphocytic leukaemia/small lymphocytic lymphoma (CLL/SLL) | Chronic lymphocytic leukaemia/small lymphocytic lymphoma (CLL/SLL) | Chronic lymphocytic leukaemia/small lymphocytic lymphoma (CLL/SLL) |
| Monoclonal B-cell lymphocytosis | Monoclonal B-cell lymphocytosis | Monoclonal B-cell lymphocytosis |
| B-cell prolymphocytic leukaemia | B-cell prolymphocytic leukaemia |  |
| **Splenic B-cell lymphomas and leukaemias** | | |
| Splenic marginal zone lymphoma | Splenic marginal zone lymphoma | Splenic marginal zone lymphoma |
| Hairy cell leukaemia | Hairy cell leukaemia | Hairy cell leukaemia |
| *Splenic B-cell lymphoma/leukaemia, unclassifiable* | *Splenic B-cell lymphoma/leukaemia, unclassifiable* |  |
| *Splenic diffuse red pulp small B-cell lymphoma* | *Splenic diffuse red pulp small B-cell lymphoma* | Splenic diffuse red pulp small B-cell lymphoma |
| *Hairy cell leukaemia variant* | *Hairy cell leukaemia variant* | Splenic B-cell lymphoma/leukaemia with prominent nucleoli  (also absorbs cases of CD5-negative B-prolymphocytic leukaemia) |
| **Lymphoplasmacytic lymphoma** | | |
| Lymphoplasmacytic lymphoma | Lymphoplasmacytic lymphoma | Lymphoplasmacytic lymphoma |
| Waldenström macroglobulinemia | Waldenström macroglobulinemia | IgM LPL/Waldenström macroglobulinemia type |
|  |  | Non-IgM LPL/Waldenström macroglobulinemia type |
| **Marginal zone lymphoma** | | |
| Extranodal marginal zone lymphoma of mucosa-associated lymphoid tissue (MALT lymphoma) | Extranodal marginal zone lymphoma of mucosa-associated lymphoid tissue (MALT lymphoma) | Extranodal marginal zone lymphoma of mucosa-associated lymphoid tissue |
| Primary cutaneous marginal zone lymphoproliferative disorder |  | Primary cutaneous marginal zone lymphoma |
| Nodal marginal zone lymphoma | Nodal marginal zone lymphoma | Nodal marginal zone lymphoma |
| *Paediatric nodal marginal zone lymphoma* | *Paediatric nodal marginal zone lymphoma* | Paediatric nodal marginal zone lymphoma |
| **Follicular lymphoma** | | |
| Follicular lymphoma | Follicular lymphoma | Follicular lymphoma |
| In situ follicular neoplasia | In situ follicular neoplasia | In situ follicular B-cell neoplasm |
| Duodenal-type follicular lymphoma | Duodenal-type follicular lymphoma | Duodenal-type follicular lymphoma |
| Testicular follicular lymphoma | Testicular follicular lymphoma |  |
| Paediatric-type follicular lymphoma | Paediatric-type follicular lymphoma | Paediatric-type follicular lymphoma |
| *BCL2-R-negative, CD23-positive follicle centre lymphoma* |  | **P** |
| Large B-cell lymphoma with *IRF4* rearrangement | *Large B-cell lymphoma with IRF4 rearrangement* | (Listed with large B-cell lymphomas in the WHO 2022 classification) |
| **Cutaneous follicle centre lymphoma** | | |
| Primary cutaneous follicle centre lymphoma | Primary cutaneous follicle centre lymphoma | Primary cutaneous follicle centre lymphoma |
|  |  | **Transformations of indolent B-cell lymphomas** |
|  |  | Transformations of indolent  B-cell lymphomas |
| **Mantle cell lymphoma** | | |
| Mantle cell lymphoma | Mantle cell lymphoma | Mantle cell lymphoma |
| In situ mantle cell neoplasia | In situ mantle cell neoplasia | In situ mantle cell neoplasm |
| Leukaemic non-nodal mantle cell lymphoma | Leukaemic non-nodal mantle cell lymphoma | Leukaemic non-nodal mantle cell lymphoma |
| **Large B-cell lymphomas** | | |
| Diffuse large B-cell lymphoma (DLBCL), NOS | Diffuse large B-cell lymphoma (DLBCL), NOS | Diffuse large B-cell lymphoma (DLBCL), NOS |
| Germinal centre B-cell subtype | Germinal centre B-cell subtype | Germinal centre B-cell subtype |
| Activated B-cell subtype | Activated B-cell subtype | Activated B-cell subtype |
| Nodular lymphocyte predominant B-cell lymphoma* | (Included as ‘Nodular lymphocyte predominant Hodgkin lymphoma’ and listed under **Hodgkin lymphomas** chapter – see section 3 below) | (Included as ‘Nodular lymphocyte predominant Hodgkin lymphoma’ and listed under **Hodgkin lymphomas** chapter – see section 3 below) |
| T-cell/histiocyte-rich large  B-cell lymphoma | T-cell/histiocyte-rich large  B-cell lymphoma | T-cell/histiocyte-rich large  B-cell lymphoma |
| Primary diffuse large B-cell lymphoma of the central nervous system | Primary diffuse large B-cell lymphoma of the central nervous system | Primary large B-cell lymphoma of immune-privileged sites |
| Primary diffuse large B-cell lymphoma of the testis |  |  |
| Primary cutaneous diffuse large B-cell lymphoma,  leg type | Primary cutaneous diffuse large B-cell lymphoma,  leg type | Primary cutaneous diffuse large B-cell lymphoma,  leg type |
| (Listed with follicular lymphomas in the ICC 2022 classification) | *Large B-cell lymphoma with IRF4 rearrangement* | Large B-cell lymphoma with *IRF4* rearrangement |
| *HHV-8 and EBV-negative primary effusion-based lymphoma* |  | Fluid overload-associated large B-cell lymphoma |
| EBV-positive diffuse large  B-cell lymphoma, NOS | EBV-positive diffuse large  B-cell lymphoma, NOS | EBV-positive diffuse large  B-cell lymphoma |
| Diffuse large B-cell lymphoma associated with chronic inflammation | Diffuse large B-cell lymphoma associated with chronic inflammation | Diffuse large B-cell lymphoma associated with chronic inflammation |
| Fibrin-associated diffuse large B-cell lymphoma | Fibrin-associated diffuse large B-cell lymphoma | Fibrin-associated large B-cell lymphoma |
| Lymphomatoid granulomatosis | Lymphomatoid granulomatosis | Lymphomatoid granulomatosis |
| EBV-positive polymorphic  B-cell lymphoproliferative disorder, NOS |  |  |
| Primary mediastinal large  B-cell lymphoma | Primary mediastinal (thymic) large B-cell lymphoma | Primary mediastinal large  B-cell lymphoma |
| Intravascular large B-cell lymphoma | Intravascular large B-cell lymphoma | Intravascular large B-cell lymphoma |
| ALK-positive large B-cell lymphoma | ALK-positive large B-cell lymphoma | ALK-positive large B-cell lymphoma |
| Plasmablastic lymphoma | Plasmablastic lymphoma | Plasmablastic lymphoma |
| **High-grade B-cell lymphoma†** | | |
| High-grade B-cell lymphoma with *MYC* and *BCL2* rearrangements | High-grade B-cell lymphoma with *MYC* and *BCL2* and/or *BCL6* rearrangements | Diffuse large B-cell lymphoma/ high-grade B-cell lymphoma with *MYC* and *BCL2* rearrangements |
| *High-grade B-cell lymphoma with MYC and BCL6 rearrangements* |  | (Included as a genetic subtype of DLBCL, NOS or high-grade B-cell lymphoma, NOS) |
| High-grade B-cell lymphoma, NOS | High-grade B-cell lymphoma, NOS | High-grade B-cell lymphoma, NOS |
| *Large B-cell lymphoma with 11q aberration* | *Burkitt-like lymphoma with 11q aberration* | High-grade B-cell lymphoma with 11q aberrations |
| Mediastinal gray-zone lymphoma | B-cell lymphoma, unclassifiable, with features intermediate between DLBCL and classic Hodgkin lymphoma | Mediastinal gray-zone lymphoma |
| **Burkitt lymphoma** | | |
| Burkitt lymphoma | Burkitt lymphoma | Burkitt lymphoma |
| **HHV-8–associated lymphoproliferative disorders** | **HHV-8–associated lymphoproliferative disorders** | **KSHV/HHV8–associated B-cell lymphoid proliferations and lymphomas** |
| Primary effusion lymphoma | Primary effusion lymphoma | Primary effusion lymphoma |
| Multicentric Castleman disease | Multicentric Castleman disease | (Listed under a new section – **Tumour-like lesions with B-cell predominance**) |
| HHV-8–positive diffuse large  B-cell lymphoma, NOS | HHV-8–positive diffuse large  B-cell lymphoma, NOS | KSHV/HHV8–positive diffuse large B-cell lymphoma |
| HHV-8–positive germinotropic lymphoproliferative disorder | HHV-8–positive germinotropic lymphoproliferative disorder | KSHV/HHV8–positive germinotropic lymphoproliferative  disorder |
| (Included in a separate **immunodeficiency-associated lymphoproliferative disorders** chapter – see section 5 below) | (Included in a separate **immunodeficiency-associated lymphoproliferative disorders** chapter – see section 5 below) | **Lymphoid proliferations and lymphomas associated with**  **immune deficiency and dysregulation** |
|  |  | Hyperplasias arising in immune deficiency/dysregulation |
|  |  | Polymorphic lymphoproliferative disorders arising in immune  deficiency/dysregulation |
| EBV-positive mucocutaneous ulcer | *EBV-positive mucocutaneous ulcer* | EBV-positive mucocutaneous ulcer |
|  |  | Lymphomas arising in immune deficiency/dysregulation |
|  |  | Inborn error of immunity-associated lymphoid proliferations and lymphomas |
| **Monoclonal gammopathies‡** | | |
| IgM monoclonal gammopathy of undetermined significance (MGUS) | IgM monoclonal gammopathy of undetermined significance | IgM monoclonal gammopathy of undetermined significance‡ |
| IgM MGUS, plasma cell type |  |  |
| IgM MGUS, NOS |  |  |
| Non-IgM monoclonal gammopathy of undetermined significance (Non-IgM MGUS) | Non-IgM monoclonal gammopathy of undetermined significance | Non-IgM monoclonal gammopathy of undetermined significance‡ |
|  |  | Monoclonal gammopathy of renal significance‡ |
| Primary cold agglutinin disease |  | Cold agglutinin disease‡ |
| **Heavy chain diseases‡** | | |
| Mu heavy chain disease | Mu heavy chain disease | Mu heavy chain disease‡ |
| Gamma heavy chain disease | Gamma heavy chain disease | Gamma heavy chain disease‡ |
| Alpha heavy chain disease | Alpha heavy chain disease | Alpha heavy chain disease‡ |
| **Plasma cell neoplasms‡** | | |
| Multiple myeloma (plasma cell myeloma) | Plasma cell myeloma | Plasma cell myeloma/ multiple myeloma‡ |
| Multiple myeloma (MM), NOS |  |  |
| MM with recurrent genetic abnormality |  |  |
| MM with *CCND* family translocation |  |  |
| MM with *MAF* family translocation |  |  |
| MM with *NSD2* translocation |  |  |
| MM with hyperdiploidy |  |  |
|  | Plasma cell myeloma variants |  |
|  | Smouldering (asymptomatic) plasma cell myeloma |  |
|  | Non-secretory myeloma |  |
|  | Plasma cell leukaemia |  |
|  | Plasmacytoma | Plasmacytoma‡ |
| Solitary plasmacytoma of bone | Solitary plasmacytoma of bone | Solitary plasmacytoma of bone |
| Extraosseous plasmacytoma | Extraosseous plasmacytoma | Extramedullary plasmacytoma |
|  | Plasma cell neoplasms with associated paraneoplastic syndrome   - POEMS syndrome - *TEMPI syndrome* | Plasma cell neoplasms with associated paraneoplastic syndrome‡   - POEMS syndrome - TEMPI syndrome - AESOP syndrome |
| **Monoclonal immunoglobulin deposition diseases** | **Monoclonal immunoglobulin deposition diseases** | **Diseases with monoclonal immunoglobulin deposition‡** |
| Ig light chain (AL) amyloidosis | Primary amyloidosis | Immunoglobulin-related (AL) amyloidosis‡ |
| Localised AL amyloidosis |  |  |
| Light chain and heavy chain deposition diseases | Light chain and heavy chain deposition diseases | Monoclonal immunoglobulin deposition disease‡ |

Italic font indicates provisional tumour entities.

Updated since WHO 2017 (terminology/wording aligned between ICC 2022 and WHO 2022)

Updated since WHO 2017 (terminology/wording different between ICC 2022 and WHO 2022)

Minimal or no changes since WHO 2017

**Key**

AESOP, adenopathy and extensive skin patch overlying a plasmacytoma; ALK, anaplastic lymphoma kinase; EBV, Epstein–Barr virus; HHV-8, human herpesvirus-8, KSHV, Kaposi sarcoma herpesvirus; LPL, lymphoplasmacytic lymphoma; NOS, not otherwise specified; POEMS, polyneuropathy, organomegaly, endocrinopathy, M-protein and skin changes; TEMPI, telangiectasias, elevated erythropoietin and erythrocytosis, monoclonal gammopathy, perinephric fluid collections, and intrapulmonary shunting.

* Previously termed ‘nodular lymphocyte-predominant Hodgkin lymphoma’ and included in the Hodgkin lymphoma section.

† Not included as one of the main families of mature B-cell neoplasm in the WHO 2022 classification.

‡ Not included as mature B-cell neoplasm in the WHO 2022 classification (as is in the WHO 2017 blue book) but included instead under a new section – plasma cell neoplasms and other diseases with paraproteins.

**ICC 2022 changes within the mature B-cell neoplasms section**

- Primary cutaneous marginal zone lymphoproliferative disorder recognised as a distinct entity to be segregated from other mucosa-associated lymphoid tissue lymphomas
- Testicular follicular lymphoma recognised as a distinct form of follicular lymphoma in young boys
- *BCL2-R-negative, CD23-positive follicle centre lymphoma* added as a new provisional entity
- Nodular lymphocyte predominant B-cell lymphoma added as a new entity (previously termed nodular lymphocyte-predominant Hodgkin lymphoma and included within the Hodgkin lymphoma section)
- Primary diffuse large B-cell lymphoma of the testis recognised as a new, specific entity closely related to primary diffuse large B-cell lymphoma of the central nervous system
- Large B-cell lymphoma with *IRF4* rearrangement upgraded to a definite entity and listed in sequence with follicular lymphomas, based on its common follicular pattern, and clinical features distinct from DLBCL
- *HHV-8 and EBV-negative primary effusion-based lymphoma* recognised as a provisional entity frequently associated with fluid overload
- EBV-positive polymorphic B-cell lymphoproliferative disorder, NOS added as a new entity
- High-grade B-cell lymphoma with *MYC* and *BCL2* and/or *BCL6* rearrangements separated into two distinct entities:
  - High-grade B-cell lymphoma with *MYC* and *BCL2* rearrangement
  - *High-grade B-cell lymphoma with MYC and BCL6 rearrangements* *(provisional)*
- *Burkitt-like lymphoma with 11q aberration* replaced by *large B-cell lymphoma with 11q aberration*
- B-cell lymphoma, unclassifiable, with features intermediate between DLBCL and classic Hodgkin lymphoma replaced with a new term, ‘mediastinal gray-zone lymphoma’
- EBV-positive mucocutaneous ulcer now recognised as a definite entity
- IgM MGUS, plasma cell type and IgM MGUS, NOS added as new subtypes of IgM MGUS
- Primary cold agglutinin disease added as a new entity
- The term ‘multiple myeloma’ (MM) replaces plasma cell myeloma
  - MM is designated as NOS or ‘MM with recurrent cytogenetic abnormalities’, the latter of which is subclassified into four mutually exclusive cytogenetic groups
- Plasma cell myeloma variants omitted as a separate entity but recognised as variants under multiple myeloma
- Plasma cell neoplasms with associated paraneoplastic syndrome omitted from document because they are considered clinical manifestations of the same disease, not distinct entities
- Primary amyloidosis replaced with Ig light chain (AL) amyloidosis and separated from localised AL amyloidosis (also termed ‘amyloid tumour’)

**WHO 2022 changes within the mature B-cell neoplasms section**

- The WHO 2022 document outlines 12 families of mature B-cell neoplasm (in bold black text in Table 1):
  - Pre-neoplastic and neoplastic small lymphocytic proliferations
  - Splenic B-cell lymphomas and leukaemias
  - Lymphoplasmacytic lymphoma
  - Marginal zone lymphoma
  - Follicular lymphoma
  - Cutaneous follicle centre lymphoma
  - Mantle cell lymphoma
  - Transformations of indolent B-cell lymphomas
  - Large B-cell lymphomas
  - KSHV/HHV8-associated B-cell lymphoid proliferations and lymphomas
  - Burkitt lymphoma
  - Lymphoid proliferations and lymphomas associated with immune deficiency and dysregulation
- Monoclonal gammopathies, diseases with monoclonal immunoglobulin deposition, heavy chain diseases and plasma cell neoplasms fall under a new section named ‘plasma cell neoplasms and other diseases with paraproteins’
- B-cell prolymphocytic leukaemia omitted as an entity
- Splenic B-cell lymphoma/leukaemia, unclassifiable omitted as an entity
  - The previously provisional subtype ‘splenic diffuse red pulp small B-cell lymphoma’ is recognised as a distinct entity
  - The previously provisional subtype ‘hairy cell leukaemia variant’ is recognised as a distinct entity and renamed ‘splenic B-cell lymphoma/leukaemia with prominent nucleoli’. This entity also encompasses cases of previously termed ‘CD5-negative B-prolymphocytic leukaemia’
- Two subtypes of lymphoplasmacytic lymphoma are recognised – IgM LPL/Waldenström macroglobulinemia type and non-IgM LPL/Waldenström macroglobulinemia type
- Primary cutaneous marginal zone lymphoma added as a new entity
- In situ follicular neoplasia renamed to in situ follicular B-cell neoplasm
- Testicular follicular lymphoma included under follicular variants
- Transformations of indolent B-cell lymphomas added as a new family
- In situ mantle cell neoplasia renamed to in situ mantle cell neoplasm
- Primary large B-cell lymphoma of immune-privileged sites added as a new umbrella term to encompass primary diffuse large B-cell lymphoma of the CNS, primary large B-cell lymphoma of the vitreoretina and primary large B-cell lymphoma of the testis
- Large B-cell lymphoma with *IRF4* rearrangement, upgraded to a definite entity and included with large B-cell lymphomas
- Fluid overload-associated large B-cell lymphoma added as a new entity
- ‘NOS’ qualifier dropped from EBV-positive diffuse large B-cell lymphoma
- Fibrin-associated large B-cell lymphoma added as a distinct entity (previously a subtype of diffuse large B-cell lymphoma associated with chronic inflammation)
- HHV-8–associated lymphoproliferative disorders renamed to KSHV/HHV-8–associated B-cell lymphoid proliferations and lymphomas
  - Multicentric Castleman disease included under a new section – tumour-like lesions with B-cell predominance, instead of under mature B-cell neoplasms
  - HHV-8–positive DLBCL, NOS renamed to KSHV/HHV-8–positive DLBCL
  - HHV-8–positive germinotropic lymphoproliferative disorder renamed to KSHV/HHV-8–positive germinotropic lymphoproliferative disorder
- High-grade B-cell lymphoma with *MYC* and *BCL2* and/or *BCL6* rearrangements conceptually reframed with diffuse large B-cell lymphoma/high-grade B-cell lymphoma with *MYC* and *BCL2* rearrangements as an entity and diffuse large B-cell lymphoma/high-grade B-cell lymphoma with *MYC* and *BCL6* rearrangements as a genetic subtype of diffuse large B-cell lymphoma, NOS or high-grade B-cell lymphoma, NOS with *MYC* and *BCL6* rearrangements
- *Burkitt-like lymphoma with 11q aberration* renamed to high-grade B-cell lymphoma with 11q aberrations
- B-cell lymphoma, unclassifiable, with features intermediate between DLBCL and classic Hodgkin lymphoma replaced with a new term, ‘mediastinal gray-zone lymphoma’
- Lymphoid proliferations and lymphomas associated with immune deficiency and dysregulation included as a family of mature B-cell neoplasms (previously included under a separate section – immunodeficiency-associated lymphoproliferative disorders)

**WHO 2022 plasma cell neoplasms and other diseases with paraproteins entity changes**

- Monoclonal gammopathy of renal significance and cold agglutinin disease added as two new entities of monoclonal gammopathies
- Extraosseous plasmacytoma subtype renamed to extramedullary plasmacytoma
- AESOP syndrome added as a third subentity of plasma cell neoplasms with associated paraneoplastic syndrome
- Primary amyloidosis renamed to immunoglobulin-related (AL) amyloidosis
- Light chain and heavy chain deposition disease renamed to monoclonal immunoglobulin deposition disease

## Pre-neoplastic and neoplastic small lymphocytic proliferations

The ICC 2022 and WHO 2022 classifications have provided updated information on pre-neoplastic and neoplastic small lymphocytic proliferations since the WHO 2017 blue book was published.

### 2.2.1 Chronic lymphocytic leukaemia/small lymphocytic lymphoma (CLL/SLL)

CLL/SLL is a neoplasm composed of monomorphic small mature B cells that frequently co-express CD5 and CD23. The WHO 2017 blue book stated that “there must be a monoclonal B-cell count ≥5 x 10^9^/L, with the characteristic morphology and phenotype of CLL in the peripheral blood.” Despite CLL and SLL being considered the same disease, the term SLL is used when there are <5 x 10^9^/L circulating B cells and nodal, splenic, or other extramedullary involvement.

The diagnostic criteria for CLL are well established and are aligned across the WHO 2017 blue book, the WHO 2022 document and the ICC 2022 document. All three documents highlight CD19, CD5, CD20, CD23, kappa, and lambda as essential markers for diagnosis and CD43, CD79b, CD81, CD200, CD10, and ROR1 as useful markers to clarify the diagnosis.

The main updates in the ICC 2022 and WHO documents since 2017 relate to the recommended investigations for prognosis/prediction of CLL (Table 2).

Table 2. Wording updates relating to prognosis/prediction of CLL

| **ICC 2022** | **WHO 2017** | **WHO 2022** |
| --- | --- | --- |
| - The mutational status of the *IGHV* and *TP53*/17p alterations need to be evaluated at the time when patients require treatment - Factors likely to have significant clinical relevance include subclonal *TP53* mutations with low variant allelic frequency (<10%), BCR stereotypes (e.g. stereotypes 2 and 8), specific mutated genes (e.g. *NOTCH1*, *SF3B1*, and *BIRC3*), the IGLV3-21^R110^ mutation and complex karyotype, defined as  ≥3 aberrations (although in CLL, a distinct threshold of ≥5 abnormalities may better stratify very-high-risk patients) | - *TP53* abnormalities (i.e. deletion in 17p13 and *TP53* mutations) are predictive of lack of response to fludarabine-containing regimens; therefore, these aberrations should be checked for in all patients before starting any line of therapy - Patients with mutated *IGHV* genes have a better prognosis than do those with unmutated genes | - *TP53* mutational analysis,   *IGHV* region somatic hypermutation analysis and BCR stereotype subset analysis (subset #2 configuration) are recommended for full prognostic evaluation of CLL/SLL   - Detection of karyotypic complexity and *BTK*, *PLCG2*, and *BCL2* mutation status all remain desirable additional investigations in the context of targeted therapy |
| - Reversible Richter-like proliferations in patients in which a BTK inhibitor has been interrupted must be distinguished from DLBCL transformation - The ICC uses the term ‘Richter transformation’ | - Approximately 2–8% of patients with CLL develop Richter syndrome (DLBCL) | - In the setting of transformation, use of the term ’Richter transformation’ is recommended over ’Richter syndrome’ |

BCR, B-cell receptor; BTK, Bruton´s tyrosine kinase.

### 2.2.2 Monoclonal B-cell lymphocytosis (MBL)

MBL is defined by a monoclonal B-cell count <5 x 10^9^/L in the peripheral blood in subjects who have no associated lymphadenopathy, organomegaly, other extramedullary involvement or any other feature of a B-cell lymphoproliferative disorder.

The WHO 2017 blue book, ICC 2022 classification and the WHO 2022 classification recognise different subtypes of MBL (Table 3).

Table 3. Subtypes of MBL

| **ICC 2022** | **WHO 2017** | **WHO 2022** |
| --- | --- | --- |
| CLL-type  Characterised by  co-expression of CD19, CD5, CD23 and CD20 (dim). The  B cells show light chain class restriction or ≥25% lack surface  immunoglobulin | CLL-type  Characterised by  co-expression of CD19, CD5, CD23 and CD20 (dim). The  B cells show light chain class restriction or ≥25% lack surface  immunoglobulin | CLL/SLL-type  Monoclonal CLL/SLL-phenotype* B-cell count ≥0.5 x 10^9^/L and total B-cell count <5 x 10^9^/L  with no other features diagnostic of CLL/SLL |
| Non-CLL-type  Characterised by CD5- (or CD5 [dim] in 20% of cases), CD19+, CD20+ B cells with  moderate to bright surface immunoglobulin expression | Non-CLL-type  Characterised by CD5- (or CD5 [dim] in 20% of cases), CD19+, CD20+ B cells with  moderate to bright surface immunoglobulin expression | Non-CLL/SLL type  Any monoclonal non-CLL/SLL  phenotype B-cell expansion with no symptoms or features  diagnostic of another mature  B-cell neoplasm. The majority  of cases have features consistent with a marginal zone origin |
| Atypical CLL-type  CD5+/bright CD20+/variable CD23+/bright surface immunoglobulin  Tissue-based MBL  An incidental nodal or extranodal finding of an infiltrate of CLL-type cells without proliferation centres in individuals without significant lymphadenopathy | Atypical CLL-type  Characterised by  co-expression of CD19, CD5, CD20 (bright), and moderate to bright surface immunoglobulin. CD23 may be negative | Low-count MBL or  clonal B-cell expansion  Clonal CLL/SLL phenotype B-cell count <0.5 x 10^9^/L with no other features diagnostic of  B-lymphoproliferative disorder |

* The CLL/SLL phenotype is defined as co-expression of CD5 and CD23 on >20% of the clonal CD19+ B cells with weak expression of CD20 and immunoglobulin light chain and no CD10 expression.

### 2.2.3 B-cell prolymphocytic leukaemia (B-PLL)

B-PLL is very rare and is characterised by the clonal proliferation of B-cell prolymphocytes, primarily affecting the peripheral blood, bone marrow and spleen.

In contrast to the WHO 2017 blue book, the WHO 2022 classification does not recognise B-PLL as an entity of mature B-cell neoplasm due to its heterogeneous nature. Some cases of CD5- B-PLL are absorbed by the new entity ‘splenic B-cell lymphoma/leukaemia with prominent nucleoli’ (see below). The ICC 2022 classification does recognise B-PLL but states that “the entity only needs to be recognised after rigorous exclusion of other lymphoid neoplasms, particularly transformation from CLL, mantle cell lymphoma or splenic marginal zone lymphoma.”

## Splenic B-cell lymphomas and leukaemias

Splenic B-cell lymphomas and leukaemias are a group of disorders characterised by the proliferation of B cells primarily in the spleen, bone marrow and peripheral blood.

The WHO 2017 blue book and the ICC 2022 classification recognise splenic marginal zone lymphoma, hairy cell leukaemia and the provisional category, splenic B-cell lymphoma/leukaemia, unclassifiable. Two provisional entities, splenic diffuse red pulp small B-cell lymphoma and hairy cell leukaemia variant, are listed under splenic B-cell lymphoma/leukaemia. As mentioned in section 2.1, in the WHO 2022 classification, the term ‘splenic B-cell lymphoma/leukaemia, unclassifiable’ has been omitted and replaced with two separate entities – splenic diffuse red pulp small B-cell lymphoma and splenic B-cell lymphoma/leukaemia with prominent nucleoli, the latter replacing the term ‘hairy cell leukaemia variant’ in the WHO 2017 blue book.

There have been no other major updates to splenic B-cell lymphoma and leukaemia entities since 2017.

## Lymphoplasmacytic lymphoma (LPL) and Waldenström macroglobulinaemia

LPL is a neoplasm of small B lymphocytes, plasmacytoid lymphocytes and plasma cells, usually involving bone marrow and sometimes lymph nodes and spleen. Waldenström macroglobulinaemia (WM) is found in a substantial subset of patients with LPL but is not synonymous with it; it is defined by the combination of LPL in the bone marrow with an IgM monoclonal component in the blood.

The 2017 WHO blue book and ICC 2022 classification distinguish between LPL and WM, whereas the WHO 2022 classification recognises two subtypes of LPL: IgM LPL/WM type and non-IgM LPL/WM type. The ICC 2022 and WHO 2022 classifications have also provided updated information on the diagnosis of LPL since the WHO 2017 blue book was published (Table 4).

Table 4. Wording updates relating to diagnosis of LPL

| **ICC 2022** | **WHO 2017** | **WHO 2022** |
| --- | --- | --- |
| - Immunophenotype of LPL typically IgM+, CD19+, CD20+, CD22+, CD79a+, CD25+ CD38+, CD5-,  CD10-, CD103- and CD23- - Diagnosis may be made with lymphoplasmacytic aggregates in trephine biopsies <10% of cellularity with evidence of clonal B cells and plasma cells | - LPL cells are typically IgM+, CD19+, CD20+, CD22+, CD79a+, CD25+ CD38+, CD5-, CD10-, CD103- and CD23- | Essential diagnostic criteria:   - Bone marrow infiltration by >10% small lymphocytes with plasmacytoid and/or plasma cell differentiation - Immunophenotype of LPL cells: IgM+, CD19+, CD20+, CD22+, CD25+, CD10-, CD23-, CD103-, CD138+/- |
| - Molecular studies for *MYD88* and *CXCR4*  mutations are strongly encouraged in the workup of suspected lymphoplasmacytic lymphoma | - The great majority (>90%) of LPLs have *MYD88* L265P, and approximately 30% have truncating *CXCR4* mutations | Desirable diagnostic criteria:   - Detection of *MYD88* (NP_002459.2:p.L265P) mutation - Detection of *CXCR4* somatic mutation - Serum electrophoresis and immunofixation showing presence of monoclonal IgM |

## Marginal zone lymphomas (MZLs)

MZLs are a heterogeneous group of indolent B-cell non-Hodgkin lymphomas that arise from the marginal zone of the lymphoid tissue. MZLs can occur in various organs and tissues, including the lymph nodes, spleen, gastrointestinal tract and other mucosal sites, such as the lung or salivary glands.

The WHO 2017 blue book recognised three main entities of MZL: extranodal marginal zone lymphoma (ENMZL), nodal marginal zone lymphoma (NMZL) and splenic marginal zone lymphoma (SMZL; see section 2.3). The ICC 2022 and WHO 2022 documents also recognise primary cutaneous marginal zone lymphoma/lymphoproliferative disorder as another distinct entity of MZL. In addition, the WHO 2022 classification lists paediatric nodal marginal zone lymphoma (PNMZL) as a separate entity to NMZL.

There have been no major updates to ENMZL, NMZL or PNMZL since 2017.

### 2.5.1 Primary cutaneous marginal zone lymphoma/lymphoproliferative disorder

The ICC 2022 and WHO 2022 classifications recognise two subtypes of primary cutaneous marginal zone lymphoma/lymphoproliferative disorder: heavy chain class-switched form (IgG+, IgA+ or IgE+) or non-class-switched form (IgM+).

Both classifications also highlight the need to exclude non-cutaneous primary disease at the time of diagnosis.

## Follicular lymphoma (FL)

FL is one of the more common lymphomas. The vast majority of FLs have at least a partially follicular growth pattern and are composed of centrocytes and centroblasts.

The WHO 2017 blue book defined four grades of FL: grade 1, grade 2, grade 3A and grade 3B. FL is graded by assessing the number of centroblasts per 10 high-power-fields (HPF) using a 40x objective. The ICC 2022 classification maintains this grading system but adds the following information:

- In equivocal cases of follicular lymphoma grade 3, *BCL2* rearrangement and CD10 positivity both favour grade 3A over grade 3B
- Patients with follicular lymphoma grade 3B with IRF4/MUM1 expression should be evaluated for*IRF4* alteration, especially younger patients
- Routine molecular testing is currently not required, but it can be useful in selected patients for differential diagnosis and specific therapeutic options (e.g. EZH2 inhibitors)

In the WHO 2022 classification, grading of FL is optional.

Since 2017 there have been updates to the variants/subtypes of FL. The WHO 2017 blue book recognised four variants of FL: in situ follicular neoplasia, duodenal-type FL, testicular FL and the diffuse variant of FL. Paediatric-type FL was considered a separate entity. The ICC 2022 document recognises two subtypes of FL: in situ follicular neoplasia and duodenal-type FL. Both paediatric-type FL and testicular FL are considered separate entities and a new provisional entity is proposed – *BCL2-R–negative, CD23-positive follicle centre lymphoma*. In the WHO 2022 document, the family of follicular lymphoma encompasses four entities: follicular lymphoma (FL), in situ follicular B-cell neoplasm, paediatric-type FL and duodenal-type FL. The WHO 2022 classification also recognises four subtypes within the FL entity:

- Classic FL (cFL): FL with at least in part a follicular growth pattern, composed of centrocytes and centroblasts and harbouring the t(14;18)(q32;q21) translocation associated with *IGH*::*BCL2* fusion in 85% of cases
- Follicular large B-cell lymphoma (FLBL): formerly FL grade 3B; has a close clinical and biological relationship to DLBCL
- FL with unusual cytological features (ucFL) – ’blastoid’ or ’large centrocyte’ variants*
- FL with predominantly diffuse growth pattern (dFL)*

There have been no major updates to in situ follicular neoplasia/in situ follicular B-cell neoplasm, duodenal-type FL or paediatric-type FL since 2017. Testicular FL is only recognised as an entity in the ICC 2022 classification, not in the WHO 2022 classification.

*These entities are provided in the 5th edition (beta v2) available online and vary from the Aleggio R, et al. Leukemia 2022 paper.

### 2.6.1 BCL2-R–negative, CD23-positive follicle centre lymphoma

The ICC 2022 classification recognises a provisional entity, *BCL2-R-negative, CD23-positive follicle centre lymphoma*, as a specific form of follicle centre lymphoma, frequently but not always with a diffuse pattern, frequent pelvic/inguinal location, and common *STAT6* mutations. This entity is partly covered in the WHO 2022 classification* within the dFL subtype.

*The 5th edition (beta v2) available online.

## Primary cutaneous follicle centre lymphoma

Primary cutaneous follicle centre lymphoma is a tumour of neoplastic follicle centre cells, including centrocytes and variable numbers of centroblasts, with a follicular, follicular and diffuse or diffuse growth pattern that generally presents in the skin of the head or trunk.

There have been no major updates to this entity since 2017. The ICC 2022 document states that “molecular and cytogenetic studies further support its segregation from other follicular lymphomas and may help predict subsequent extracutaneous dissemination.“

## Transformations of indolent B-cell lymphomas

The WHO 2022 classification has introduced transformations of indolent B-cell lymphomas as a new family/class. Transformation is defined as the emergence of an aggressive lymphoma type in a patient with previously or synchronously diagnosed, clonally-related indolent B-cell lymphoma.

## Mantle cell lymphoma (MCL)

MCL is a mature B-cell neoplasm derived from cells of the mantle zone of lymphoid follicles, and is typically composed of small to medium-sized monomorphic cells expressing CD5, SOX11 and cyclin D1. It is associated with *CCND* family rearrangements, most commonly *CCND1*. The ICC 2022 and WHO 2022 classifications provide more information on the definition of MCL (Table 5).

Table 5. Updated information on the definition of MCL

| **ICC 2022** | **WHO 2017** | **WHO 2022** |
| --- | --- | --- |
| - Definition is expanded to include genetic variants with *CCND2* and *CCND3*  rearrangements with immunoglobulin genes in otherwise typical MCL - Aggressive B-cell lymphomas with secondary *CCND1*  rearrangements should not be diagnosed as MCL | - In >95% of cases, there is a *CCND1* translocation | - The *IGH*::*CCND1* fusion associated with t(11;14)(q13;q32) is the genetic hallmark of MCL, present in ≥95% of cases. In the small subset of MCL cases negative for cyclin D1 expression and *CCND1* rearrangement, *CCND2* or *CCND3* rearrangements have been identified as alternative mechanisms of cell cycle dysregulation |

The WHO 2017 blue book recognised two variants of MCL: in situ mantle cell neoplasia and leukaemic non-nodal MCL. There have been no major updates to these variants since 2017 except for the name change to in situ mantle cell neoplasm in the WHO 2022 classification.

## Large B-cell lymphomas

The large B-cell lymphomas family comprises a spectrum of tumours with varying morphologies, genetic features and clinical behaviour. Some large B-cell lymphoma entities have been updated since the WHO 2017 blue book, and some new entities have been added (as described in section 2.1).

Further information on the new/updated entities can be found in sections 2.10.1 to 2.10.8 below. The rest of the entities of large B-cell lymphoma remain largely unchanged since 2017, with the exception of some modifications in names/diagnostic terms.

### 2.10.1 Diffuse large B-cell lymphoma, NOS (DLBCL, NOS)

DLBCL, NOS is a neoplasm of medium or large B-lymphoid cells with a diffuse growth pattern. This is a morphologically and molecularly heterogeneous entity that does not meet the diagnostic criteria of specific large B-cell lymphoma neoplasms.

On the basis of gene expression profiling, DLBCL can be divided into two main molecular subtypes: the germinal centre B-cell-like (GCB) subtype and the activated B-cell-like (ABC) subtype. This cell-of-origin designation in DLBCL, NOS is consistent across the WHO 2017 blue book, ICC 2022 classification and WHO 2022 classification. However, it is highlighted in both the ICC 2022 and WHO 2022 documents that cell-of-origin classification is considered insufficient to fully capture the biological complexity of these tumours. Molecular profiling studies have identified new genetic subgroups of DLBCL that may provide more precise patient stratification in the future.

### 2.10.2 Nodular lymphocyte predominant B-cell lymphoma

In the ICC 2022 classification, nodular lymphocyte predominant B-cell lymphoma replaces nodular lymphocyte-predominant Hodgkin lymphoma, due to major biological and clinical differences from classic Hodgkin lymphoma and the close relationship to T-cell/histiocyte-rich large B-cell lymphoma. The WHO 2022 classification continues to use the Hodgkin lymphoma term but also accepts this new term. For more information, see section 3.2.

### 2.10.3 Primary diffuse large B-cell lymphoma of the central nervous system, testis and other immune-privileged sites

Primary diffuse large B-cell lymphoma of the central nervous system is defined as a DLBCL arising within the brain, spinal cord, leptomeninges or eye. In the WHO 2017 blue book and the ICC 2022 classification, it is recognised as a separate entity. The ICC 2022 document also recognises primary diffuse large B-cell lymphoma of the testis as a specific entity closely related to primary diffuse large B-cell lymphoma of the central nervous system.

The WHO 2022 classification introduces a new umbrella term, ‘primary large B-cell lymphoma of immune-privileged sites’, to encompass primary DLBCL of the CNS with primary DLBCL of the vitreoretina and primary DLBCL of the testis, which all share common biological features.

### 2.10.4 Fluid overload-associated large B-cell lymphoma

The WHO 2022 classification includes a new entity, ‘fluid overload associated large B-cell lymphoma (FO-LBCL)’, defined as a B-cell neoplasm presenting as serous effusions without detectable tumour masses, often in patients with fluid overload states. It is not associated with Kaposi sarcoma-associated herpes virus (KSHV)/Human herpesvirus 8 (HHV8); therefore, the WHO 2022 document recognises KSHV/HHV-8-negative effusion-based lymphoma as an acceptable alternative term. In the ICC 2022 classification, *HHV-8 and Epstein-Barr virus (EBV)-negative primary effusion-based lymphoma* is recognised as a provisional entity. The document states that “patients who conform to other well-defined lymphomas should not be included in this entity.” In contrast with the ICC 2022 classification, the WHO 2022 classification accepts rare cases of FO-LBCL associated with EBV infection outside overt immunodeficiency states (e.g. in putative immunosenescence)*.

*Additional information on this topic can be found in the 5th edition (beta v2) available online.

### 2.10.5 EBV-positive polymorphic B-cell lymphoproliferative disorder, NOS

The ICC 2022 classification introduces this as a new term to be used for EBV-associated B-cell proliferations with or without known immunodeficiency when the morphologic changes do not fulfil the criteria of a well-defined EBV-positive DLBCL, NOS. In patients with focal EBV-positive B cells and preserved lymph node architecture, the term ‘EBV reactivation’ is recommended.

### 2.10.6 High-grade B-cell lymphoma (HGBL or HGBCL)

HGBL (WHO) or HGBCL (ICC 2022) describes a group of aggressive, mature B-cell lymphomas that for biological and clinical reasons should not be classified as DLBCL, NOS or Burkitt lymphoma.

The WHO 2017 blue book recognised two entities: HGBL with *MYC* and *BCL2* and/or *BCL6* rearrangements and HGBL, NOS. The ICC 2022 classification maintains the HGBCL, NOS entity but has redefined the other entity, recognising HGBCL with *MYC* and *BCL2* rearrangements (with or without *BCL6* rearrangement) and excluding patients with only *MYC* and *BCL6* rearrangements. HGBCL with *MYC* and *BCL6* rearrangements remains recognised as a new provisional entity in an effort to encourage additional investigation into these cases. The WHO 2022 classification also maintains the HGBL, NOS subtype. However, owing to the fact that most *MYC/BCL2* ‘double hit’ lymphomas have a DLBCL morphology, HGBL with *MYC* and *BCL2* and/or *BCL6* rearrangements has been conceptually reframed and renamed diffuse large B-cell lymphoma/high-grade B-cell lymphoma (DLBCL/HGBL) with *MYC* and *BCL2* rearrangements. DLBCL and HGBL with *MYC* and *BCL6* rearrangements are considered genetic subtypes of DLBCL, NOS or HGBL, NOS, respectively.

### 2.10.7 High-grade B-cell lymphoma / large B-cell lymphoma with 11q aberration(s)

The WHO 2017 blue book coined the provisional entity ‘*Burkitt-like lymphoma with 11q aberration’*, identified originally as a lesion clinically and pathologically resembling Burkitt lymphoma but lacking *MYC* rearrangements. The ICC 2022 classification replaces this term with the provisional entity ‘*large B-cell lymphoma with 11q aberration*’ because molecular studies indicate that it is closer to DLBCL than to Burkitt lymphoma and its clinical behaviour is not as aggressive as other HGBLs. The WHO 2022 classification uses the term ‘high-grade B-cell lymphoma with 11q aberrations’ instead, because the tumour cells are not always large cell.

### 2.10.8 Mediastinal gray-zone lymphoma (MGZL)

The ICC 2022 and WHO 2022 classifications replace the term ‘B-cell-lymphoma, unclassifiable, with features intermediate between DLBCL and classic Hodgkin lymphoma’ (used in the WHO 2017 blue book) with MGZL. This entity describes a B-cell lymphoma with overlapping features between primary mediastinal large B-cell lymphoma and classic Hodgkin lymphoma, especially nodular sclerosis classic Hodgkin lymphoma. Clinical and genomic data indicate that most non-mediastinal gray-zone lymphomas are distinct from MGZL; thus, patients with extra-mediastinal disease should be diagnosed as having DLBCL, NOS.

## Burkitt lymphoma (BL)

BL is a highly aggressive but curable lymphoma that often presents in extranodal sites or as leukaemic disease. It is composed of monomorphic, medium-sized blastic B cells with basophilic cytoplasm and numerous mitotic figures, usually with a demonstrable *MYC* gene translocation to an IG locus. This definition remains largely unchanged since 2017.

In the WHO 2017 blue book, three subtypes of BL were recognised: endemic, non-endemic or sporadic and immunodeficiency-associated. However, the WHO 2022 classification advises characterising BL by molecular features rather than epidemiological criteria or geographic location. Therefore, the WHO 2022 document recommends differentiating BL into two subtypes: EBV-positive BL and EBV-negative BL, to reflect the dual mechanisms of BL pathogenesis – virus-driven and mutational-driven. EBV-positive cases show higher levels of somatic hypermutation, particularly in non-coding sequences near the transcription start site, and have fewer driver mutations, including those affecting *TCF3* and *ID3*.

Both 2022 classifications recommend that ‘Burkitt-like’ neoplasms with a precursor B-cell phenotype and a single *MYC* rearrangement are classified as B-lymphoblastic leukaemia/lymphoma with *MYC* rearrangement rather than BL.

## (KSHV/) HHV-8-associated lymphoproliferative disorders

In addition to causing Kaposi sarcoma, which may involve the lymph nodes, the human herpesvirus 8 (HHV8), also known as Kaposi sarcoma-associated herpesvirus (KSHV), is responsible for a spectrum of lymphoproliferative disorders. In the WHO 2022 classification, a new family has been introduced for KSHV/HHV-8–related lymphomas, and the full name KSHV/HHV-8 is now preferred over HHV-8 (which is used in the WHO 2017 blue book and ICC 2022 classification) to accommodate both the common practice of haematopathologists and of virus researchers.

There have been no major updates to these entities since 2017.

## Lymphoid proliferations and lymphomas associated with immune deficiency and dysregulation

In the WHO 2017 and ICC 2022 classifications, immunodeficiency-associated lymphoproliferative disorders are discussed in separate sections. However, the WHO 2022 classification organises lymphoid proliferations and lymphomas associated with immune deficiency as one of the 12 families of mature B-cell neoplasms. For more details on this class/family, see section 5 below.

### 2.13.1 EBV-positive mucocutaneous ulcer

EBV-positive mucocutaneous ulcer was introduced in the WHO 2017 blue book as a provisional entity occurring in patients with age-related or iatrogenic immunosuppression, often with Hodgkin-like features and a typically indolent course, with spontaneous regression in some cases. In the ICC 2022 and WHO 2022 classifications, it is now considered a definite entity.

There have been no major updates to EBV-positive mucocutaneous ulcer since 2017.

## Monoclonal gammopathies

Monoclonal gammopathies are a group of disorders characterised by a proliferation of either clonal plasma cells or lymphoid cells that produce a detectable monoclonal protein in the blood or urine. The ICC 2022 and WHO 2022 classifications have provided updated information on monoclonal gammopathies since the WHO 2017 blue book was published.

### 2.14.1 IgM monoclonal gammopathy of undetermined significance (IgM MGUS)

IgM MGUS is defined by the presence of a serum monoclonal (M) protein of <3 g/dL and bone marrow plasma cells <10% in patients who lack lymphoplasmacytic B-cell infiltrates/aggregates sufficient for a diagnosis of LPL.

The WHO 2017 blue book and WHO 2022 classification do not define subtypes of IgM MGUS. However, in the ICC 2022 classification two types of IgM MGUS are recognised – a plasma cell type and a NOS type. The rare plasma cell type is considered a precursor of IgM+ plasma cell myeloma/multiple myeloma (PCM/MM) and is defined as showing clonal plasma cells without a detectable B-cell component and with wild-type *MYD88*. This entity also includes patients with t(11;14)(q13;q32) or other cytogenetic abnormalities typical of PCM/MM. The remaining patients with IgM MGUS, NOS include those with a *MYD88* mutation, those with detectable monotypic or monoclonal B cells but without abnormal lymphoplasmacytic aggregates diagnostic of LPL and those who lack evidence of other small B-cell neoplasms.

The ICC 2022 document recommends routine fluorescence in situ hybridisation (FISH) studies and *MYD88* mutation analysis to identify the rare tumours that are likely to progress to PCM/MM.

### 2.14.2 Non-IgM monoclonal gammopathy of undetermined significance (Non-IgM MGUS)

Non-IgM MGUS is defined by the presence of a non-IgM (mainly IgG or IgA, rarely IgE, IgD and light chain only) serum monoclonal (M) protein of <3 g/dL, bone marrow plasma cells <10%, and the absence of end-organ damage, such as hypercalcaemia, renal insufficiency, anaemia or bone lesions that can be attributed to the plasma cell proliferative disorder.

In the WHO 2017 blue book and the ICC 2022 classification, non-IgM MGUS is included as a plasma cell neoplasm. However, in the WHO 2022 classification it has been grouped with other monoclonal gammopathies because it is a precursor lesion with the potential to evolve to a plasma cell neoplasm.

There have been no other updates to non-IgM MGUS since 2017.

### 2.14.3 Monoclonal gammopathy of renal significance (MGRS)

MGRS is a plasma cell or B-cell neoplasm that does not meet the accepted criteria for malignancy but secretes a monoclonal immunoglobulin or fragment, resulting in kidney damage and dysfunction.

In contrast to the WHO 2017 blue book and the ICC 2022 classification, the WHO 2022 classification introduces MGRS as a new distinct entity. The ICC 2022 document recognises MGRS and monoclonal gammopathy of clinical significance (MGCS), but not as separate disease entities; instead, MGRS and MGCS are viewed as descriptive terms that can be added as a clinical feature to the diagnosis of the underlying disease (e.g. MGUS).

### 2.14.4 (Primary) cold agglutinin disease

The ICC 2022 and WHO 2022 classifications recognise (primary) cold agglutinin disease as a new diagnostic entity, distinct from LPL or IgM MGUS. It is a rare autoimmune haemolytic anaemia mediated by monoclonal cold agglutinins and driven by an underlying clonal B-cell lymphoid proliferation not fulfilling criteria for a B-cell lymphoma.

## Heavy chain diseases

The heavy chain diseases (HCDs) are three rare B-cell neoplasms characterised by the production of monoclonal immunoglobulin heavy chains (IgG in gamma HCD, IgA in alpha HCD, and IgM in mu HCD) and typically no light chains.

There have been no major updates to HCDs since 2017.

## Plasma cell neoplasms

Plasma cell neoplasms arise from the monoclonal expansion of post-germinal centre, class switched, terminally differentiated B cells that secrete monoclonal immunoglobulins called M-proteins or paraproteins. The ICC 2022 and WHO 2022 classifications have provided updated information on plasma cell neoplasms since the WHO 2017 blue book was published.

### 2.16.1 Plasma cell myeloma (PCM) / multiple myeloma (MM)

In the WHO 2017 blue book the term ‘plasma cell myeloma’ is used but multiple myeloma is highlighted as a synonym. The WHO 2022 classification defines the two terms separately:

- Plasma cell myeloma is a bone marrow-based, multifocal neoplastic proliferation of plasma cells
- Multiple myeloma is defined by the combination of plasma cell myeloma, usually associated with a serum and/or urine monoclonal Ig, and either evidence of organ damage related to the disease or, in the absence of organ damage, laboratory or imaging findings that suggest a high risk of developing end-organ damage within 2 years

In the ICC 2022 classification, the term ‘multiple myeloma’ is preferred over plasma cell myeloma.

The ICC document also divides multiple myeloma into two main groups defined by cytogenetics: MM, NOS and MM with recurrent genetic abnormalities, including MM with *CCND* family translocations, MM with *MAF* family translocation, MM with *NSD2* translocation, and MM with hyperdiploidy.

While these genetic variants are mentioned, but not explicitly stated as subtypes in the WHO 2022 classification, the latter still recognises ‘plasma cell myeloma variants’ as separate subtypes, which include smouldering (asymptomatic) PCM, non-secretory myeloma and plasma cell leukaemia. The ICC 2022 classification recognises the same variants as subtypes of PCM.

### 2.16.2 Plasmacytoma

Plasmacytoma is a solitary neoplasm of clonal plasma cells without evidence of plasma cell myeloma or end-organ damage due to plasma cell neoplasia. There are two types of plasmacytoma: solitary plasmacytoma of bone and extraosseous (extramedullary) plasmacytoma.

Both the ICC and WHO 2022 classifications recommend studying the presence of plasma cells in the bone marrow by flow cytometry in plasmacytoma entities, particularly in solitary plasmacytomas of bone, due to its prognostic impact. There have been no other major updates to this entity since 2017.

### 2.16.3 Plasma cell neoplasms with associated paraneoplastic syndrome

This is a group of rare paraneoplastic syndromes associated with plasma cell neoplasms. The WHO 2017 blue book recognised POEMS syndrome and the provisional entity ‘TEMPI syndrome’ as part of this family/class.

The WHO 2022 classification confirms TEMPI syndrome as a distinct subtype of plasma cell neoplasms with associated paraneoplastic syndrome. In addition, AESOP syndrome has been added as another subtype. The ICC 2022 classification has omitted the plasma cell neoplasms with associated paraneoplastic syndrome family/class, regarding them as clinical variants in the setting of plasma cell neoplasms.

## Monoclonal immunoglobulin (Ig) deposition diseases

The monoclonal Ig deposition diseases are closely related disorders characterised by visceral and soft tissue deposition of aberrant Ig, resulting in compromised organ function. The WHO 2017 blue book classified monoclonal Ig deposition diseases as plasma cell neoplasms, whereas they are grouped separately in the ICC 2022 and WHO 2022 classifications.

The WHO 2017 blue book recognised two major categories of monoclonal Ig deposition diseases: primary amyloidosis and light chain and heavy chain diseases, both of which have been renamed in the ICC 2022 and WHO 2022 classifications.

### 2.17.1 Primary amyloidosis

In the WHO 2022 classification, primary amyloidosis is renamed to immunoglobulin-related (AL) amyloidosis. In the ICC 2022 classification, it is renamed to Ig light chain (AL) amyloidosis. The ICC 2022 document also emphasises the need to distinguish this entity from localised AL amyloidosis, a rare disorder with excellent prognosis and rare progression to systemic AL amyloidosis.

There have been no other updates since 2017.

### 2.17.2 Light chain and heavy chain deposition diseases

In the WHO 2022 classification, this term is renamed to monoclonal Ig deposition disease. There have been no other updates since 2017.

# 3. Hodgkin lymphomas

Hodgkin lymphomas (HLs) are lymphoid neoplasms usually affecting lymph nodes. They are composed of large dysplastic mononuclear and multinucleated cells surrounded by a variable mixture of mature non-neoplastic inflammatory cells. Abundant band-like and/or more diffuse collagen fibrosis may be present. The neoplastic cells are often ringed by T cells in a rosette-like manner.

## 3.1 Categorisation

Two major categories of HL were recognised in the WHO 2017 blue book: nodular lymphocyte predominant HL (NLPHL) and classic HL (CHL). The updated WHO 2022 document retains these two main HL categories; however, the ICC 2022 classification replaces NLPHL with the term ‘nodular lymphocyte predominant B-cell lymphoma’ and lists it under mature B-cell neoplasms. The entities included within the HL section of each document are listed in Table 6.

Table 6. Hodgkin lymphoma entities

| **ICC 2022** | **WHO 2017** | **WHO 2022** |
| --- | --- | --- |
| (Included as ‘Nodular lymphocyte predominant B-cell lymphoma’ and listed under **Mature B cell neoplasms** – see section 1 above) | Nodular lymphocyte predominant Hodgkin lymphoma | Nodular lymphocyte predominant Hodgkin lymphoma |
| Classic Hodgkin lymphoma | Classic Hodgkin lymphoma | Classic Hodgkin lymphoma |
| Nodular sclerosis classic Hodgkin lymphoma | Nodular sclerosis classic Hodgkin lymphoma | Nodular sclerosis classic Hodgkin lymphoma |
| Lymphocyte-rich classic Hodgkin lymphoma | Lymphocyte-rich classic Hodgkin lymphoma | Lymphocyte-rich classic Hodgkin lymphoma |
| Mixed cellularity classic Hodgkin lymphoma | Mixed cellularity classic Hodgkin lymphoma | Mixed cellularity classic Hodgkin lymphoma |
| Lymphocyte-depleted classic Hodgkin lymphoma | Lymphocyte-depleted classic Hodgkin lymphoma | Lymphocyte-depleted classic Hodgkin lymphoma |

Updated since WHO 2017 (terminology/wording aligned between ICC 2022 and WHO 2022)

Updated since WHO 2017 (terminology/wording different between ICC 2022 and WHO 2022)

Minimal or no changes since WHO 2017

**Key**

**ICC 2022 changes within the HL section**

- Nodular lymphocyte predominant Hodgkin lymphoma replaced by nodular lymphocyte predominant B-cell lymphoma and listed as mature B-cell neoplasm entity

**WHO 2022 changes within the HL section**

- No entity changes since the WHO 2017 blue book

## 3.2 Nodular lymphocyte predominant Hodgkin lymphoma (NLPHL)

NLPHL is a germinal centre-derived B-cell neoplasm composed of scattered large neoplastic B cells with multilobated nuclei (LP cells), often within nodules dominated by mantle zone B cells and follicular dendritic cell meshworks. Variant histological growth patterns also occur in which small B cells are few and/or nodules are infrequent.

As mentioned above, the ICC 2022 classification has renamed NLPHL to nodular lymphocyte predominant B-cell lymphoma and listed it as a mature B-cell neoplasm. The WHO 2022 classification has retained the NLPHL category as per the WHO 2017 blue book but acknowledges that NLPHL “may be more accurately called nodular lymphocyte predominant B-cell lymphoma since the neoplastic cells have a functional B-cell programme, and therefore this term is now considered acceptable in preparation of future definitive adoption of the new nomenclature.”

## 3.3 Classic Hodgkin lymphoma (CHL)

CHL is a neoplasm derived from germinal centre B cells, characterised by a low fraction of tumour cells embedded in a reactive micro-environment rich in immune cells. The large neoplastic Hodgkin and Reed–Sternberg cells show a defective B-cell expression programme.

The WHO 2017 blue book recognised four subtypes: nodular sclerosis CHL, lymphocyte-rich CHL, mixed cellularity CHL and lymphocyte-depleted CHL. These subtypes remain unchanged in the ICC 2022 and WHO 2022 classifications. However, the WHO 2022 document does state that “the prognostic relevance of these subtypes has been lost due to modern treatment protocols.”

The CHL diagnostic criteria remain unchanged since 2017. The use of a standard immunohistochemical panel using CD30, CD15, IRF4/MUM1, PAX5, CD20, CD3 and LMP1 or EBER in situ hybridisation is recommended. Additional immunohistochemical or clonality studies may be warranted in the setting of atypical histological or clinical features.

# 4. Mature T- and NK-cell neoplasms

Mature T-cell neoplasms are a heterogeneous group of lymphoid neoplasms that arise from clonal proliferations of mature T lymphocytes in lymph nodes, bone marrow, blood or other tissues. Neoplasms that arise from natural killer (NK) cells are closely related and share some phenotypic features of mature T-cell neoplasms; therefore, these are categorised together.

## 4.1 Categorisation

Many of the mature T- and NK-cell neoplasm entities from the WHO 2017 blue book are included within the ICC 2022 and WHO 2022 classifications, but new types have also been identified and added to the 2022 classification documents. The differences between each document are listed in Table 7.

Table 7. Mature T-cell and NK-cell neoplasm entities

| **ICC 2022** | **WHO 2017** | **WHO 2022** |
| --- | --- | --- |
| **Mature T-cell and NK-cell leukaemias** | | |
| T-cell prolymphocytic leukaemia | T-cell prolymphocytic leukaemia | T-prolymphocytic leukaemia |
| T-cell large granular lymphocytic leukaemia | T-cell large granular lymphocytic leukaemia | T-large granular lymphocytic leukaemia |
| *Chronic lymphoproliferative disorder of NK cells* | *Chronic lymphoproliferative disorder of NK cells* | NK-large granular lymphocytic leukaemia |
| Aggressive NK-cell leukaemia | Aggressive NK-cell leukaemia | Aggressive NK-cell leukaemia |
| Adult T-cell leukaemia/lymphoma | Adult T-cell leukaemia/lymphoma | Adult T-cell leukaemia/lymphoma |
| Sézary syndrome | Sézary syndrome | Sézary syndrome |
| **EBV-positive T-cell/NK-cell lymphoproliferative disorders of childhood** | **EBV-positive T-cell and NK-cell lymphoproliferative diseases of childhood** | **EBV-positive T- and NK-cell lymphoid proliferations and lymphomas of childhood** |
| Systemic EBV-positive T-cell lymphoma of childhood | Systemic EBV-positive T-cell lymphoma of childhood | Systemic EBV-positive T-cell lymphoma of childhood |
| Chronic active EBV disease, systemic (T-and NK-cell phenotype) | Chronic active EBV infection of T-and NK-cell type, systemic form | Systemic chronic active EBV disease |
| Hydroa vacciniforme lymphoproliferative disorder | Hydroa vacciniforme-like lymphoproliferative disorder | Hydroa vacciniforme lymphoproliferative disorder |
| Classic |  | Classic |
| Systemic |  | Systemic |
| Severe mosquito bite allergy | Severe mosquito bite allergy | Severe mosquito bite allergy |
| **EBV-positive T-cell and NK-cell lymphomas** | | |
| Extranodal NK/T-cell lymphoma, nasal type | Extranodal NK/T-cell lymphoma, nasal type | Extranodal NK/T-cell lymphoma |
| *Primary nodal EBV-positive*  *T-cell/NK-cell lymphoma* |  | EBV-positive nodal T- and  NK-cell lymphoma |
| **T and NK-cell lymphomas and lymphoproliferative disorders of the gastrointestinal tract** | **Intestinal T-cell lymphomas** | **Intestinal T-cell and NK-cell lymphoid proliferations and lymphomas** |
| Enteropathy-associated T-cell lymphoma | Enteropathy-associated T-cell lymphoma | Enteropathy-associated T-cell lymphoma |
| Type II refractory coeliac disease |  |  |
| Monomorphic epitheliotropic intestinal T-cell lymphoma | Monomorphic epitheliotropic intestinal T-cell lymphoma | Monomorphic epitheliotropic intestinal T-cell lymphoma |
| Intestinal T-cell lymphoma, NOS | Intestinal T-cell lymphoma, NOS | Intestinal T-cell lymphoma, NOS |
| Indolent clonal T-cell lymphoproliferative disorder of the gastrointestinal tract | *Indolent T-cell lymphoproliferative disorder of the gastrointestinal tract* | Indolent T-cell lymphoma of the gastrointestinal tract |
| Indolent NK-cell lymphoproliferative disorder of the gastrointestinal tract |  | Indolent NK-cell lymphoproliferative disorder of the gastrointestinal tract |
| **Hepatosplenic T-cell lymphoma** | | |
| Hepatosplenic T-cell lymphoma | Hepatosplenic T-cell lymphoma | Hepatosplenic T-cell lymphoma |
| **Primary cutaneous T-cell lymphoid proliferations and lymphomas** | | |
| Subcutaneous panniculitis-like T-cell lymphoma | Subcutaneous panniculitis-like T-cell lymphoma | Subcutaneous panniculitis-like T-cell lymphoma |
| Mycosis fungoides | Mycosis fungoides | Mycosis fungoides |
| Primary cutaneous CD30+  T-cell lymphoproliferative disorders | Primary cutaneous CD30+  T-cell lymphoproliferative disorders | Primary cutaneous CD30+  T-cell lymphoproliferative disorder |
| Lymphomatoid papulosis | Lymphomatoid papulosis | Lymphomatoid papulosis |
| Primary cutaneous anaplastic large cell lymphoma | Primary cutaneous anaplastic large cell lymphoma | Primary cutaneous anaplastic large cell lymphoma |
|  | Primary cutaneous peripheral T-cell lymphomas, rare subtypes |  |
| Primary cutaneous gamma- delta T-cell lymphoma | Primary cutaneous gamma delta T-cell lymphoma | Primary cutaneous gamma/delta T-cell lymphoma |
| Primary cutaneous CD8+ aggressive epidermotropic cytotoxic T-cell lymphoma | *Primary cutaneous CD8+ aggressive epidermotropic cytotoxic T-cell lymphoma* | Primary cutaneous CD8+ aggressive epidermotropic cytotoxic T-cell lymphoma |
| Primary cutaneous acral CD8+ T-cell lymphoproliferative disorder | *Primary cutaneous acral CD8+ T-cell lymphoma* | Primary cutaneous acral CD8+ lymphoproliferative disorder |
| Primary cutaneous small/medium CD4+ T-cell lymphoproliferative disorder | *Primary cutaneous CD4+ small/medium T-cell lymphoproliferative disorder* | Primary cutaneous CD4+  small or medium T-cell lymphoproliferative disorder |
|  |  | Primary cutaneous peripheral T-cell lymphoma, NOS |
| **Follicular helper T-cell (TFH) lymphoma** | **Nodal lymphomas of T follicular helper (TFH) cell origin** | **Nodal T-follicular helper (TFH) cell lymphoma** |
| Angioimmunoblastic type (angioimmunoblastic T-cell lymphoma) | Angioimmunoblastic T-cell lymphoma | Angioimmunoblastic-type |
| Follicular type | Follicular T-cell lymphoma | Follicular type |
| NOS | Nodal peripheral T-cell lymphoma with TFH phenotype | NOS |
| **Other peripheral T-cell lymphomas** | | |
| Peripheral T-cell lymphoma, NOS | Peripheral T-cell lymphoma, NOS | Peripheral T-cell lymphoma, NOS |
| **Anaplastic large cell lymphoma** | | |
| Anaplastic large cell lymphoma, ALK-positive | Anaplastic large cell lymphoma, ALK-positive | ALK-positive anaplastic large cell lymphoma |
| Anaplastic large cell lymphoma, ALK-negative | Anaplastic large cell lymphoma, ALK-negative | ALK-negative anaplastic large cell lymphoma |
| Breast implant-associated anaplastic large cell lymphoma | *Breast implant-associated anaplastic large cell lymphoma* | Breast implant-associated anaplastic large cell lymphoma |

Italic font indicates provisional tumour entities.

Updated since WHO 2017 (terminology/wording aligned between ICC 2022 and WHO 2022)

Updated since WHO 2017 (terminology/wording different between ICC 2022 and WHO 2022)

Minimal or no changes since WHO 2017

**Key**

ALK, anaplastic lymphoma kinase; EBV, Epstein–Barr virus; NK, natural killer; NOS, not otherwise specified.

**ICC 2022 changes within the mature T- and NK-cell neoplasms section**

- Chronic active EBV disease replaces chronic active EBV infection and is restricted to patients who have the T-cell and NK-cell phenotype; B-cell patients are excluded
- Hydroa vacciniforme-like lymphoproliferative disorder is updated to hydroa vacciniforme lymphoproliferative disorder, and two distinct subtypes are specified – classic and systemic
- *Primary nodal EBV-positive T-cell/NK-cell lymphoma* added as a provisional entity
- Type II refractory coeliac disease added as a subentity/precursor of enteropathy-associated T-cell lymphoma
- *Indolent T-cell lymphoproliferative disorder of the gastrointestinal tract* renamed to indolent clonal T-cell lymphoproliferative disorder of the gastrointestinal tract and considered a definite entity
- Indolent NK-cell lymphoproliferative disorder of the gastrointestinal tract added as a new entity
- Primary cutaneous CD8+ aggressive epidermotropic cytotoxic T-cell lymphoma and primary cutaneous small/medium CD4+ T-cell lymphoproliferative disorder are considered definite entities
- *Primary cutaneous acral CD8+ T-cell lymphoma* renamed to primary cutaneous acral CD8+ T-cell lymphoproliferative disorder and considered a definite entity
- Follicular helper T-cell (TFH) lymphoma is considered a single entity that encompasses three subtypes:
  - Angioimmunoblastic-type (angioimmunoblastic T-cell lymphoma)
  - Follicular type
  - NOS (replaces nodal peripheral T-cell lymphoma with T-follicular helper phenotype)
- Breast implant-associated anaplastic large cell lymphoma is considered a definite entity

**WHO 2022 changes within the mature T- and NK-cell neoplasms section**

- The WHO 2022 document outlines nine families of mature T- and NK-cell neoplasm (in bold black text in Table 7):
  - Mature T-cell and NK-cell leukaemias
  - EBV-positive T- and NK-cell lymphoid proliferations and lymphomas of childhood
  - EBV-positive T-cell and NK-cell lymphomas
  - Intestinal T-cell and NK-cell lymphoid proliferations and lymphomas
  - Hepatosplenic T-cell lymphoma
  - Primary cutaneous T-cell lymphoid proliferations and lymphomas
  - Nodal T-follicular helper (TFH) cell lymphoma
  - Other peripheral T-cell lymphomas
  - Anaplastic large cell lymphoma
- T-cell large granular lymphocytic leukaemia renamed to T-large granular lymphocytic leukaemia
- NK-large granular lymphocytic leukaemia (definite entity) replaces c*hronic lymphoproliferative disorder of NK cells*
- Chronic active EBV infection of T- and NK-cell type: systemic form renamed to systemic chronic active EBV disease
- Hydroa vacciniforme-like lymphoproliferative disorder is updated to hydroa vacciniforme lymphoproliferative disorder, and two distinct subtypes are specified – classic and systemic
- ‘Nasal-type’ qualifier dropped from extranodal NK-/T-cell lymphoma
- EBV-positive nodal T-cell and NK-cell lymphoma added as a new entity
- Indolent T-cell lymphoproliferative disorder of the gastrointestinal tract is recognised as a definite entity and renamed to indolent T-cell lymphoma of the gastrointestinal tract
- Indolent NK-cell lymphoproliferative disorder of the gastrointestinal tract added as a new entity
- Primary cutaneous CD8+ aggressive epidermotropic cytotoxic T-cell lymphoma and primary cutaneous CD4+ small or medium T-cell lymphoproliferative disorder are considered definite entities
- *Primary cutaneous acral CD8+ T-cell lymphoma* renamed to primary cutaneous acral CD8+ lymphoproliferative disorder and considered a definite entity
- Primary cutaneous peripheral T-cell lymphoma, NOS added as a new entity
- Nodal T-follicular helper (TFH) cell lymphoma is considered a single entity that encompasses three subtypes:
  - Angioimmunoblastic type (replaces angioimmunoblastic T-cell lymphoma)
  - Follicular type (replaces follicular T-cell lymphoma)
  - NOS (replaces nodal peripheral T-cell lymphoma with TFH phenotype)
- Anaplastic large cell lymphoma, ALK-positive and anaplastic large cell lymphoma, ALK-negative reworded to ALK-positive anaplastic large cell lymphoma and ALK-negative anaplastic large cell lymphoma, respectively
- Breast implant-associated anaplastic large cell lymphoma is considered a definite entity

## 4.2 Mature T-cell and NK-cell leukaemias

The mature T-cell and NK-cell leukaemias family is comprised of six entities representative of T- and NK-cell proliferations that primarily present as leukaemic disease. Both the ICC and WHO 2022 classifications have provided updated information on mature T- and NK-cell neoplasms since the WHO 2017 blue book was published.

### 4.2.1 T-cell prolymphocytic/T-prolymphocytic leukaemia (T-PLL)

T-PLL is a rare, aggressive T-cell leukaemia characterised by the proliferation of small to medium-sized prolymphocytes with a mature post-thymic T-cell phenotype, involving the peripheral blood, bone marrow, lymph nodes, liver, spleen and skin.

The ICC 2022 and WHO 2022 classifications have provided updated information on the diagnosis of T-PLL since the WHO 2017 blue book was published, which can be found in the de Leval L, et al. 2022 special report and the online WHO beta v2 version, respectively.

### 4.2.2 T-cell large/T-large granular lymphocytic leukaemia (T-LGLL)

T-LGLL is a neoplastic proliferation of cytotoxic large granular T cells with persistent peripheral lymphocytosis.

The ICC 2022 and WHO 2022 classifications have provided updated information on the diagnosis and prognosis of T-LGLL since the WHO 2017 blue book was published, which can be found in the de Leval L, et al. 2022 special report and the online WHO beta v2 version, respectively.

### 4.2.3 Chronic lymphoproliferative disorder of NK cells / NK-large granular lymphocytic leukaemia

Chronic lymphoproliferative disorders of NK cells (CLPD-NKs) are rare and heterogeneous. They are characterised by a persistent increase in the peripheral blood NK-cell count (usually to >2 x 10^9^/L) without a clearly identified cause and a chronic indolent clinical course.

In both the WHO 2017 blue book and the ICC 2022 classification, CLPD-NKs are included as a provisional entity. In the WHO 2022 classification, CLPD-NK has been renamed to NK-large granular lymphocytic leukaemia, given recent evidence that this is a monoclonal or oligoclonal expansion of NK cells that has many similarities with T-LGLL.

### 4.2.4 Aggressive NK-cell leukaemia

Aggressive NK-cell leukaemia is a systemic malignant proliferation of NK cells frequently associated with EBV with an acute presentation and an aggressive clinical course.

The ICC 2022 and WHO 2022 classifications have provided updated information on the diagnosis of aggressive NK-cell leukaemia since the WHO 2017 blue book was published, which can be found in the de Leval L, et al. 2022 special report and the online WHO beta v2 version, respectively.

### 4.2.5 Adult T-cell leukaemia/lymphoma (ATLL)

ATLL is a mature T-cell neoplasm most often composed of highly pleomorphic lymphoid cells and associated with human retrovirus HTLV-1. Most ATLL patients present with widespread lymph node involvement as well as involvement of peripheral blood.

The WHO 2017 blue book recognised four subtypes of ATLL: acute, lymphoma, chronic and smouldering. There have been no updates to ATLL in the ICC 2022 classification. The WHO 2022 classification retains these four subtypes; however, chronic type ATLL is further divided into unfavourable and favourable subtypes based on the presence of lactate dehydrogenase (LDH) or blood urea nitrogen (BUN) levels above the normal upper limits or an albumin level below the normal lower limit. Acute, lymphoma and unfavourable chronic subtypes of ATLL, which have a similar prognosis, are grouped as aggressive ATLL, while favourable chronic and smouldering ATLLs are grouped as indolent ATLL.

The WHO 2022 document states that “genetic analyses of ATLL have revealed novel events that impact disease pathogenesis.” In addition, it is noted that aggressive subtypes show more genetic alterations, whereas *STAT3* mutations are more common in indolent subtypes. In the WHO 2022 classification, the subtypes are further stratified into subsets with different prognosis by genetic alterations (Table 8).

Table 8. Clinical prognostic indices and genetic prognostic markers for ATLL in the WHO 2022 classification

|  | **Acute subtype** | **Lymphoma subtype** | **Unfavourable chronic subtype** | **Favourable chronic subtype** | **Smouldering subtype** |
| --- | --- | --- | --- | --- | --- |
| **Clinical prognostic indices** | | | | | |
| ATL-PI | Stage, PS, age, albumin,  sIL2-R | |  | | |
| iATL-PI |  | | sIL-2R | | |
| JCOG-PI | Corrected Ca, PS | | |  | |
| Modified ATL-PI | Clinical subtype, PS, corrected Ca, sIL-2R | |  | | |
| New prognostic score for ATL from North America | PS, stage, calcium, age | | | | |
| **Genetic prognostic markers** | | | | | |
| Kataoka et al. | *PD-L1* amplification,  *PRKCB* mutations | | *IRF4* mutations, *PD-L1* amplification,  *CDKN2A* deletions | | |

ATL-PI, adult T-cell leukaemia-prognostic index; ECOG, Eastern Cooperative [Oncology](https://www.sciencedirect.com/topics/medicine-and-dentistry/oncology) Group; iATL-PI, indolent adult T-cell leukaemia-prognostic index; JCOG-PI, Japan Clinical Oncology Group-prognostic index; PS, ECOG performance status; sIL-2R, soluble interleukin-2 receptor.

### 4.2.6 Sézary syndrome (SS)

SS is a neoplasm of T lymphocytes defined by the triad of erythroderma, generalised lymphadenopathy and the presence of clonally related neoplastic T cells with cerebriform nuclei (Sézary cells) in skin, lymph nodes and peripheral blood. SS and mycosis fungoides are closely related neoplasms but are considered separate entities on the basis of differences in clinical behaviour and cell of origin.

There have been no major updates to SS since 2017.

## 4.3 EBV-positive T-cell and NK-cell lymphoproliferative diseases / lymphoid proliferations and lymphomas of childhood

EBV-positive T-cell and NK-cell lymphoproliferative diseases of childhood (or EBV-positive T-cell and NK-cell lymphoid proliferations and lymphomas of childhood in the WHO 2022 classification) are a group of uncommon disorders characterised by EBV infection of T- and NK-cells. They occur with increased frequency in Asian and native American ethnic groups. This group of disorders is comprised of four entities. The ICC 2022 and WHO 2022 classifications provide updated information on each entity except systemic EBV-positive T-cell lymphoma of childhood, which remains unchanged since 2017.

### 4.3.1 Chronic active EBV disease

The WHO 2017 blue book included the entity ‘chronic active EBV infection of T-cell or NK-cell type, systemic form’, which is defined as a systemic EBV positive polyclonal, oligoclonal or (often) monoclonal lymphoproliferative disorder characterised by fever, persistent hepatitis, hepatosplenomegaly and lymphadenopathy. The ICC 2022 and WHO 2022 classifications have renamed this entity to chronic active EBV disease, systemic (T- and NK-cell phenotype) and systemic chronic active EBV disease, respectively. The term ‘disease’ is preferred over ‘infection’ because most adults are chronically infected with EBV.

There have been no other major updates to chronic active EBV disease in the ICC 2022 and WHO 2022 classifications. However, both documents clarify that this entity should include only T- or NK-cell disease. B-cell disorders are excluded.

### 4.3.2 Hydroa vacciniforme lymphoproliferative disorder (HV-LPD)

HV-LPD is a cutaneous form of chronic active EBV disease. Patients present with skin lesions on sun-exposed areas with EBV-infected T or NK cells and very high levels of EBV DNA in blood. In the WHO 2017 blue book, this entity was referred to as ‘hydroa vacciniforme-like lymphoproliferative disorder’; however, it is now known that all HV lesions have EBV and therefore the updated term ‘HV-LPD’ is used in both the ICC 2022 and WHO 2022 classifications.

The ICC 2022 and WHO 2022 classifications also recognise two forms of HV-LPD – the classic form (which is indolent and only involves the skin) and the systemic form (which is severe and includes fever, lymphadenopathy and often liver involvement). The WHO 2022 document emphasises the need to distinguish the systemic form of HV-LPD from chronic active EBV disease (without HV-LPD).

### 4.3.3 Severe mosquito bite allergy

The WHO 2017 blue book defined severe mosquito bite allergy as an EBV-positive NK-cell lymphoproliferative disorder characterised by high fever and severe skin manifestations, including bullae, ulcers, necrosis and scarring after mosquito bites.

There have been no updates to this entity in the ICC 2022 classification. The WHO 2022 classification recognises that a small subset of severe mosquito bite allergies are derived from T cells.

## 4.4 EBV-positive NK-/T-cell lymphomas

The ICC 2022 and WHO 2022 classifications have provided updated information on EBV-positive NK-/T-cell lymphomas since the WHO 2017 blue book was published.

### 4.4.1 Extranodal NK-/T-cell lymphoma (nasal type)

Extranodal NK-/T-cell lymphoma, nasal type is a predominantly extranodal lymphoma of NK-cell or T-cell lineage, characterised by vascular infiltration and destruction by the tumour cells, prominent necrosis, cytotoxic phenotype and association with EBV.

There have been no major updates to this entity in the ICC 2022 classification. In the WHO 2022 classification, the ‘nasal type’ qualifier has been dropped in accordance with the recognised presentation of this disease at various extranodal sites. The ICC 2022 classification retains the ‘nasal type’ qualifier to emphasise that in other sites, the morphology is similar to those cases presented in the nasal region.

### 4.4.2 Primary nodal EBV-positive T-cell/NK-cell lymphoma / EBV-positive nodal T- and NK-cell lymphoma

This is a rare EBV-positive lymphoma of cytotoxic T- or NK-cell lineage that occurs mostly in East Asia in elderly and/or immunodeficient patients. Patients typically present with lymphadenopathy with or without extranodal involvement, advanced-stage disease and B symptoms; they have a dismal prognosis.

In the WHO 2017 blue book, primary nodal EBV-positive T- or NK-cell lymphoma was introduced as a variant of peripheral T-cell lymphoma, NOS. In the ICC 2022 classification, *primary nodal EBV-positive T-cell/NK-cell lymphoma* is considered a provisional entity. In the WHO 2022 classification, EBV-positive nodal T- and NK-cell lymphoma is recognised as a distinct entity.

## 4.5 Intestinal T-cell and NK-cell lymphoid proliferations and lymphomas / T- and NK-cell lymphomas and lymphoproliferative disorders of the gastrointestinal tract

Some entities within this family of disorders have been updated since the WHO 2017 blue book, and some new entities have been added (as described in section 4.1).

Further information on the new/updated entities can be found in sections 4.5.1 to 4.5.3 below. The remaining entities have stayed largely the same since 2017.

### 4.5.1 Type II refractory coeliac disease

The ICC 2022 classification has added this entity as a precursor of enteropathy-associated T-cell lymphoma (EATL). EATL and type II refractory coeliac disease have frequent gain-of-function mutations in *STAT3* and *JAK1*.

### 4.5.2 Indolent T-cell lymphoproliferative disorder of the gastrointestinal tract

The WHO 2017 blue book included *indolent T-cell lymphoproliferative disorder of the gastrointestinal tract (ITP)* as a provisional entity. In this disorder, the lymphoid cells mainly infiltrate the lamina propria but usually do not invade the epithelium. The clinical course is indolent, but most patients do not respond to conventional chemotherapy. A subset of cases progress to a higher-grade T-cell lymphoma with spread beyond the gastrointestinal tract.

In the ICC 2022 classification, ITP is considered a definite entity and renamed ‘indolent clonal T-cell lymphoproliferative disorder of the gastrointestinal tract’, in acknowledgement of its monoclonal nature. In the WHO 2022 classification, this entity is renamed ‘indolent T-cell lymphoma of the gastrointestinal tract’ due to the significant morbidity related to the tumour and the ability of the disease to disseminate. The qualifier ‘indolent’ remains to indicate its protracted clinical course.

### 4.5.3 Indolent NK-cell lymphoproliferative disorder of the gastrointestinal tract

Indolent NK-cell lymphoproliferative disorder of the gastrointestinal tract, which was previously referred to as ‘lymphomatoid gastropathy or NK-cell enteropathy’ and previously thought to be a reactive process, is included as a new distinct entity in the ICC 2022 and WHO 2022 classifications because of recent findings supporting its neoplastic nature.

## 4.6 Hepatosplenic T-cell lymphoma

Hepatosplenic T-cell lymphoma is an aggressive mature T-cell lymphoma characterised by a proliferation of cytotoxic T cells involving the spleen, liver and bone marrow.

There have been no major updates to this entity since 2017.

## 4.7 Primary cutaneous T-cell lymphoproliferative disorders and lymphomas

The primary cutaneous T-cell lymphoid proliferations and lymphomas family comprises a group of entities with diverse clinical and genetic features, different phenotypes and varying histology. Some entities of primary cutaneous T-cell lymphoid proliferations and lymphomas have been updated since the WHO 2017 blue book, and one new entity has been added (as described in section 4.1).

Further information on the new/updated entities can be found in sections 4.7.1 and 4.7.2 below. The remaining entities have stayed largely the same since 2017.

### 4.7.1 Primary cutaneous peripheral T-cell lymphomas, rare subtypes

In the WHO 2017 blue book, primary cutaneous gamma/delta T-cell lymphoma, CD8+ aggressive epidermotropic cytotoxic T-cell lymphoma, acral CD8+ T-cell lymphoma and CD4+ small/medium T-cell lymphoproliferative disorder were grouped together under the term ‘cutaneous peripheral T-cell lymphoma, rare subtypes’. In the ICC 2022 and WHO 2022 classifications, these are now listed as separate entities acknowledging their specific clinicopathological and genetic characteristics.

In addition, primary cutaneous acral CD8+ T-cell lymphoma is now classified as a lymphoproliferative disorder in both the ICC 2022 and WHO 2022 documents due to its indolent course and general need for only local-type therapies or even just observation.

### 4.7.2 Primary cutaneous peripheral T-cell lymphoma, NOS

The WHO 2022 classification has added this as a new entity to be used for the very rare cases that do not fit into any of the other known primary cutaneous T-cell lymphoproliferative disorder and lymphoma entities.

## 4.8 Peripheral T-cell lymphoma, not otherwise specified (PTCL, NOS)

PTCL, NOS is a heterogeneous entity of nodal and extranodal mature T-cell lymphomas that cannot be assigned to a specific PTCL entity. Nodal TFH-cell lymphomas and EBV-positive nodal T- and NK-cell lymphomas are excluded from this entity.

There have been no major updates to this entity since 2017.

## 4.9 Nodal lymphomas of T-follicular helper (TFH) cell origin

In the WHO 2017 blue book, nodal lymphomas of T-follicular helper (TFH) cell origin represent a group of mature T-cell neoplasms with phenotypic features and gene expression signature of TFH cells, a subset of effector T-helper cells residing predominantly in secondary lymphoid follicles. In the ICC 2022 classification this group has been renamed ‘follicular helper T-cell lymphoma (TFH lymphoma)’. In the WHO 2022 classification the term ‘nodal T-follicular helper (TFH) cell lymphoma’ is used.

The WHO 2017 blue book defined three distinct entities of nodal lymphoma of TFH-cell origin: angioimmunoblastic T-cell lymphoma, follicular T-cell lymphoma and nodal peripheral T-cell lymphoma with TFH phenotype. In contrast with this, in the ICC 2022 and WHO 2022 classifications, TFH lymphoma or nodal-TFH cell lymphoma are considered single entities that encompass three subtypes: angioimmunoblastic-type, follicular-type and NOS.

## 4.10 Anaplastic large cell lymphoma (ALCL)

ALCLs are a group of mature T-cell lymphomas that share common cytomorphological and immunophenotypic features but are clinically, pathologically and genetically heterogeneous. The ICC 2022 and WHO 2022 classifications have provided updated information on ALCLs since the WHO 2017 blue book was published.

### 4.10.1 ALK-positive ALCL

ALK-positive ALCL is a CD30+ mature T-cell lymphoma with aberrant expression of the anaplastic lymphoma kinase (ALK) protein secondary to rearrangements of the*ALK* gene.

There have been no major updates to this entity since 2017.

### 4.10.2 ALK-negative ALCL

ALK-negative ALCL is a mature T-cell lymphoma with uniform strong expression of CD30, without ALK expression or *ALK* rearrangement.

There have been no major updates to the diagnostic criteria for ALK-negative ALCL since 2017. However, recent genomic analyses have led to recognition of different genetic subtypes in the ICC 2022 and WHO 2022 classifications:

Genetic subtypes of ALK-negative ALCL defined in the ICC 2022 classification

- ALK-negative ALCL with *DUSP22*-rearrangement

(ALK-negative ALCL with *TP63* rearrangements are associated with worse outcome; ALK-negative ALCL with *JAK2* rearrangements or coexisting *TP63* and *DUSP22* rearrangements require further study).

Genetic subtypes of ALK-negative ALCL defined in the WHO 2022 classification

- ALK-negative ALCL with *TP63* rearrangements
- ALK-negative ALCL with loss of *TP53* and/or overexpression of IL-2Rα
- ALK-negative ALCL with *DUSP22*-rearrangement

### 4.10.3 Breast implant-associated anaplastic large cell lymphoma

Breast implant-associated ALCL is a mature CD30+ T-cell lymphoma which arises in response to textured-surface breast implants and is associated with distinct biologic behaviour and generally excellent outcome.

In the WHO 2017 blue book, breast implant-associated ALCL was considered a provisional entity. In the ICC 2022 and WHO 2022 classifications, it has been upgraded to a definite entity. There have been no other major updates to this entity since 2017. However, both 2022 classifications recommend the use of tumour-node-metastasis staging criteria to facilitate clinical management.

# 5. Immunodeficiency-associated lymphoproliferative disorders

Immune deficiency/dysregulation-associated lymphoproliferative disorders are a heterogeneous group of lymphoid disorders that occur in people with primary (inborn) or secondary (acquired) immunodeficiency.

## 5.1 Categorisation

The WHO 2017 blue book included four main categories of immunodeficiency-associated lymphoproliferative disorders. The updated ICC 2022 document includes two of the main categories from the WHO 2017 blue book. In contrast, the WHO 2022 document introduced major changes to the reporting of immunodeficiency-associated lymphoproliferative disorders with new designations included, to align with the International Union of Immunological Societies (IUIS) classification. The differences between each document are listed in Table 9.

In addition, the WHO 2022 document has introduced a new family, ‘lymphoid proliferations and lymphomas associated with immune deficiency and dysregulation’, to replace immunodeficiency-associated lymphoproliferative disorders, and included this group of disorders under mature B-cell neoplasms. In the WHO 2017 blue book and the ICC 2022 classification, immunodeficiency-associated lymphoproliferative disorders are included as separate sections to mature B-cell neoplasms.

Table 9. Immunodeficiency-associated lymphoproliferative disorder entities

| **ICC 2022** | **WHO 2017** | **WHO 2022*** |
| --- | --- | --- |
|  |  | Inborn error of immunity-associated lymphoid proliferations  and lymphomas |
|  |  | Lymphomas arising in immune deficiency / dysregulation |
| Post-transplant lymphoproliferative disorders (PTLD) | Post-transplant lymphoproliferative disorders (PTLD) |  |
| Monomorphic PTLD  (B- and T-/NK-cell types)† | Monomorphic PTLD  (B- and T/NK-cell types) |  |
| Classic Hodgkin lymphoma PTLD† | Classic Hodgkin lymphoma PTLD |  |
| Non-destructive PTLD   - Plasmacytic hyperplasia PTLD - Infectious mononucleosis PTLD - Florid follicular hyperplasia | Non-destructive PTLD   - Plasmacytic hyperplasia PTLD - Infectious mononucleosis PTLD - Florid follicular hyperplasia | Hyperplasias arising in immune deficiency/ dysregulation |
| Polymorphic PTLD | Polymorphic PTLD | Polymorphic lymphoproliferative disorders arising in immune  deficiency/dysregulation‡ |
| Other iatrogenic immunodeficiency-associated lymphoproliferative disorders | Other iatrogenic immunodeficiency-associated lymphoproliferative disorders |  |
| Updated since WHO 2017 (terminology/wording aligned between ICC 2022 and WHO 2022)  Updated since WHO 2017 (terminology/wording different between ICC 2022 and WHO 2022)  Minimal or no changes since WHO 2017  **Key** |  | EBV-positive mucocutaneous ulcer |

* Note: The classification concept in the WHO 2022 document is different from the WHO 2017 blue book and the ICC 2022 classification and, therefore, a direct translation of individual entities is not possible.

† These lesions are classified according to the lymphoma to which they correspond.

‡ Lymphoproliferations/lymphomas post transplant or in HIV can manifest as hyperplasias, polymorphic lymphoproliferative disorders and/or lymphomas.

**ICC 2022 changes within the immunodeficiency-associated lymphoproliferative disorders section**

- No major changes since the WHO 2017 blue book

**WHO 2022 changes within the immunodeficiency-associated lymphoproliferative disorders section**

- Hyperplasias arising in immune deficiency/dysregulation added as a new name to encompass non-destructive lymphoproliferative disorders
- Polymorphic lymphoproliferative disorders arising in immune deficiency/dysregulation added as a new entity to encompass polymorphic lymphoproliferative disorders
- EBV-positive mucocutaneous ulcer included in this family instead of under mature B-cell neoplasms
- Lymphomas arising in immune deficiency/dysregulation added as a new entity to encompass overt lymphomas, such as DLBCL, classic Hodgkin lymphoma or others
- Inborn error of immunity-associated lymphoid proliferations and lymphomas added as a new entity to replace lymphoproliferative diseases associated with primary immune disorders

## 5.2 ICC 2022 classification updates

Immunodeficiency-associated lymphoproliferative disorders were discussed by the CAC, and it was decided that the classification and entities from the WHO 2017 blue book should be maintained in the ICC 2022 classification. It was felt that the nomenclature proposed by the 2015 Workshop of the Society for Hematopathology/European Association for Haematopathology did not sufficiently define and separate distinct clinical entities.

## 5.3 WHO 2022 framework and nomenclature changes

In the WHO 2017 blue book, immunodeficiency-associated lymphoproliferative disorders were grouped according to the disease background in which they arose and were discussed in separate chapters: primary immunodeficiencies, HIV infection, post transplantation and other iatrogenic immunodeficiencies.

Inspired by the 2015 Workshop of the Society for Hematopathology/European Association for Haematopathology, the WHO 2022 classification introduces a new overarching framework and a standardised nomenclature to cover the different settings of immune dysfunction.

Further details on the new framework and nomenclature changes are provided in the WHO 2022 classification document.

# 6. Histiocytic and dendritic cell neoplasms

Histiocytic neoplasms are derived from mononuclear phagocytes (macrophages and dendritic cells) or histiocytes. Dendritic cell tumours are related to several lineages of accessory antigen-presenting cells (dendritic cells) that have a role in phagocytosis, processing and presentation of antigen to lymphoid cells.

## 6.1 Categorisation

Most histiocytic and dendritic neoplasm entities from the WHO 2017 blue book are included within both the ICC 2022 and WHO 2022 classifications, but new types have also been identified and added to the 2022 classification documents. The differences between each document are listed in Table 10.

In addition, the WHO 2022 classification places histiocytic/dendritic cell neoplasms after myeloid neoplasms (in the Khoury J, et al. 2022 Leukaemia companion paper), in recognition of their derivation from common myeloid progenitors that give rise to cells of the monocytic/histiocytic/dendritic lineages. Neoplasms that arise from lymphoid stromal cells are classified as ‘stroma-derived neoplasms of lymphoid tissues’ and are now included within the Alaggio R, et al. 2022 companion manuscript with the other lymphoid neoplasms described above.

Table 10. Histiocytic and dendritic cell neoplasm entities

| **ICC 2022** | **WHO 2017** | **WHO 2022** |
| --- | --- | --- |
|  |  | **Plasmacytoid dendritic cell neoplasms*** |
|  |  | Mature plasmacytoid dendritic cell proliferation associated with myeloid neoplasm* |
| Blastic plasmacytoid dendritic cell neoplasm† | Blastic plasmacytoid dendritic cell neoplasm‡ | Blastic plasmacytoid dendritic cell neoplasm* |
|  |  | **Langerhans cell and other dendritic cell neoplasms*** |
| Tumours derived from Langerhans cells | Tumours derived from Langerhans cells | Langerhans cell neoplasms* |
| Langerhans cell histiocytosis | Langerhans cell histiocytosis | Langerhans cell histiocytosis* |
| Langerhans cell sarcoma | Langerhans cell sarcoma | Langerhans cell sarcoma* |
|  |  | Other dendritic cell neoplasms* |
| Indeterminate dendritic cell histiocytosis | Indeterminate dendritic cell tumour | Indeterminate dendritic cell tumour* |
| Interdigitating dendritic cell sarcoma | Interdigitating dendritic cell sarcoma | Interdigitating dendritic cell sarcoma* |
| **Mesenchymal dendritic cell neoplasms^§^** | | |
| Follicular dendritic cell sarcoma | Follicular dendritic cell sarcoma | Follicular dendritic cell sarcoma**^§^** |
| EBV-positive inflammatory follicular dendritic cell/fibroblastic reticular cell tumour | Inflammatory pseudotumour-like follicular/fibroblastic dendritic cell sarcoma | EBV-positive inflammatory follicular dendritic cell sarcoma**^§^** |
| Fibroblastic reticular cell sarcoma | Fibroblastic reticular cell tumour | Fibroblastic reticular cell tumour**^§^** |
| **Histiocytic neoplasms*** | | |
| ALK-positive histiocytosis |  | ALK-positive histiocytosis* |
| Disseminated juvenile xanthogranuloma | Disseminated juvenile xanthogranuloma | Juvenile xanthogranuloma* |
| Erdheim–Chester disease | Erdheim–Chester disease | Erdheim–Chester disease* |
| Rosai–Dorfman–Destombes disease |  | Rosai–Dorfman disease* |
| Histiocytic sarcoma | Histiocytic sarcoma | Histiocytic sarcoma* |
|  |  | **Myofibroblastic tumour^§^** |
|  |  | Intranodal palisaded myofibroblastoma**^§^** |
|  |  | **Spleen-specific vascular-stromal tumours^§^** |
|  |  | Littoral cell angioma**^§^** |
|  |  | Splenic hamartoma**^§^** |
|  |  | Sclerosing angiomatoid nodular transformation of spleen**^§^** |

Updated since WHO 2017 (terminology/wording aligned between ICC 2022 and WHO 2022)

Updated since WHO 2017 (terminology/wording different between ICC 2022 and WHO 2022)

Minimal or no changes since WHO 2017

**Key**

ALK, anaplastic lymphoma kinase; EBV, Epstein–Barr virus.

* Included within the 5th edition of the World Health Organization Classification of Haematolymphoid Tumours: Myeloid and Histiocytic/Dendritic Neoplasms (Khoury JD, et al., 2022).

† Included within the International Consensus Classification of Myeloid Neoplasms and Acute Leukemias: integrating morphologic, clinical, and genomic data (Arber DA, et al., 2022) and not under histiocytic and dendritic cell neoplasms.

† Included in a separate section of the WHO 2017 blue book and not under histiocytic and dendritic cell neoplasms.

**^§^** Classified as a stroma-derived neoplasm of lymphoid tissues and included within the 5th edition of the World Health Organization Classification of Haematolymphoid Tumours: Lymphoid Neoplasms (Alaggio R, et al., 2022).

**ICC 2022 changes within the histiocytic and dendritic cell neoplasms section**

- Indeterminate dendritic cell tumour replaced with indeterminate dendritic cell histiocytosis
- Inflammatory pseudotumour-like follicular/fibroblastic dendritic cell sarcoma renamed to EBV-positive inflammatory follicular dendritic cell/fibroblastic reticular cell tumour
- Fibroblastic reticular cell tumour replaced with fibroblastic reticular cell sarcoma
- ALK-positive histiocytosis and Rosai–Dorfman–Destombes disease added as new entities

**WHO 2022 changes within the histiocytic and dendritic cell neoplasms and stroma-derived neoplasms sections**

- These entities are now listed in two separate sections:
  - Histiocytic/dendritic cell neoplasms, comprised of three families:
    - Plasmacytoid dendritic cell neoplasms
    - Langerhans cell and other dendritic cell neoplasms
    - Histiocyte/macrophage neoplasms
  - Stroma-derived neoplasms of lymphoid tissues, comprised of three families:
    - Mesenchymal dendritic cell neoplasms
    - Myofibroblastic tumours
    - Spleen-specific vascular-stromal tumours
- Mature plasmacytoid dendritic cell proliferation associated with myeloid neoplasm and blastic plasmacytoid dendritic cell neoplasm added as new entities under plasmacytoid dendritic cell neoplasms
- Inflammatory pseudotumour-like follicular/fibroblastic dendritic cell sarcoma renamed to EBV-positive inflammatory follicular dendritic cell sarcoma
- Two new entities of histiocytic neoplasm added – ALK-positive histiocytosis and Rosai–Dorfman disease
- Disseminated juvenile xanthogranuloma renamed to juvenile xanthogranuloma
- Intranodal palisaded myofibroblastoma added as a new entity of myofibroblastic tumour
- Three entities of splenic vascular-stromal tumours (littoral cell angioma, splenic hamartoma and sclerosing angiomatoid nodular transformation of spleen) added to spleen-specific vascular-stromal tumours family

## 6.2 Inflammatory pseudotumour-like follicular/fibroblastic dendritic cell sarcoma

In the WHO 2017 blue book, inflammatory pseudotumour-like follicular/fibroblastic dendritic cell sarcoma was included as a variant of follicular dendritic cell sarcoma. This entity is defined as an indolent proliferation of stromal cells of mesenchymal origin not derived from haematopoietic stem cells. Neoplastic cells are EBV positive and are associated with a rich inflammatory background. Spleen and liver are the most common sites, but it can also arise in other extranodal locations.

Given its distinctive clinicopathologic features, the ICC 2022 and WHO 2022 classifications recognise this as a distinct and separate entity to follicular dendritic cell sarcoma. In the ICC 2022 classification, it is renamed ‘EBV-positive inflammatory follicular dendritic cell/fibroblastic reticular cell tumour’, whereas the WHO 2022 classification uses the term ‘EBV-positive inflammatory follicular dendritic cell sarcoma.’

## 6.3 ALK-positive histiocytosis

The ICC 2022 and WHO classifications have introduced ALK-positive histiocytosis as a new entity. It is a histiocytic neoplasm lacking high-grade cytologic atypia and characterised by ALK immunoreactivity usually due to *ALK* gene rearrangement.

## 6.4 Rosai–Dorfman (–Destombes) disease

The ICC 2022 and WHO 2022 classifications have introduced this as a new entity. Rosai–Dorfman disease (WHO 2022) or Rosai–Dorfman–Destombes disease (ICC 2022) is a histiocytosis characterised by nodal or extranodal accumulation of large, S100-positive histiocytes/macrophages that commonly exhibit emperipolesis.

## 6.5 Other histiocytic and dendritic cell neoplasms

Further details on other histiocytic and dendritic cell neoplasms are provided in the WHO 2022 and ICC 2022 classifications.

# 7. Conclusion

In summary, this paper has compared the ICC and WHO 2022 classifications for mature lymphoid neoplasms and examined the updates to different entities that have been introduced since the WHO 2017 blue book was published. The discussion was structured around five key subsections, namely mature B-cell neoplasms, Hodgkin lymphomas, mature T- and NK-cell neoplasms, immunodeficiency-associated lymphoproliferative disorders, and histiocytic and dendritic cell neoplasms.

The following references were used in the development of this paper and can provide further information, as required:

- Swerdlow SH, Campo E, Harris NL, et al., editors. WHO Classification of Tumours of Haematopoietic and Lymphoid Tissues. Revised 4th ed. IARC; 2017
- Campo E, Jaffe ES, Cook JR, et al. The International Consensus Classification of Mature Lymphoid Neoplasms: A report from the Clinical Advisory Committee. Blood. 2022;140(11):1229–53
- Arber DA, Orazi A, Hasserjian RP, et al. International Consensus Classification of Myeloid Neoplasms and Acute Leukemias: integrating morphologic, clinical, and genomic data. Blood. 2022;140(11):1220–28
- Alaggio R, Amador C, Anagnostopoulos I, et al. The 5th edition of the World Health Organization Classification of Haematolymphoid Tumours: Lymphoid Neoplasms. Leukemia 2022;36:1720–48
- Khoury JD, Solary E, Abla O, et al. The 5th edition of the World Health Organization Classification of Haematolymphoid Tumours: Myeloid and Histiocytic/Dendritic Neoplasms. Leukemia 2022;36:1703–19
- WHO Classification of Tumours Editorial Board. Haematolymphoid Tumours. 5th ed. [Internet; beta version ahead of print]. Available at: https://tumourclassification.iarc.who.int/chapters/63 [cited 17 October 2023]
- de Leval L, Alizadeh AA, Bergsagel PL, et al. Genomic profiling for clinical decision making in lymphoid neoplasms. Blood 2022;140:2193–227
- Laurent C, Cook JR, Yoshino T, et al. Follicular lymphoma and marginal zone lymphoma: how many diseases? Virchows Arch 2023;482:149–62
- Fend F, Dogan A, Cook JR. Plasma cell neoplasms and related entities – evolution in diagnosis and classification. Virchows Arch 2023;482:163–77

# 8. Acknowledgements

The authors would like to thank Elias Campo, Elaine Jaffe, James Cook, Steven Swerdlow, Leticia Quintanilla-Martinez (members of the Pathology Steering committee of the CAC) and German Ott for providing feedback and comments during the preparation of the manuscript.  These individuals do not necessarily endorse all the conclusions and opinions that are presented herein.

Medical writing support was provided by Elements Communications Ltd., UK and funded by the European Hematology Association.
