# Supplementary material for: Precision hematology: Navigating the evolution of diagnostic classifications in the era of globalized medicine
Source: Hemasphere. 2024 Apr 4;8(4):e65. doi: 10.1002/hem3.65 (PMC10993146; doi:10.1002/hem3.65)
Supplement: Supplementary file 2 — Supplement 2: Updates to the classification of myeloid neoplasms and acute leukaemias: an overview of changes since the WHO 2017 Blue Book. [file HEM3-8-e65-s002.docx]

**Updates to the classification of myeloid neoplasms and
acute leukaemias: an overview of changes since the**

**WHO 2017 Blue Book**

Table of Contents

[1. Introduction 3](#_Toc148891536)

[2. Myeloproliferative neoplasms (MPN) 5](#_Toc148891537)

[2.1 Categorisation 5](#_Toc148891538)

[2.2 Diagnostic criteria 6](#_Toc148891539)

[⮚ 2.2.1 Chronic myeloid leukaemia (CML) 6](#_Toc148891540)

[⮚ 2.2.2 Chronic neutrophilic leukaemia (CNL) 8](#_Toc148891541)

[⮚ 2.2.3 Polycythaemia vera (PV) 11](#_Toc148891542)

[⮚ 2.2.4 Primary myelofibrosis (PMF) 13](#_Toc148891543)

[⮚ 2.2.5 Essential thrombocythaemia (ET) 14](#_Toc148891544)

[⮚ 2.2.6 Chronic eosinophilic leukaemia, not otherwise specified (CEL, NOS) 16](#_Toc148891545)

[⮚ 2.2.7 Myeloproliferative neoplasm, unclassifiable (MPN-U) 19](#_Toc148891546)

[3. Mastocytosis 21](#_Toc148891547)

[3.1 Categorisation 21](#_Toc148891548)

[3.2 Diagnostic criteria 22](#_Toc148891549)

[⮚ 3.2.1 Cutaneous mastocytosis 22](#_Toc148891550)

[⮚ 3.2.2 Systemic mastocytosis (SM) 23](#_Toc148891551)

[3.4 B and C findings 25](#_Toc148891552)

[4. Myeloid/lymphoid neoplasms with eosinophilia and tyrosine kinase gene fusions 26](#_Toc148891553)

[4.1 Categorisation 26](#_Toc148891554)

[5. Myelodysplastic/myeloproliferative neoplasms (MDS/MPN) 27](#_Toc148891555)

[5.1 Categorisation 27](#_Toc148891556)

[5.2 Diagnostic criteria 29](#_Toc148891557)

[⮚ 5.2.1 Chronic myelomonocytic leukaemia (CMML) 29](#_Toc148891558)

[⮚ 5.2.2 Clonal monocytosis of undetermined significance (CMUS) and clonal cytopenia with monocytosis of undetermined significance (CCMUS) 32](#_Toc148891559)

[⮚ 5.2.3 Atypical chronic myeloid leukaemia (aCML) or MDS/MPN with
 neutrophilia 33](#_Toc148891560)

[⮚ 5.2.4 Juvenile myelomonocytic leukaemia (JMML) 36](#_Toc148891561)

[⮚ 5.2.5 MDS/MPN with *SF3B1* mutation and thrombocytosis 39](#_Toc148891562)

[⮚ 5.2.6 MDS/MPN, NOS 41](#_Toc148891563)

[6. Clonal haematopoiesis (CH) 44](#_Toc148891564)

[6.1 Cytopenia 44](#_Toc148891565)

[6.2 Clonal haematopoiesis of indeterminate potential (CHIP) 44](#_Toc148891566)

[6.3 Clonal cytopenia of undetermined significance (CCUS) 44](#_Toc148891567)

[7. Myelodysplastic syndromes/neoplasms (MDS) 45](#_Toc148891568)

[7.1 Categorisation 45](#_Toc148891569)

[7.2 Diagnostic features 46](#_Toc148891570)

[7.3 MDS/AML 50](#_Toc148891571)

[7.4 *TP53* mutation 50](#_Toc148891572)

[8. Paediatric myeloid disorders 51](#_Toc148891573)

[8.1 ICC 2022 updates 51](#_Toc148891574)

[⮚ 8.1.1 JMML, JMML-like neoplasms and Noonan syndrome-associated myeloproliferative disorder 52](#_Toc148891575)

[⮚ 8.1.2 Refractory cytopenia of childhood (RCC) 52](#_Toc148891576)

[⮚ 8.1.3 Haematological neoplasms with germline predisposition 52](#_Toc148891577)

[8.2 WHO 2022 updates 53](#_Toc148891578)

[⮚ 8.2.1 Childhood MDS with low blasts 53](#_Toc148891579)

[⮚ 8.2.2 Childhood MDS with increased blasts 53](#_Toc148891580)

[9. Acute myeloid leukaemia (AML) 53](#_Toc148891581)

[9.1 Categorisation 53](#_Toc148891582)

[9.2 Percentage of blasts required for AML diagnosis 57](#_Toc148891583)

[9.3 Therapy-related myeloid neoplasms vs. diagnostic qualifiers 58](#_Toc148891584)

[9.4 AML with myelodysplasia-related changes (AML-MR) 58](#_Toc148891585)

[9.5 Acute myeloid leukaemia, NOS 60](#_Toc148891586)

[9.6 Myeloid sarcoma 61](#_Toc148891587)

[10. Myeloid neoplasms with germline predisposition 62](#_Toc148891588)

[10.1 Diagnostic qualifiers 62](#_Toc148891589)

[10.2 Categorisation 62](#_Toc148891590)

[11. Myeloid proliferations associated with Down syndrome 65](#_Toc148891591)

[12. Conclusion 65](#_Toc148891592)

[13. Acknowledgements 66](#_Toc148891593)

[14. Supplementary appendix 66](#_Toc148891594)

# 1. Introduction

The classification of tumours of haematopoietic and lymphoid tissues is complex and constantly evolving, and plays a crucial role in the diagnosis, treatment and prognosis of patients with haematological malignancies. For decades, the World Health Organization (WHO) classification system has been widely used as a standard reference. It has been updated several times since its inception, reflecting advances in our understanding of the biology of these tumours and the development of new diagnostic and therapeutic tools.

In 2008, the fourth edition of the WHO Classification of Tumours of Hematopoietic and Lymphoid Tissues (WHO-HAEM4) was released as a collaborative effort between the European Association for Haematopathology (EAHP), the Society for Hematopathology (SH) and the WHO International Agency for Cancer Research (IARC). This fourth edition was determined by an eight-person steering committee composed of members from both societies. A clinical advisory committee (CAC) made up of haematologists, pathologists, oncologists and geneticists was convened to provide insights and reach consensus on the proposed contents. In 2017, a major update to the fourth edition (WHO-HAEM4R) involved many of the original editors as well as additional senior advisors with expertise in myeloid neoplasms and molecular/cytogenic issues. A CAC was convened to contribute to the WHO-HAEM4R as per previous editions. The final volume had more than 200 contributors from 24 countries.

Significant developments since 2017 resulted in a need for a further update. To develop the WHO fifth edition, an editorial board of *standing* members as well as *expert* members was appointed by the IARC for their leadership and expertise relevant to a particular volume. The editorial board established the draft classification (table of contents) and assigned multidisciplinary authorship groups including haematologists, oncologists, pathologists, radio-oncologists and geneticists. A clinical advisory board meeting was held and a total of 420 experts were involved in the fifth edition as either authors or editors. The resulting classification was presented in two companion manuscripts released in 2022 covering the classification of myeloid and histiocytic/dendritic neoplasms and the classification of lymphoid neoplasm as well as a beta v2 version released online.

However, the executive committees of the EAHP and SH expressed dissatisfaction with the process used to develop the fifth edition. There were concerns raised about the lack of oversight from the SH and EAHP and a failure to follow a formal CAC process as had been done before. To address this, a CAC comprising an international group of pathologists, clinicians and scientists, endorsed by SH and EAHP, separate from the WHO, was convened to update the WHO 2017 classification. This resulted in four special reports in Blood in 2022 describing the International Consensus Classification (ICC) of myeloid neoplasms, acute leukaemias and mature lymphoid neoplasms and a monographic issue of Virchows Archives in January 2023.

The aim of this document is to highlight how the latest classifications developed in 2022 have changed compared with the WHO-HAEM4R (referred to in this paper as the WHO 2017 blue book). This document will focus on updates to the classification of myeloid neoplasms and acute leukaemias and therefore will compare the 5th edition of the World Health Organization Classification of Haematolymphoid Tumours: Myeloid and Histiocytic/Dendritic Neoplasms review article in *Leukaemia* (WHO 2022)* and the International Consensus Classification of Myeloid Neoplasms and Acute Leukaemias: integrating morphologic, clinical, and genomic data article in *Blood* (ICC 2022).

The following 10 subsections are included below:

- Myeloproliferative neoplasms (MPN)
- Mastocytosis
- Myeloid/lymphoid neoplasms with eosinophilia and tyrosine kinase gene fusions
- Myelodysplastic/myeloproliferative neoplasms (MDS/MPN)
- Clonal haematopoiesis (CH)
- Myelodysplastic syndromes/neoplasms (MDS)
- Paediatric myeloid disorders
- Acute myeloid leukaemia (AML)
- Myeloid neoplasms with germline predisposition
- Myeloid proliferations associated with Down syndrome

Changes to acute leukaemias of ambiguous lineage (ALAL) are not included in this document because they were not addressed by the ICC 2022 classification working group.

*In some instances, the 5^th^ edition (beta v2) available online was used to provide additional information.

# 2. Myeloproliferative neoplasms (MPN)

MPN are clonal haematopoietic stem cell disorders characterised by the proliferation of cells of one or more of the myeloid lineages.

## 2.1 Categorisation

Myeloproliferative neoplasm entities remain largely unchanged between the WHO 2017 blue book, ICC 2022 and WHO 2022 classifications. The categories and differences between each document are listed in Table 1.

Table 1. MPN entities as defined by the WHO 2017 blue book, ICC 2022 and WHO 2022 classifications

| **ICC 2022** | **WHO 2017** | **WHO 2022** |
| --- | --- | --- |
| Chronic myeloid leukaemia | Chronic myeloid leukaemia, *BCR-ABL1*-positive | Chronic myeloid leukaemia |
| Chronic neutrophilic leukaemia | Chronic neutrophilic leukaemia | Chronic neutrophilic leukaemia |
| Polycythaemia vera | Polycythaemia vera | Polycythaemia vera |
| Primary myelofibrosis | Primary myelofibrosis | Primary myelofibrosis |
| Early/prefibrotic primary myelofibrosis | Prefibrotic/early primary myelofibrosis | Primary myelofibrosis, prefibrotic |
| Overt primary myelofibrosis | Overt primary myelofibrosis | Primary myelofibrosis, fibrotic |
| Essential thrombocythaemia | Essential thrombocythaemia | Essential thrombocythaemia |
| Chronic eosinophilic leukaemia, not otherwise specified | Chronic eosinophilic leukaemia, not otherwise specified | Chronic eosinophilic leukaemia |
| (Categorised under paediatric and/or germline mutation-associated disorders) | (Categorised under MDS/MPN) | Juvenile myelomonocytic leukaemia |
| Myeloproliferative neoplasm, unclassifiable | Myeloproliferative neoplasm, unclassifiable | Myeloproliferative neoplasm, not otherwise specified |

Updated since WHO 2017 (terminology/wording aligned between ICC 2022 and WHO 2022)

Updated since WHO 2017 (terminology/wording different between ICC 2022 and WHO 2022)

Minimal or no changes since WHO 2017

**Key**

**ICC 2022 changes to MPN entities**

- No major changes since the WHO 2017 blue book

**WHO 2022 changes to MPN entities**

- Primary myelofibrosis subtypes renamed to ‘prefibrotic’ and ‘fibrotic’
- The ‘not otherwise specified’ qualifier for chronic eosinophilic leukaemia has been omitted
- Juvenile myelomonocytic leukaemia has moved from MDS/MPN and is now categorised under MPN above
- MPN, unclassifiable entity replaced with MPN, not otherwise specified

## 2.2 Diagnostic criteria

The WHO 2022 and ICC 2022 classifications have also updated diagnostic criteria for each MPN entity since the WHO 2017 blue book was published.

### 2.2.1 Chronic myeloid leukaemia (CML)

CML is a myeloproliferative neoplasm in which granulocytes are the major proliferative component. It is characterized by the presence of the oncogenic fusion protein BCR-ABL1, the product of a t(9;22)(q34.1;q11.2) chromosomal translocation. The WHO 2017 blue book stated that ‘the natural history of untreated CML is biphasic or triphasic: an initial indolent chronic phase (CP) followed by an accelerated phase (AP), a blast phase (BP), or both’. Most patients with CML are diagnosed in CP, which usually has an insidious onset.

The WHO 2017 blue blook provides defining features of CP which remain unchanged in the WHO 2022 and ICC 2022 classifications. However, diagnostic criteria for AP and BP CML listed in the WHO 2017 blue book have been updated in the WHO 2022 and ICC 2022 classifications (Table 2).

Table 2. Diagnostic criteria for AP and BP CML as defined by the WHO 2017 blue book, ICC 2022 and WHO 2022 classifications

| **Accelerated phase CML criteria** | | |
| --- | --- | --- |
| **ICC 2022** | **WHO 2017** | **WHO 2022** |
| - Peripheral blood basophils ≥20% - Bone marrow or peripheral blood blasts 10–19% - Presence of additional clonal cytogenetic abnormality in Ph+ cells (ACA)[*](javascript:;) | Haematological/cytogenetic criteria^a^   - Persistent or increasing high white blood cell count (>10 × 10^9^/L), unresponsive to therapy - Persistent or increasing splenomegaly, unresponsive to therapy - Persistent thrombocytosis (>1000 × 10^9^/L), unresponsive to therapy - Persistent thrombocytopenia (<100 × 10^9^/L), unrelated to therapy - >20% basophils in the peripheral blood - 10–19% blasts in the peripheral blood and/or bone marrow^b,c^ - Additional clonal chromosomal abnormalities in Ph+ cells at diagnosis, including so-called major route abnormalities (a second Ph chromosome, trisomy 8, isochromosome 17q, trisomy 19), complex karyotype, and abnormalities of 3q26.2 - Any new clonal chromosomal abnormality in Ph+ cells that occurs during therapy   Provisional response-to-TKI criteria   - Haematological resistance (or failure to achieve a complete haematological response^d^) to the first TKI - Any haematological, cytogenetic or molecular indications of resistance to two sequential TKIs - Occurrence of two or more mutations in the *BCR-ABL1* fusion gene during TKI therapy | (Accelerated phase diagnostic criteria have been omitted) |
| **Blast phase CML criteria** | | |
| **ICC 2022** | **WHO 2017** | **WHO 2022** |
| - Bone marrow or peripheral blood blasts ≥20% - Myeloid sarcoma[^†^](javascript:;) - Presence of lymphoblasts (>5%) warrants consideration of lymphoblastic crisis^‡^ | - >20% blasts in the blood or bone marrow - Presence of an extramedullary proliferation of blasts | - ≥20% myeloid blasts in the blood or bone marrow - Presence of an extramedullary proliferation of blasts - Presence of increased lymphoblasts in peripheral blood or bone marrow |

Updated since WHO 2017 (terminology/wording aligned between ICC 2022 and WHO 2022)

Updated since WHO 2017 (terminology/wording different between ICC 2022 and WHO 2022)

Minimal or no changes since WHO 2017

**Key**

ACA, additional chromosome abnormality; CML, chronic myeloid leukaemia; Ph, Philadelphia chromosome; TKI, tyrosine kinase inhibitor.

*Second Ph, trisomy 8, isochromosome 17q, trisomy 19, complex karyotype or abnormalities of 3q26.2.

^†^Extramedullary blast proliferation.

^‡^Immunophenotypic analysis is required to confirm lymphoid lineage.

^a^Large clusters or sheets of small, abnormal megakaryocytes associated with marked reticulin or collagen fibrosis in biopsy specimens may be considered presumptive evidence of the accelerated phase, although these findings are usually associated with one or more of the criteria listed above.

^b^The finding of bona fide lymphoblasts in the peripheral blood or bone marrow (even if <10%) should prompt concern that lymphoblastic transformation may be imminent, and warrants further clinical and genetic investigation.

^c^>20% blasts in the peripheral blood or bone marrow, or an infiltrative proliferation of blasts in an extramedullary site, is diagnostic of the blast phase of CML.

^d^Complete haematological response is defined as white blood cell count <10 × 10^9^/L, platelet count

<450 × 10^9^/L, no immature granulocytes in the differential, and spleen not palpable.

**ICC 2022 changes to CML diagnostic criteria**

Accelerated phase

- Haematological criteria relating to white blood cell count, splenomegaly, thrombocytosis and thrombocytopenia are not considered
- Criteria concerning response to tyrosine kinase inhibitor (TKI) therapy are not considered
- Criteria relating to the presence of additional clonal cytogenetic abnormalities have been combined into one criterion

Blast phase

- An additional criterion relating to lymphoblasts has been included

**WHO 2022 changes to CML diagnostic criteria**

Accelerated phase

- AP diagnostic criteria have been omitted in favour of an emphasis on high-risk features associated with CP progression and resistance to TKI therapy. These features are listed in Supplementary Table 1.

Blast phase

- An additional criterion relating to lymphoblasts has been included

### 2.2.2 Chronic neutrophilic leukaemia (CNL)

CNL is a rare *BCR::ABL1*-negative myeloproliferative neoplasm characterised by sustained peripheral blood neutrophilia (white blood cell count ≥25 × 10^9^/L, with ≥80% segmented neutrophils and bands), bone marrow hypercellularity due to neutrophilic granulocyte proliferation, and hepatosplenomegaly. *CSF3R* mutations are common in this disease; however, additional mutations are seen in most cases.

The WHO 2017 blue book stated that the diagnosis of CNL ‘requires exclusion of reactive neutrophilia and other MPN’ and lists five main diagnostic criteria. These criteria remain largely unchanged in the WHO 2022 classification but have been updated in the ICC 2022 classification (Table 3).

Table 3. Diagnostic criteria for CNL as defined by the WHO 2017 blue book, ICC 2022 and WHO 2022 classifications

| **ICC 2022** | **WHO 2017** | **WHO 2022** |
| --- | --- | --- |
| 1. - Peripheral blood white blood cell count ≥13 × 10^9^/L[*](javascript:;)  - Segmented neutrophils plus banded neutrophils constitute ≥80% of the white blood cells    - Neutrophil precursors (promyelocytes, myelocytes and metamyelocytes) constitute <10% of the white blood cells  - Circulating blasts only rarely observed  - Monocyte count <10% of all leukocytes[†](javascript:;)  - No significant dysgranulopoiesis | 1. - Peripheral blood white blood cell count ≥25 × 10^9^/L  - Segmented neutrophils plus banded neutrophils constitute ≥80% of the white blood cells  - Neutrophil precursors (promyelocytes, myelocytes and metamyelocytes) constitute <10% of the white blood cells  - Myeloblasts rarely observed  - Monocyte count <1 × 10^9^/L  - No dysgranulopoiesis | 1. - Peripheral blood white blood cell count ≥25 × 10^9^/L  - Segmented neutrophils plus banded neutrophils constitute ≥80% of the white blood cells  - Neutrophil precursors (promyelocytes, myelocytes and metamyelocytes) constitute <10% of the white blood cells  - Myeloblasts rarely observed  - Monocytes constitute <10% of peripheral blood leukocytes; absolute monocytosis not meeting criteria for CMML  -  No dysgranulopoiesis |
| 2. Hypercellular bone marrow with neutrophil granulocytes increased in percentage and absolute number, showing normal maturation | 2. - Hypercellular bone marrow  - Neutrophil granulocytes increased in percentage and number  - Neutrophil maturation appears normal  - Myeloblasts constitute <5% of the nucleated cells | 2. - Hypercellular bone marrow  - Neutrophil granulocytes increased in percentage and number  - Neutrophil maturation appears normal  - Myeloblasts constitute <5% of the nucleated cells |
| 3. *CSF3R* T618I or another activating *CSF3R* mutation or persistent neutrophilia  (≥3 months), splenomegaly, and no identifiable cause of reactive neutrophilia, including absence of a plasma cell neoplasm or, if a plasma cell neoplasm is present, demonstration of clonality of myeloid cells by cytogenetic or molecular studies | 3. Not meeting WHO criteria for *BCR-ABL1*-positive CML, PV, ET or PMF | 3. Not meeting WHO criteria for *BCR-ABL1*-positive CML, PV, ET or PMF |
| 4. Not meeting diagnostic criteria for *BCR::ABL1*-positive CML, PV, ET, PMF or of a myeloid/lymphoid neoplasm with eosinophilia and tyrosine kinase gene fusions | 4. No rearrangement of *PDGFRA*, *PDGFRB* or *FGFR1*, and no *PCM1-JAK2* fusion | 4. No evidence of disease-defining gene rearrangement of *PDGFRA*, *PDGFRB* or *FGFR1*, and no *PCM1::JAK2* fusion |
|  | 5. *CSF3R* T618I or another activating *CSF3R* mutation  or  Persistent neutrophilia  (≥3 months), splenomegaly and no identifiable cause of reactive neutrophilia including absence of a plasma cell neoplasm or, if a plasma cell neoplasm is present, demonstration of clonality of myeloid cells by cytogenetic or molecular studies | 5. Presence of CSF3R p.T618I or another activating *CSF3R* mutation  or  Persistent neutrophilia  (≥3 months), splenomegaly and no identifiable cause of reactive neutrophilia including absence of a plasma cell neoplasm or, if a plasma cell neoplasm is present, demonstration of clonality of myeloid cells by cytogenetic or molecular studies |

Updated since WHO 2017 (terminology/wording aligned between ICC 2022 and WHO 2022)

Updated since WHO 2017 (terminology/wording different between ICC 2022 and WHO 2022)

Minimal or no changes since WHO 2017

**Key**

CML, chronic myeloid leukaemia; CNL, chronic neutrophilic leukaemia; PV, polycythaemia vera; ET, essential thrombocythaemia; PMF, primary myelofibrosis.

*At least 25 × 10^9^/L in cases lacking *CSF3R* T618I or another activating *CSF3R* mutation.

^†^10–19% blasts in peripheral blood or bone marrow represent CNL in accelerated phase; ≥20% blasts represents blast phase.

**ICC 2022 changes to CNL diagnostic criteria**

- Key diagnostic threshold for leukocytosis has been lowered from ≥25 to ≥13 × 10^9^/L in cases with *CSF3R* T618I or another activating *CSF3R* mutation
- The criterion related to monocyte count has been updated
- ‘Absence of *PDGFRA*, *PDGFRB* or *FGFR1* rearrangement, or of *PCM1-JAK2* fusion’ removed as a diagnostic criterion
- The order of the criteria has been updated

**WHO 2022 changes to CNL diagnostic criteria**

- The criterion related to monocyte count has been updated

**Polycythaemia vera, primary myelofibrosis and essential thrombocythaemia**

These are all *BCR::ABL1*-negative MPN with overlapping disease characteristics. Distinguishing between these entities can therefore be challenging but is important for identifying optimal treatment strategies and for patient prognosis.

### 2.2.3 Polycythaemia vera (PV)

PV is characterised by an overproduction of red blood cells independent of the mechanisms that normally regulate erythropoiesis. The majority of PV patients carry the *JAK2* V617F mutation or the *JAK2* exon 12 mutation. It is widely recognised that there are two phases of PV – the polycythaemic phase and the post-polycythaemic myelofibrotic (post-PV MF) phase.

The WHO 2017 blue book provided diagnostic criteria for PV and post-PV MF phases. Small refinements have been made to the diagnostic criteria for each PV phase in both the ICC 2022 and WHO 2022 classifications (Table 4).

Table 4. Diagnostic criteria for PV and post-PV MF as defined by the WHO 2017 blue book, ICC 2022 and WHO 2022 classifications

| **PV phase criteria** | | |
| --- | --- | --- |
| **ICC 2022** | **WHO 2017** | **WHO 2022** |
| Major criteria  1. Elevated haemoglobin concentration or elevated haematocrit or increased red blood cell mass*  2. Presence of JAK2 V617F or JAK2 exon 12 mutation^†^  3. Bone marrow biopsy showing age-adjusted hypercellularity with trilineage proliferation (panmyelosis), including prominent erythroid, granulocytic, and increase in pleomorphic, mature megakaryocytes without atypia | Major criteria  1. Elevated haemoglobin concentration (>16.5 g/dL in men; >16.0 g/dL in women) or  Elevated haematocrit (>49% in men; >48% in women) or  Increased red blood cell mass (>25% above mean normal predicted value)  2. Bone marrow biopsy showing age-adjusted hypercellularity with trilineage growth (panmyelosis),  including prominent erythroid, granulocytic, and megakaryocytic proliferation with pleomorphic,  mature megakaryocytes (differences in size)^a^  3. Presence of *JAK2* V617F or *JAK2* exon 12 mutation | Major criteria  1. Elevated haemoglobin concentration (>16.5 g/dL in men; >16.0 g/dL in women) or  elevated haematocrit (>49% in men; >48% in women)^b^  2. Bone marrow biopsy showing age-adjusted hypercellularity with trilineage growth (panmyelosis),  including prominent erythroid, granulocytic and megakaryocytic proliferation with pleomorphic,  mature megakaryocytes (differences in size)^c^  3. Presence of *JAK2* V617F or *JAK2* exon 12 mutation |
| Minor criterion  Subnormal serum erythropoietin level | Minor criterion  Subnormal serum erythropoietin level | Minor criterion  Subnormal serum erythropoietin level |
| *The diagnosis of PV requires either all three major criteria*  *or the first two major criteria plus the minor criterion^‡^* | | |
| **Post-PV MF phase criteria** | | |
| **ICC 2022** | **WHO 2017** | **WHO 2022** |
| Required criteria 1. Previous established diagnosis of PV  2. Bone marrow fibrosis of grade 2 or 3 | Required criteria 1. Documentation of a previous diagnosis of WHO-defined PV  2. Bone marrow fibrosis of grade 2–3 on a 0–3 scale or grade 3–4 on a 0–4 scale | Required criteria 1. Documentation of a previous diagnosis of WHO-defined PV  2. Bone marrow fibrosis of grade 2–3 on a 0–3 scale |
| Additional criteria  1. Anaemia (i.e. below the reference range given age, sex and altitude considerations) or sustained loss of requirement of either phlebotomy (in the absence of cytoreductive therapy) or cytoreductive treatment for erythrocytosis  2. Leukoerythroblastosis  3. Increase in palpable splenomegaly of >5 cm from baseline or the development of a newly palpable splenomegaly  4. Development of any two  (or all three) of the following constitutional symptoms: >10% weight loss in 6 months, night sweats, unexplained fever (>37.5°C) | Additional criteria  1. Anaemia (i.e. below the reference range given age, sex and altitude considerations) or sustained loss of requirement of either phlebotomy (in the absence of cytoreductive therapy) or cytoreductive treatment for erythrocytosis  2. Leukoerythroblastosis  3. Increasing splenomegaly, defined as either an increase in palpable splenomegaly of  >5 cm from baseline (distance from the left costal margin) or the development of a newly palpable splenomegaly  4. Development of any two  (or all three) of the following constitutional symptoms: >10% weight loss in 6 months, night sweats, unexplained fever (>37.5°C) | Additional criteria  1. Anaemia (i.e. below the reference range given age, sex and altitude considerations) or sustained loss of requirement of either phlebotomy (in the absence of cytoreductive therapy) or cytoreductive treatment for erythrocytosis  2. Leukoerythroblastosis  3. Increasing splenomegaly, defined as either an increase in palpable splenomegaly of  >5 cm from baseline (distance from the left costal margin) or the development of a newly palpable splenomegaly  4. Development of any two  (or all three) of the following constitutional symptoms: >10% weight loss in 6 months, night sweats, unexplained fever (>37.5°C) |
| *The diagnosis of post-PV MF is established by all required criteria and at least two additional criteria* | | |

Updated since WHO 2017 (terminology/wording aligned between ICC 2022 and WHO 2022)

Updated since WHO 2017 (terminology/wording different between ICC 2022 and WHO 2022)

Minimal or no changes since WHO 2017

**Key**

*Diagnostic thresholds: haemoglobin: >16.5 g/dL in men and >16.0 g/dL in women; haematocrit: >49% in men and >48% in women; red blood cell mass: >25% above mean normal predicted value.

^†^It is recommended to use highly sensitive assays for *JAK2*V617F (sensitivity level < 1%); in negative cases, consider searching for non-canonical or atypical *JAK2* mutations in exons 12 to 15.

^‡^A bone marrow biopsy may not be required in patients with sustained absolute erythrocytosis (haemoglobin concentrations of >18.5 g/dL in men or >16.5 g/dL in women and haematocrit values of >55.5% in men or >49.5% in women) and the presence of a *JAK2*V617F or *JAK2* exon 12 mutation.

^a^Major criterion 2 (bone marrow biopsy) may not be required in patients with sustained absolute erythrocytosis (haemoglobin concentrations of >18.5 g/dL in men or >16.5 g/dL in women or haematocrit values of >55.5% in men or >49.5% in women), if major criterion 3 and the minor criterion are present. However, initial myelofibrosis (present in as many as 20% of patients) can only be detected by bone marrow biopsy, and this finding may predict a more rapid progression to overt myelofibrosis (post-PV myelofibrosis)

^b^Haematocrit for diagnosis in the absence of a *JAK2* mutation. A higher haematocrit target could be considered (e.g. 0.52) in men before further investigation may be required.

^c^Major criterion 2 (bone marrow biopsy) may not be required in patients with sustained absolute erythrocytosis (haemoglobin concentrations of >18.5 g/dL in men or >16.5 g/dL in women or haematocrit values of >0.555 in men or >0.495 in women), if major criterion 3 and the minor criterion are present.

**ICC 2022 changes to PV diagnostic criteria**

Polycythaemic phase

- The order of the three major criteria has been updated
- A recommendation on how to detect presence of *JAK2* mutations has been added
- The definition of trilineage proliferation seen on a bone marrow biopsy has been adjusted
- The scenario where a biopsy may not be required has been updated

Post-PV MF phase

- The bone marrow fibrosis criterion has been updated

**WHO 2022 changes to PV diagnostic criteria**

Polycythaemic phase

- Increased red blood cell mass has been removed as a diagnostic criterion

Post-PV MF phase

- The bone marrow fibrosis criterion has been updated

### 2.2.4 Primary myelofibrosis (PMF)

PMF is characterised by a proliferation of abnormal megakaryocytes and granulocytes in the bone marrow, which in fully developed disease is associated with bone marrow reticulin/collagen fibrosis, osteosclerosis and extramedullary haematopoiesis. There are two stages of PMF – an early, pre-fibrotic stage and an overt fibrotic stage. Both the ICC 2022 and the WHO 2022 classifications highlight the importance of distinguishing between pre-fibrotic PMF, ET, PV and fibrotic PMF.

The diagnostic criteria for each PMF stage listed in the WHO 2017 blue book remain largely unchanged in the ICC 2022 and WHO 2022 classifications.

**ICC 2022 changes to PMF diagnostic criteria**

- No major changes since the WHO 2017 blue book

**WHO 2022 changes to PMF diagnostic criteria**

- No major changes since the WHO 2017 blue book

### 2.2.5 Essential thrombocythaemia (ET)

ET is characterised by sustained thrombocytosis (platelet count ≥450 × 10^9^/L) in the peripheral blood and increased numbers of large, mature megakaryocytes in the bone marrow. Thrombosis and/or haemorrhage are common in patients with ET. After many years, around 10% of patients will develop bone marrow fibrosis (post-ET MF).

The WHO 2017 blue book provided diagnostic criteria for both ET and post-ET MF. These criteria remain largely unchanged in the WHO 2022 classification but have been refined in the ICC 2022 classification (Table 5).

Table 5. Diagnostic criteria for ET and post-ET MF as defined by the WHO 2017 blue book, ICC 2022 and WHO 2022 classifications

| **ET phase criteria** | | |
| --- | --- | --- |
| **ICC 2022** | **WHO 2017** | **WHO 2022** |
| Major criteria  1. Platelet count ≥450 × 10^9^/L  2. Bone marrow biopsy showing proliferation mainly of the megakaryocytic lineage, with increased numbers of enlarged, mature megakaryocytes with hyperlobulated staghorn-like nuclei, infrequently dense clusters*; no significant increase or left shift in neutrophil granulopoiesis or erythropoiesis; no relevant BM fibrosis^†^  3. Diagnostic criteria for BCR::ABL1-positive CML, PV, PMF, or other myeloid neoplasms are not met  4. *JAK2*, *CALR* or *MPL* mutation^‡^ | Major criteria  1. Platelet count ≥450 × 10^9^/ L  2. Bone marrow biopsy showing proliferation mainly of the megakaryocytic lineage, with increased numbers of enlarged, mature megakaryocytes with hyperlobulated nuclei; no significant increase or left shift in neutrophil granulopoiesis or erythropoiesis; very rarely a minor (grade 1^a^) increase in reticulin fibres  3. WHO criteria for BCR::ABL1-positive CML, PV, PMF, or other myeloid neoplasms are not met  4. *JAK2*, *CALR* or *MPL* mutation | Major criteria  1. Platelet count ≥450 × 10^9^/ L  2. Bone marrow biopsy showing proliferation mainly of the megakaryocytic lineage, with increased numbers of enlarged, mature megakaryocytes with hyperlobulated nuclei; no significant increase or left shift in neutrophil granulopoiesis or erythropoiesis; very rarely a minor (grade 1) increase in reticulin fibres  3. WHO criteria for BCR::ABL1-positive CML, PV, PMF, or other myeloid neoplasms are not met  4. *JAK2*, *CALR* or *MPL* mutation |
| Minor criterion  Presence of a clonal marker^§^ or absence of evidence of reactive thrombocytosis^ǁ^ | Minor criterion  Presence of a clonal marker or exclusion of reactive thrombocytosis | Minor criterion  Presence of a clonal marker or exclusion of reactive thrombocytosis |
| *The diagnosis of ET requires either all major criteria or the first three major criteria*  *plus the minor criterion* | | |
| **Post-ET MF phase criteria** | | |
| **ICC 2022** | **WHO 2017** | **WHO 2022** |
| Required criteria 1. Previous established diagnosis of ET  2. Bone marrow fibrosis of grade 2 or 3 | Required criteria 1. Documentation of a previous diagnosis of WHO-defined ET  2. Bone marrow fibrosis of grade 2–3 on a 0–3 scale or grade 3–4 on a 0–4 scale | Required criteria 1. Documentation of a previous diagnosis of WHO-defined ET  2. Bone marrow fibrosis of grade 2–3 on a 0–3 scale |
| Additional criteria  1. Anaemia (i.e. below the reference range given age, sex and altitude considerations) and a >2 g/dL decrease from baseline haemoglobin concentration  2. Leukoerythroblastosis  3. Increase in palpable splenomegaly of >5 cm from baseline or the development of a newly palpable splenomegaly  4. Elevated LDH level above the reference range  5. Development of any two  (or all three) of the following constitutional symptoms: >10% weight loss in 6 months, night sweats, unexplained fever (>37.5°C) | Additional criteria  1. Anaemia (i.e. below the reference range given age, sex and altitude considerations) and a >2 g/dL decrease from baseline haemoglobin concentration  2. Leukoerythroblastosis  3. Increasing splenomegaly, defined as either an increase in palpable splenomegaly of  >5 cm from baseline (distance from the left costal margin) or the development of a newly palpable splenomegaly  4. Elevated LDH level (above the reference range)  4. Development of any two  (or all three) of the following constitutional symptoms: >10% weight loss in 6 months, night sweats, unexplained fever (>37.5°C) | Additional criteria  1. Anaemia (i.e. below the reference range given age, sex and altitude considerations) and a >2 g/dL decrease from baseline haemoglobin concentration  2. Leukoerythroblastosis  3. Increasing splenomegaly, defined as either an increase in palpable splenomegaly of  >50 mm from baseline (distance from the left costal margin, or on imaging) or the development of a newly palpable splenomegaly  4. Elevated LDH level (above the reference range)  4. Development of any two  (or all three) of the following constitutional symptoms: >10% weight loss in 6 months, night sweats, unexplained fever (>37.5°C) |
| *The diagnosis of post-ET MF is established by all required criteria and at least two additional criteria* | | |

Updated since WHO 2017 (terminology/wording aligned between ICC 2022 and WHO 2022)

Updated since WHO 2017 (terminology/wording different between ICC 2022 and WHO 2022)

Minimal or no changes since WHO 2017

**Key**

CML, chronic myeloid leukaemia; ET, essential thrombocythaemia; MF, myelofibrosis; LDH, lactate dehydrogenase; PMF, primary myelofibrosis; PV, polycythaemia vera.

*Three or more megakaryocytes lying adjacent without other BM cells in between; in most of these rare clusters ≤6 megakaryocytes may be observed, increase in huge clusters (>6 cells) accompanied by granulocytic proliferation is a morphological hallmark of pre-PMF.

^†^Very rarely a minor increase in reticulin fibres may occur at initial diagnosis (grade 1).

^‡^It is recommended to use highly sensitive assays for JAK2 V617F (sensitivity level < 1%) and CALR and MPL (sensitivity level 1% to 3%); in negative cases, consider a search for noncanonical JAK2 and MPL mutations.

^§^Assessed by cytogenetics or sensitive NGS techniques.

^ǁ^Reactive causes of thrombocytosis include a variety of underlying conditions like iron deficiency, chronic infection, chronic inflammatory disease, medication, neoplasia, or history of splenectomy.

^a^See Supplementary Table 2.

**ICC 2022 changes to ET diagnostic criteria**

ET

- The major criterion relating to the proliferation mainly of the megakaryocytic lineage has been updated

Post-ET MF

- The bone marrow fibrosis criterion has been updated

**WHO 2022 changes to ET diagnostic criteria**

ET

- No major changes since the WHO 2017 blue book

Post-ET MF

- The bone marrow fibrosis criterion has been updated

### 2.2.6 Chronic eosinophilic leukaemia, not otherwise specified (CEL, NOS)

CEL, NOS (or CEL in WHO 2022 classification) is characterised by persistent eosinophilia (clonal proliferation of eosinophilic precursors resulting in a persistently elevated number of eosinophils in blood, bone marrow or peripheral tissues) not meeting the criteria for other genetically defined entities. It can be challenging to define clonality and therefore distinguish CEL from other eosinophilic disorders (e.g. idiopathic hypereosinophilic syndromes [iHES] and hypereosinophilia of unknown significance [HEus]).

The WHO 2017 blue book provided diagnostic criteria for CEL, NOS which have since been updated in the WHO 2022 and ICC 2022 classifications (Table 6). Unlike the WHO 2022 classification, the ICC 2022 classification also provides separate diagnostic criteria for iHES (Table 7).

Table 6. Diagnostic criteria for CEL, NOS (or CEL) as defined by the WHO 2017 blue book, ICC 2022 and WHO 2022 classifications

| **ICC 2022** | **WHO 2017** | **WHO 2022** |
| --- | --- | --- |
| 1. Peripheral blood hypereosinophilia (eosinophil count ≥1.5 × 10^9^/L and eosinophils ≥10% of white blood cells) | 1. Eosinophilia (eosinophil count ≥1.5 × 10^9^/L) | 1. Hypereosinophilia defined as peripheral blood eosinophilia ≥1.5 × 10^9^/L on at least two occasions over an interval of at least 4 weeks |
| 2. Blasts constitute <20% cells in peripheral blood and bone marrow, not meeting other diagnostic criteria for AML* | 2. WHO criteria for *BCR-ABL1*-positive CML, PV, ET, PMF CNL, CMML and *BCR-ABL1*-negative atypical CML are not met | 2. Evidence of clonality^b^ |
| 3. No tyrosine kinase gene fusion including BCR::ABL1, other ABL1, PDGFRA, PDGFRB, FGFR1, JAK2 or FLT3 fusions | 3. No rearrangement of *PDGFRA*, *PDGFRB* or *FGFR1*, and no *PCM1-JAK2*, *ETV6-JAK2* or *BCR-JAK2* fusion | 3. Abnormal bone marrow morphology (e.g. megakaryocytic or erythroid dysplasia) |
| 4. Not meeting criteria for other well-defined MPN, chronic myelomonocytic leukaemia or SM^†^ | 4. Blasts constitute <20% cells in peripheral blood and bone marrow, and inv(16)(p13.1q22),  t(16;16)(p13.1;q22), t(8;21)(q22;q22.1) and other diagnostic features of AML are absent | 4. WHO criteria for other myeloid or lymphoid neoplasms (including MPN, MDS/MPN, MDS, myeloid/lymphoid neoplasms with eosinophilia and tyrosine kinase gene fusions, mastocytosis and AML) are not met |
| 5. Bone marrow shows increased cellularity with dysplastic megakaryocytes with or without dysplastic features in other lineages and often significant fibrosis, associated with an eosinophilic infiltrate or increased blasts ≥5% in the bone marrow and/or ≥2% in the peripheral blood | 5. There is a clonal cytogenetic or molecular genetic abnormality^a^  or  Blast cells account for ≥2% of cells in the peripheral blood or ≥5% in the bone marrow |  |
| 6. Demonstration of a clonal cytogenetic abnormality and/or somatic mutation(s)^‡^ |  |  |

Updated since WHO 2017 (terminology/wording aligned between ICC 2022 and WHO 2022)

Updated since WHO 2017 (terminology/wording different between ICC 2022 and WHO 2022)

Minimal or no changes since WHO 2017

**Key**

AML, acute myeloid leukaemia; CEL, chronic eosinophilic leukaemia; CHIP, clonal haematopoiesis of indeterminate potential; CML, chronic myeloid leukaemia; CMML, chronic myelomonocytic leukaemia; CNL, chronic neutrophilic leukaemia; ET, essential thrombocythaemia; MDS, myelodysplastic neoplasms; MDS/MPN, myelodysplastic/myeloproliferative neoplasms; MPN, myeloproliferative neoplasms; NOS, not otherwise specified; PMF, primary myelofibrosis; PV, polycythaemia vera; SM, systemic mastocytosis.

*AML with recurrent genetic abnormalities with <20% blasts is excluded.

^†^Eosinophilia can be seen in association with SM. However, “true” CEL, NOS may occur as SM-AMN (SM with an associated myeloid neoplasm).

^‡^In the absence of a clonal cytogenetic abnormality and/or somatic mutation(s) or increased blasts, bone marrow findings supportive of the diagnosis will suffice in the presence of persistent eosinophilia, provided other causes of eosinophilia having been excluded.

^a^Because some clonal molecular genetic abnormalities (e.g. mutations in *TET2*, *ASXL1* and *DNMT3A*) can occur in a minority of elderly people in the absence of any apparent haematological abnormality, it is essential to exclude all possible causes of reactive eosinophilia before making this diagnosis solely on the basis of a molecular genetic abnormality in an elderly person.

^b^The possibility of CHIP should be considered.

**ICC 2022 changes to CEL, NOS diagnostic criteria**

- The order of the diagnostic criteria has been updated
- The definition of eosinophilia has been expanded
- The criterion relating to gene fusion/rearrangement has been updated
- The criterion relating to the exclusion of other myeloid neoplasms has been updated to include systemic mastocytosis (SM)
- Clonal cytogenetic abnormality and abnormal bone marrow morphology are included as two separate criteria
- Diagnostic criteria for iHES have been included (Table 7)

**WHO 2022 changes to CEL diagnostic criteria**

- The time interval required to define sustained hypereosinophilia is reduced from
  6 months to 4 weeks
- The criterion relating to the exclusion of other myeloid neoplasms has been updated
- A requirement for both clonality and abnormal bone marrow morphology has been added
- The criterion relating to increased blasts (≥2% in peripheral blood or 5–19% in bone marrow) as an alternative to clonality has been eliminated

Table 7. Diagnostic criteria for iHES as defined by the ICC 2022 classification

| **ICC 2022 diagnostic criteria for iHES** |
| --- |
| 1. Persistent peripheral blood hypereosinophilia (eosinophil count ≥1.5 × 10^9^/L and ≥10% eosinophils)* |
| 2. Organ damage and/or dysfunction attributable to tissue eosinophilic infiltrate^†^ |
| 3. No evidence of a reactive, well-defined autoimmune disease or neoplastic condition/disorder underlying the hypereosinophilia |
| 4. Exclusion of lymphocyte variant hypereosinophilic syndrome^‡^ |
| 5. Bone marrow morphologically within normal limits except for increased eosinophils |
| 6. No molecular genetic clonal abnormality, with the caveat of clonal haematopoiesis of indeterminate potential (CHIP) |
| *The diagnosis of iHES requires all six criteria.* |

*Preferably a minimal duration of 6 months if documentation is available.

^†^Hypereosinophilia of uncertain significance has no tissue damage, but otherwise fulfils the same diagnostic criteria.

^‡^An abnormal T-cell population must be detected immunophenotypically with or without T-cell receptor clonality by molecular analysis.

### 2.2.7 Myeloproliferative neoplasm, unclassifiable (MPN-U)

MPN-U or MPN, NOS (WHO 2022 entity) is a designation that should only be applied to cases with clinical, laboratory, morphologic and molecular features of MPN but fail to meet the diagnostic criteria of any specific MPN type, or that present with features that overlap between two or more distinct MPN types.

The WHO 2017 blue book provided diagnostic criteria for MPN-U which have been updated in the ICC 2022 and WHO 2022 classifications (Table 8).

Table 8. Diagnostic criteria for MPN-U (or MPN, NOS) as defined by the WHO 2017 blue book, ICC 2022 and WHO 2022 classifications

| **ICC 2022** | **WHO 2017** | **WHO 2022** |
| --- | --- | --- |
| 1. Clinical and haematological features of an MPN are present* | 1. Features of an MPN are present | 1. Presence of features of an MPN^d^ |
| 2. JAK2, CALR or MPL mutation^†^ or presence of another clonal marker^‡^ | 2. WHO criteria for any other MPN, MDS^a^, MDS/MPN^a^ or *BCR-ABL1*-positive CML are not met | 2. WHO criteria for any other MPN, MDS, MDS/MPN^a^ or *BCR::ABL1*-positive CML are not met, negative for *PDGFRA*, *PDGFRB, FGFR1, JAK2* fusions, *ETV6::ABL1* and other *ABL1* rearrangements |
| 3. Diagnostic criteria for any other MPN, MDS, MDS/MPN,^§^ or BCR::ABL1-positive CML are not met | 3. *JAK2*, *CALR* or *MPL* mutation characteristically associated with MPN  or  Presence of another clonal marker^b^  or  Absence of a cause of reactive fibrosis^c^ | 3. Presence of driver mutations such as *JAK2, CALR* or *MPL* mutation, or another clonal marker^e^ |
| *The diagnosis of MPN-U (or MPN, NOS) requires all three criteria* | | |
|  |  | *The WHO 2022 classification also requires the absence of both these criteria:*  1. Insufficient clinical data or inadequate bone marrow specimen for accurate evaluation and classification  2. Recent history of cytotoxic or growth factor therapy, particularly when dysplastic features are seen |

Updated since WHO 2017 (terminology/wording aligned between ICC 2022 and WHO 2022)

Updated since WHO 2017 (terminology/wording different between ICC 2022 and WHO 2022)

Minimal or no changes since WHO 2017

**Key**

CEL, chronic eosinophilic leukaemia; CML, chronic myeloid leukaemia; MDS, myelodysplastic neoplasms; MDS/MPN, myelodysplastic/myeloproliferative neoplasms; MPN, myeloproliferative neoplasms; NGS, next-generation sequencing; NOS, not otherwise specified; U, unclassifiable.

*In cases presenting with bone marrow fibrosis reactive causes must be excluded, in particular bone marrow fibrosis secondary to infection, autoimmune disorder or another chronic inflammatory condition, hairy cell leukaemia or another lymphoid neoplasm, metastatic malignancy or toxic (chronic) myelopathy.

^†^It is recommended to use highly sensitive assays for *JAK2* V617F (sensitivity level <1%) and *CALR* and *MPL* (sensitivity level 1% to 3%); in negative cases, consider searching for non-canonical *JAK2* and *MPL* mutations.

^‡^Assessed by cytogenetics or sensitive NGS techniques; detection of mutations associated with myeloid neoplasms (e.g. *ASXL1*, *EZH2*, *IDH1*, *IDH2*, *SF3B1*, *SRSF2* and *TET2* mutations) supports the clonal nature of the disease.

^§^In cases presenting with myelodysplastic features effects of any previous treatment, severe comorbidity, and changes during the natural progression of the disease process must be carefully excluded.

^a^Effects of any previous treatment, severe comorbidity and changes during the natural progression of the disease process must be excluded.

^b^In the absence of any of the three major clonal mutations, a search for other mutations associated with myeloid neoplasms (e.g. *ASXL1*, *EZH2*, *TET2*, *IDH1*, *IDH2*, *SRSF2* and *SF3B1* mutations) may be of help in confirming the clonal nature of a suspected MPN, unclassifiable.

^c^Bone marrow fibrosis secondary to infection, autoimmune disorder or another chronic inflammatory condition, hairy cell leukaemia or another lymphoid neoplasm, metastatic malignancy, or toxic (chronic) myelopathy.

^d^MPN features include either one of the following:

- Clinical: Splenomegaly, atypical thrombosis, leukocytosis, in the absence of significant monocytosis and significant eosinophilia (not meeting criteria for CEL);
- Bone marrow morphology features of atypical megakaryocytic hyperplasia in a hypercellular marrow, panmyelosis, in the absence of dysplastic features (not meeting criteria for MDS or MDS/MPN);
- Clinical and morphological features can be discrepant. In the pathology report, it is important to describe the morphological findings, summarise the reasons for the difficulty in the classification of a specific MPN subtype, specify any particular subtypes that can be excluded, and recommend additional workup such as expanded molecular testing or repeat peripheral blood/bone marrow within a reasonable duration for further classification.

^e^In the absence of any of the three major clonal mutations, a search for other mutations associated with myeloid neoplasms (e.g. *ASXL1, EZH2, TET2, IDH1, IDH2, SRSF2* and *SF3B1* mutations) and translocations such as those involving ABL1 may be of help in confirming the clonal nature of a suspected MPN, NOS.

**ICC 2022 changes to MPN-U diagnostic criteria**

- The order of the diagnostic criteria has been updated
- ‘Absence of a cause of reactive fibrosis’ has been omitted from the third criterion

**WHO 2022 changes to MPN, NOS diagnostic criteria**

- The second criterion has been updated to include no rearrangement of *PDGFRA, PDGFRB*, or *FGFR1*; and no fusions involving *JAK2 or ABL1*
- ‘Absence of a cause of reactive fibrosis’ has been omitted from the third criterion
- The absence of two additional criteria is a new requirement

# 3. Mastocytosis

Mastocytosis is a neoplastic disease characterised by infiltration of clonal mast cells in one or more organ system. The disorder is heterogenous with manifestations ranging from skin lesions that can spontaneously regress to highly aggressive neoplasms associated with multiorgan failure.

## 3.1 Categorisation

The WHO 2017 blue book classified three main types of mastocytosis variant: cutaneous mastocytosis, systemic mastocytosis and mast cell sarcoma. In the WHO 2022 classification, these three main types remain the same and additional subtypes have been included. The ICC 2022 classification has also been updated and mast cell sarcoma has been omitted as a main type. The differences between each document are listed in Table 9.

Table 9. Mastocytosis entities as defined by the WHO 2017 blue book, ICC 2022 and WHO 2022 classifications

| **ICC 2022** | **WHO 2017** | **WHO 2022** |
| --- | --- | --- |
| **Cutaneous mastocytosis** | **Cutaneous mastocytosis** | **Cutaneous mastocytosis** |
|  |  | Urticaria pigmentosa/maculopapular cutaneous mastocytosis   - - Monomorphic   - Polymorphic |
|  |  | Diffuse cutaneous mastocytosis |
|  |  | Cutaneous mastocytoma   - - Isolated mastocytoma   - Multilocalised mastocytoma |
| **Systemic mastocytosis** | **Systemic mastocytosis** | **Systemic mastocytosis** |
|  |  | Bone marrow systemic mastocytosis |
| Indolent systemic mastocytosis | Indolent systemic  mastocytosis | Indolent systemic mastocytosis |
| Smoldering systemic mastocytosis |  | Smoldering systemic mastocytosis |
| Systemic mastocytosis with an associated myeloid neoplasm | Systemic mastocytosis with an associated haematological neoplasm | Systemic mastocytosis with an associated haematological neoplasm |
| Aggressive systemic mastocytosis | Aggressive systemic mastocytosis | Aggressive systemic mastocytosis |
| Mast cell leukaemia | Mast cell leukaemia | Mast cell leukaemia |
| **Mast cell sarcoma** | **Mast cell sarcoma** | **Mast cell sarcoma** |

Updated since WHO 2017 (terminology/wording aligned between ICC 2022 and WHO 2022)

Updated since WHO 2017 (terminology/wording different between ICC 2022 and WHO 2022)

Minimal or no changes since WHO 2017

**Key**

**ICC 2022 changes to mastocytosis entities**

- Smoldering systemic mastocytosis has been added as a new subtype
- Systemic mastocytosis with an associated haematological neoplasm is changed to systemic mastocytosis with an associated myeloid neoplasm

**WHO 2022 changes to mastocytosis entities**

- Three subtypes of cutaneous mastocytosis have been specified
- Bone marrow systemic mastocytosis and smoldering systemic mastocytosis have been added as new subtypes

## 3.2 Diagnostic criteria

Some minor refinements to the diagnostic criteria for mastocytosis have been made in both the WHO 2022 and ICC 2022 classifications.

### 3.2.1 Cutaneous mastocytosis

Cutaneous mastocytosis is almost always confined to the skin and mainly affects children. The WHO 2017 blue book and ICC 2022 classification recognise three major variants of cutaneous mastocytosis: urticaria pigmentosa/maculopapular cutaneous mastocytosis, diffuse cutaneous mastocytosis and mastocytoma of skin. These variants have been included in the WHO 2022 classification as specific subtypes. Additional sub-classifications of urticaria pigmentosa/maculopapular cutaneous mastocytosis (monomorphic or polymorphic) and cutaneous mastocytoma (isolated or multilocalised) are also included in the WHO 2022 document.

No major changes to the diagnostic criteria for cutaneous mastocytosis have been made since 2017.

### 3.2.2 Systemic mastocytosis (SM)

SM is characterised by the involvement of at least one extracutaneous organ, with or without evidence of skin lesions. This disorder is usually diagnosed after the second decade of life.

Since the WHO 2017 blue book was published, the ICC 2022 and WHO 2022 classifications have updated the SM diagnostic criteria (Table 10). The ICC 2022 classification also includes specific diagnostic criteria for systemic mastocytosis with an associated myeloid neoplasm (SM-AMN) (Table 11).

Table 10. Diagnostic criteria for SM as defined by the WHO 2017 blue book, ICC 2022 and WHO 2022 classifications

| **ICC 2022** | **WHO 2017** | **WHO 2022** |
| --- | --- | --- |
| Major criterion  Multifocal dense infiltrates of tryptase- and/or CD117-positive mast cells (≥15 mast cells in aggregates) detected in sections of bone marrow and/or other extracutaneous organ(s)[*](javascript:;) | Major criterion  Multifocal dense infiltrates of mast cells (≥15 mast cells in aggregates) detected in sections of bone marrow and/or other extracutaneous organ(s) | Major criterion  Multifocal dense infiltrates of mast cells (≥15 mast cells in aggregates) detected in sections of bone marrow and/or other extracutaneous organ(s) |
| Minor criteria   - In bone marrow biopsy or in section of other extracutaneous organs, >25% of mast cells are spindle shaped or have an atypical immature morphology[^†^](javascript:;) - *KIT* D816V mutation or other activating *KIT* mutation detected in bone marrow, peripheral blood, or other extracutaneous organs[*](javascript:;)^,^[^‡^](javascript:;) - Mast cells in bone marrow, peripheral blood or other extracutaneous organs express CD25, CD2 and/or CD30, in addition to mast cell markers - Elevated serum tryptase level, persistently  >20 ng/mL. In cases of  SM-AMN an elevated tryptase does not count as a SM minor criterion | Minor criteria   - In biopsy sections of bone marrow or other extracutaneous organs, >25% of the mast cells in the infiltrate are spindle-shaped or have atypical morphology or >25% of all mast cells in bone marrow aspirate smears are immature or atypical - Detection of an activating point mutation at codon 816 of *KIT* in the bone marrow, blood or another extracutaneous organ - Mast cells in bone marrow, blood or other extracutaneous organs express CD25, with or without CD2, in addition to normal mast cell markers^a^ - Serum total tryptase is persistently >20 ng/mL, unless there is an associated myeloid neoplasm, in which case this parameter is not valid | Minor criteria   - >25% of all mast cells are atypical cells (type I or type II) on bone marrow smears or are spindle-shaped in dense and diffuse mast cell infiltrates in sections of bone marrow or other extracutaneous organ(s)^b^ - Activating *KIT* point mutation(s) at codon 816 or in other critical regions of*KIT*^c^ in bone marrow or another extracutaneous organ(s) - Mast cells in bone marrow, blood or other extracutaneous organ(s) aberrantly express one or more of the following antigens: CD25, CD2, CD30^d^ - Baseline serum tryptase concentration >20 ng/mL in the absence of a myeloid associated haematological neoplasm.^e^ In the case of a known HαT, the tryptase level could be adjusted^f^ |
| *The diagnosis of SM can be made when the major criterion and at least 1 minor*  *criterion are present, or when ≥3 minor criteria are present*  Updated since WHO 2017 (terminology/wording aligned between ICC 2022 and WHO 2022)  Updated since WHO 2017 (terminology/wording different between ICC 2022 and WHO 2022)  Minimal or no changes since WHO 2017  **Key** | | |

HαT, hereditary alpha-tryptasaemia; M/LN-eo, myeloid/lymphoid neoplasm with eosinophilia;
SM-AMN, systemic mastocytosis with an associated myeloid neoplasm.

* In the absence of a *KIT* mutation particularly in cases with eosinophilia, the presence of tyrosine kinase gene fusions associated with M/LN-eo must be excluded.

^†^Round-cell well-differentiated morphology can occur in a small subset of cases. In these cases, the mast cells are often negative for CD25 and CD2 but positive for CD30.

^‡^To avoid ‘false-negative’ results, use of a high sensitivity PCR assay for detection of *KIT* D816V mutation is recommended. If negative, exclusion of *KIT* mutation variants is strongly recommended in suspected SM.

^a^CD25 is the more sensitive marker, by both flow cytometry and immunohistochemistry.

^b^In tissue sections, an abnormal mast cell morphology counts in both a dense infiltrate and a diffuse mast cell infiltrate. In the bone marrow smear, an atypical morphology of mast cells does not count as an SM criterion when mast cells are located in or adjacent to bone marrow particles. Morphological criteria of atypical mast cells have been described previously by Valent P, et al., 2001.

^c^Any type of *KIT* mutation counts as a minor SM criterion when published solid evidence for its transforming behaviour is available.

^d^Expression has to be confirmed by either flow cytometry or by immunohistochemistry.

^e^Myeloid neoplasms can lead to increased serum tryptase levels. Therefore, this criterion does not count in cases of systemic mastocytosis with an associated haematological neoplasm.

^f^A possible mode for adjustment has been proposed in Valent P, et al., 2021. The basal tryptase level may be divided by 1 plus the extra copy numbers of the alpha tryptase gene. For example, when the tryptase level is 30 and 2 extra copies of the alpha tryptase gene are found in a patient with HαT, the HαT-corrected tryptase level is 10 (30/3 = 10) and thus, it is not a minor SM criterion.

**ICC 2022 changes to systemic mastocytosis diagnostic criteria**

- Demonstration of tryptase and/or *KIT* (CD117) immunoreactivity has been added to the major criterion to ensure proper identification of mast cells
- The presence of any activating *KIT* mutation has been accepted as a minor criterion
- Expression of CD30 has been accepted as a minor criterion
- Identification of one of the tyrosine kinase gene fusions associated with myeloid/lymphoid neoplasm with eosinophilia excludes a diagnosis of SM
- Diagnostic criteria for SM-AMN have been included (Table 11)

**WHO 2022 changes to systemic mastocytosis diagnostic criteria**

- The presence of any activating *KIT* mutation has been accepted as a minor criterion
- Expression of CD30 has been accepted as a minor criterion
- Additional wording relating to hereditary tryptasaemia has been included in the fourth minor criterion

Table 11. Diagnostic criteria for SM-AMN as defined by the ICC 2022 classification

| **ICC 2022 SM-AMN diagnostic criteria** |
| --- |
| 1. Meets the diagnostic criteria for SM |
| 2. Meets the criteria for an associated myeloid neoplasm (e.g. CMML or other MDS/MPN, MDS, MPN, AML or other myeloid neoplasm)[*](javascript:;) |
| 3. The associated myeloid neoplasm should be fully classified according to established criteria[^†^](javascript:;) |

AML, acute myeloid leukaemia; AMN, associated myeloid neoplasm; CMML, chronic myelomonocytic leukaemia; LDH, lactate dehydrogenase; MDS, myelodysplastic neoplasms; MDS/MPN, myelodysplastic/myeloproliferative neoplasms; M/LN-eo, myeloid/lymphoid neoplasm with eosinophilia; MPN, myeloproliferative neoplasms; SM, systemic mastocytosis.

*High degree of suspicion can be raised by the presence of monocytosis, eosinophilia, splenomegaly, elevated LDH, high *KIT* D816V variant allele frequency and additional somatic mutations in genes associated with myeloid malignancies (particularly if occurring in combination) as they could be signs of an AMN.

^†^If eosinophilia is present, the presence of tyrosine kinase gene fusions associated with M/LN-eo should be excluded. Although usually mutually exclusive, rare cases with both a *KIT* mutation and a gene fusion associated with M/LN-eo have been reported. In these rare instances, the M/LN-eo would represent the SM-associated AMN, but it is recommended assigning such cases only in instances in which both a *KIT* mutation and an M/LN-eo gene fusion are present.

## 3.4 B and C findings

The WHO 2017 blue book included specific diagnostic criteria for each variant of systemic mastocytosis. Within these criteria were B ('burden of disease’) and C ('cytoreduction-requiring') findings, which indicated organ involvement without and with organ dysfunction, respectively. The B findings have been refined in both the WHO 2022 and ICC classifications. The WHO 2022 classification has also updated the C findings whereas in the ICC 2022 classification, the C findings remain unchanged from the WHO 2017 blue blook (Supplementary Table 3).

# 4. Myeloid/lymphoid neoplasms with eosinophilia and tyrosine kinase gene fusions

Since the WHO 2017 blue book, the name of this neoplasm group has changed in both the ICC 2022 and WHO 2022 classifications from myeloid/lymphoid neoplasm with eosinophilia and gene rearrangement to myeloid/lymphoid neoplasms with eosinophilia and tyrosine kinase gene fusions to specify the molecular genetic changes underlying these hematopoietic neoplasms. These *BCR::ABL1*-negative diseases have long been recognised in view of their distinctive clinicopathological features and sensitivity to TKI. They encompass a broad range of histological types, including MPN, MDS, MDS/MPN, AML and mixed phenotype acute leukaemia, as well as B- or T- lymphoblastic leukaemia/lymphoma.

## 4.1 Categorisation

Myeloid/lymphoid neoplasms with eosinophilia and tyrosine kinase gene fusion entities from the WHO 2017 blue book are included within the WHO 2022 and ICC 2022 classifications but new types have also been identified and added to the 2022 classification documents. The categories and differences between each document are listed in Table 12.

Table 12. Myeloid/lymphoid neoplasms with eosinophilia and tyrosine kinase gene fusion entities as defined by the WHO 2017 blue book, ICC 2022 and WHO 2022 classifications

| **ICC 2022** | **WHO 2017** | **WHO 2022** |
| --- | --- | --- |
| Myeloid/lymphoid neoplasm with *PDGFRA* rearrangement | Myeloid/lymphoid neoplasms with *PDGFRA* rearrangement | Myeloid/lymphoid neoplasms with *PDGFRA* rearrangement |
| Myeloid/lymphoid neoplasm with *PDGFRB* rearrangement | Myeloid/lymphoid neoplasms with *PDGFRB* rearrangement | Myeloid/lymphoid neoplasms with *PDGFRB* rearrangement |
| Myeloid/lymphoid neoplasm with *FGFR1* rearrangement | Myeloid/lymphoid neoplasms with *FGFR1* rearrangement | Myeloid/lymphoid neoplasms with *FGFR1* rearrangement |
| Myeloid/lymphoid neoplasm with *JAK2* rearrangement | *Myeloid/lymphoid neoplasms with PCM1-JAK2* | Myeloid/lymphoid neoplasms with *JAK2* rearrangement |
| Myeloid/lymphoid neoplasm with *FLT3* rearrangement |  | Myeloid/lymphoid neoplasms with *FLT3* rearrangement |
| Myeloid/lymphoid neoplasm with *ETV6::ABL1* |  | Myeloid/lymphoid neoplasms with *ETV6::ABL1* fusion |
|  |  | Myeloid/lymphoid neoplasms with other defined tyrosine kinase fusions:   - *ETV6::FGFR2* - *ETV6::LYN* - *ETV6::NTRK3* - *RANBP2::ALK* - *BCR::RET* - *FGFR1OP::RET* |

Italic font indicates provisional tumour entities.

Updated since WHO 2017 (terminology/wording aligned between ICC 2022 and WHO 2022)

Updated since WHO 2017 (terminology/wording different between ICC 2022 and WHO 2022)

Minimal or no changes since WHO 2017

**Key**

**ICC 2022 changes to myeloid/lymphoid neoplasms with eosinophilia and tyrosine kinase gene fusion entities**

- The myeloid/lymphoid neoplasms with *PCM1-JAK2* entity has been updated to myeloid/lymphoid neoplasm with *JAK2* rearrangement
- Two new entities have been added – myeloid/lymphoid neoplasm with *FLT3* rearrangement and myeloid/lymphoid neoplasm with *ETV6::ABL1*

**WHO 2022 changes to myeloid/lymphoid neoplasms with eosinophilia and tyrosine kinase gene fusion entities**

- The myeloid/lymphoid neoplasms with *PCM1-JAK2* entity has been updated to myeloid/lymphoid neoplasm with *JAK2* rearrangement
- Myeloid/lymphoid neoplasm with *FLT3* rearrangement and myeloid/lymphoid neoplasm with *ETV6::ABL1* have been added as new entities
- New scalable genetic framework introduced under a new category – myeloid/lymphoid neoplasms with other defined tyrosine kinase fusions

Further details on specific genetic abnormalities, clinical presentations and targeted therapy of myeloid/lymphoid neoplasms with eosinophilia and tyrosine kinase gene fusions are provided in each 2022 classification document.

# 5. Myelodysplastic/myeloproliferative neoplasms (MDS/MPN)

MDS/MPN are a heterogeneous group of diseases defined by overlapping pathological and molecular features of MDS and MPN, often manifesting clinically with various combinations of cytopenias and cytoses.

## 5.1 Categorisation

Since the WHO 2017 blue book, the MDS/MPN entities have been updated in both the ICC 2022 and WHO 2022 classifications. The differences between each document are listed in Table 13.

Table 13. MDS/MPN entities as defined by the WHO 2017 blue book, ICC 2022 and WHO 2022 classifications

| **ICC 2022** | **WHO 2017** | **WHO 2022** |
| --- | --- | --- |
| Chronic myelomonocytic leukaemia (CMML) | Chronic myelomonocytic leukaemia | Chronic myelomonocytic leukaemia |
| Clonal monocytosis of undetermined significance (CMUS) |  |  |
| Clonal cytopenia with monocytosis of undetermined significance (CCMUS) |  |  |
| Atypical chronic myeloid leukaemia (aCML) | Atypical chronic myeloid leukaemia, *BCR-ABL1*-negative | MDS/MPN with neutrophilia |
| (Categorised under paediatric and/or germline mutation-associated disorders) | Juvenile myelomonocytic leukaemia (JMML) | (Categorised under MPN) |
| MDS/MPN with *SF3B1* mutation and thrombocytosis | MDS/MPN with ring sideroblasts and thrombocytosis* | MDS/MPN with *SF3B1* mutation and thrombocytosis |
| MDS/MPN with ring sideroblasts and thrombocytosis, NOS^†^ |  |  |
| MDS/MPN, NOS | MDS/MPN, unclassifiable | MDS/MPN, NOS |

Updated since WHO 2017 (terminology/wording aligned between ICC 2022 and WHO 2022)

Updated since WHO 2017 (terminology/wording different between ICC 2022 and WHO 2022)

Minimal or no changes since WHO 2017

**Key**

*Retained as an acceptable term in the WHO 2022 classification to be used for cases with wild-type SF3B1 and ≥15% ring sideroblasts.

^†^Considered equivalent to MDS/MPN with *SF3B1* mutation and thrombocytosis. The WHO 2022 classification states that “in the absence of *SF3B1* mutation, a diagnosis of MDS/MPN-SF3B1-T can still be rendered provided the presence of ≥15% of ring sideroblasts.”

**ICC 2022 changes to MDS/MPN entities**

- Three new entities have been added – clonal cytopenia with monocytosis of undetermined significance; clonal monocytosis of undetermined significance and myelodysplastic/myeloproliferative neoplasm with ring sideroblasts and thrombocytosis, not otherwise specified
- The notation ‘*BCR::ABL1*-negative’ has been dropped from the name aCML
- JMML entity has been moved and is now grouped with paediatric and/or germline mutation-associated disorders
- MDS/MPN with ring sideroblasts and thrombocytosis redefined based on *SF3B1* mutation and renamed to MDS/MPN with *SF3B1* mutation and thrombocytosis
- The ‘unclassifiable’ qualifier has been updated to ‘not otherwise specified’

**WHO 2022 changes to MDS/MPN entities**

- Atypical chronic myeloid leukaemia, *BCR-ABL1*-negative renamed MDS/MPN with neutrophilia
- JMML has moved and is now classified under myeloproliferative neoplasms
- MDS/MPN with ring sideroblasts and thrombocytosis redefined based on *SF3B1* mutation and renamed to MDS/MPN with SF3B1 mutation and thrombocytosis
- The ‘unclassifiable’ qualifier has been updated to ‘not otherwise specified’

## 5.2 Diagnostic criteria

The WHO 2022 and ICC 2022 classifications have also refined diagnostic criteria for each MDS/MPN entity since the WHO 2017 blue book was published.

### 5.2.1 Chronic myelomonocytic leukaemia (CMML)

CMML is the most common MDS/MPN and is characterised by sustained peripheral blood monocytosis and various combinations of somatic mutations involving epigenetic regulation, spliceosome and signal transduction genes.

The CMML diagnostic criteria have been updated in both the ICC 2022 and WHO 2022 classifications (Table 14).

Table 14. Diagnostic criteria for CMML as defined by the WHO 2017 blue book, ICC 2022 and WHO 2022 classifications

| **ICC 2022** | **WHO 2017** | **WHO 2022** |
| --- | --- | --- |
| - Monocytosis defined as monocytes ≥0.5 × 10^9^/L and ≥10% of the WBC - Cytopenia:  anaemia, haemoglobin  <13 g/dL (males),  <12 g/dL (females); neutropenia,  ANC <1.8 × 10^9^/L; thrombocytopenia, platelets <150 × 10^9^/L[*](javascript:;) - Bone marrow examination with morphological findings consistent with CMML (hypercellularity due to a myeloid proliferation often with increased monocytes), and lacking diagnostic features of acute myeloid leukaemia, MPN or other conditions associated with monocytosis^†^ - No BCR::ABL1 or genetic abnormalities of myeloid/lymphoid neoplasms with eosinophilia and tyrosine kinase gene fusions - Blasts (including promonocytes) <20% of the cells in blood and bone marrow - Presence of clonality: abnormal cytogenetics and/or presence of at least one myeloid neoplasm associated mutation of at least 10% allele frequency^‡^ - In cases without evidence of clonality,   - monocytes ≥1.0 × 10^9^/L and >10% of the WBC, and  - increased blasts (including promonocytes),^§^ or morphological dysplasia, or  - an abnormal immunophenotype consistent with CMML would be required for its diagnosis | - Persistent peripheral blood monocytosis (≥1 × 10^9^/L) with monocytes accounting for ≥10% of the leukocytes - WHO criteria for *BCR-ABL1*-positive CML, PMF, PV and ET^a^ are not met - No rearrangement of *PDGFRA*, *PDGFRB* or *FGFR1* and no *PCM1-JAK2* (which should be specifically excluded in cases   with eosinophilia)   - Blasts^b^ constitute <20% of the cells in the peripheral blood and bone marrow - Dysplasia involving ≥1 myeloid lineages   or  If myelodysplasia is absent or minimal, criteria 1–4 are met and:  - an acquired, clonal cytogenetic or molecular genetic abnormality is present in haematopoietic cells^c^  *or*  - the monocytosis has persisted for ≥3 months and all other causes of monocytosis (e.g. malignancy, infection and inflammation) have been excluded | **Prerequisite criteria**   - Persistent absolute (≥0.5 × 10^9^/L) and relative (≥10%) peripheral blood monocytosis - Not meeting diagnostic criteria of CML or other MPN^a^ - Not meeting diagnostic criteria of myeloid/lymphoid neoplasms with tyrosine kinase fusions^d^ - Blasts constitute <20% of the cells in the peripheral blood and bone marrow^b^   **Supporting criteria**   1. Dysplasia involving ≥1 myeloid lineages^e^ 2. Acquired clonal cytogenetic or molecular abnormality 3. Abnormal partitioning of peripheral blood monocyte subsets^f^   ***Requirements for diagnosis***   - *Pre-requisite criteria must be present in all cases* - *If monocytosis is  ≥1 × 10^9^/ L: one or more supporting criteria must be met* - *If monocytosis is  <1 × 10^9^/ L: supporting criteria 1 and 2 must be met* |

Updated since WHO 2017 (terminology/wording aligned between ICC 2022 and WHO 2022)

Updated since WHO 2017 (terminology/wording different between ICC 2022 and WHO 2022)

Minimal or no changes since WHO 2017

**Key**

CCMUS, clonal cytopenia with monocytosis of undetermined significance; CML, chronic myeloid leukaemia; CMML, chronic myelomonocytic leukaemia; CMUS, clonal monocytosis of undetermined significance; ET, essential thrombocythaemia; MPN, myeloproliferative neoplasms; PMF, primary myelofibrosis; PV, polycythaemia vera; WBC, white blood cell count.

*A small proportion of cases may show only borderline or no cytopenia usually in early phase disease.

^†^For cases lacking bone marrow findings of CMML, a diagnosis of CMUS could be considered. If cytopenia is present, a diagnosis of CCMUS could be entertained. In these diagnostic settings, however, an alternative cause for the observed monocytosis would have to be excluded based on appropriate clinicopathological correlations.

^‡^Based on International Consensus Group Conference, Vienna, 2018.

^§^Increased blasts: ≥5% in the bone marrow and/or ≥2% in the peripheral blood.

^a^MPN can be associated with monocytosis or it can develop during the course of the disease; such cases can mimic CMML. In these rare instances, a documented history of MPN excludes CMML, whereas the presence of MPN features in the bone marrow and/or MPN-associated mutations (in *JAK2*, *CALR* or *MPL*) tends to support MPN with monocytosis rather than CMML.

^b^Blasts and blast equivalents include myeloblasts, monoblasts and promonocytes. Promonocytes are monocytic precursors with abundant light-grey or slightly basophilic cytoplasm with a few scattered fine lilac-coloured granules, finely distributed stippled nuclear chromatin, variably prominent nucleoli and delicate nuclear folding or creasing. Abnormal monocytes, which can be present in both the peripheral blood and the bone marrow, are excluded from the blast count.

^c^In the appropriate clinical context, mutations in genes often associated with CMML (e.g. *TET2*, *SRSF2*, *ASXL1* and *SETBP1*) support the diagnosis. However, some of these mutations can be age-related or present in other neoplasms; therefore, these genetic findings must be interpreted with caution.

^d^Criteria for myeloid/lymphoid neoplasms with tyrosine kinase fusions should be specifically excluded in cases with eosinophilia.

^e^Morphological dysplasia should be present in ≥10% of cells of a haematopoietic lineage in the bone marrow.

^f^Based on detection of increased classical monocytes (>94%) in the absence of known active autoimmune diseases and/or systemic inflammatory syndromes.

**ICC 2022 changes to CMML diagnostic criteria**

- The definition of monocytosis has changed
- A criterion relating to cytopenia has been added
- The criterion relating to dysplasia has been replaced by two new criteria relating to clonality
- The criterion relating to MPN or other conditions associated with monocytosis has been expanded
- The criterion relating to gene rearrangement has been reworded

**WHO 2022 changes to CMML diagnostic criteria**

- Diagnostic criteria have been restructured into pre-requisite and supporting criteria with additional guidance on requirements for diagnosis
- The criteria relating to gene rearrangements has been reworded
- The criterion relating to dysplasia has moved from a pre-requisite criterion to a supporting criterion
- Two additional supporting criteria have been added

CMML subtyping

Both the ICC 2022 and WHO 2022 classifications formally recognise two subtypes of CMML, based on white blood cell count:

- Myelodysplastic CMML (MD-CMML): WBC <13 × 10^9^/L
- Myeloproliferative CMML (MP-CMML): WBC ≥13 × 10^9^/L

CMML subgrouping

In the previous WHO 2017 blue book, three subgroups of CMML were defined by the percentage of blasts and promonocytes in the peripheral blood and bone marrow. These subgroups and their criteria have since been updated in both the ICC 2022 and WHO 2022 classifications (Table 15).

Table 15. CMML subgroups as defined by the WHO 2017 blue book, ICC 2022 and WHO 2022 classifications

| **ICC 2022** | **WHO 2017** | **WHO 2022** |
| --- | --- | --- |
| (CMML-0 has been eliminated in view of evidence that it provides no or limited prognostic significance) | **CMML-0:** <2% blasts in the blood and <5% in the bone marrow; no Auer rods | (CMML-0 has been eliminated in view of evidence that it provides no or limited prognostic significance) |
| **CMML-1:** <5% blasts in peripheral blood and <10% in bone marrow | **CMML-1:** 2–4% blasts in the blood or 5–9% in the bone marrow; <5% blasts in the blood, <10% blasts in the bone marrow, and no Auer rods | **CMML-1:** <5% blasts in peripheral blood, <10% in bone marrow |
| **CMML-2:** 5–19% blasts in peripheral blood and 10–19% in bone marrow | **CMML-2:** 5–19% blasts in the blood, 10–19% in the bone marrow or Auer rods are present; <20% blasts in the bone marrow and blood | **CMML-2:** 5–19% blasts in peripheral blood, 10–19% in bone marrow, or Auer rods |

Updated since WHO 2017 (terminology/wording aligned between ICC 2022 and WHO 2022)

Updated since WHO 2017 (terminology/wording different between ICC 2022 and WHO 2022)

Minimal or no changes since WHO 2017

**Key**

### 5.2.2 Clonal monocytosis of undetermined significance (CMUS) and clonal cytopenia with monocytosis of undetermined significance (CCMUS)

CMUS and CCMUS are new entities of MDS/MPN in the ICC 2022 classification which are not included in the WHO 2017 blue book or the WHO 2022 classification. The ICC recognises CMUS as a precursor condition of CMML. It is characterised by persistent monocytosis (monocytes ≥10% and ≥0.5 × 10^9^/L of the white blood cell count), in the presence of myeloid neoplasm-associated mutation(s) without bone marrow morphological findings of CMML. If cytopenia is present, the nomenclature of clonal cytopenia and monocytosis of undetermined significance (CCMUS) is suggested. Table 16 describes the diagnostic criteria for these two new entities.

Table 16. Diagnostic criteria for CMUS and CCMUS as defined by the ICC 2022 classification

| **ICC 2022 CMUS and CCMUS diagnostic criteria** |
| --- |
| Persistent monocytosis defined as monocytes ≥0.5 × 10^9^/L and ≥10% of the WBC |
| Absence or presence of cytopenia (thresholds same as for MDS)[*](javascript:;) |
| Presence of at least one myeloid neoplasm associated mutation of appropriate allele frequency (i.e. ≥2%)[^†^](javascript:;) |
| No significant dysplasia, increased blasts (including promonocytes) or morphological findings of CMML on bone marrow examination[^‡^](javascript:;) |
| No criteria for a myeloid or other haematopoietic neoplasm are fulfilled |
| No reactive condition that would explain a monocytosis is detected |

*If cytopenia is present the nomenclature of CCMUS is suggested.

^†^VAF threshold based on International Consensus Group Conference, Vienna, 2018.

^‡^Bone marrow findings of CMML include hypercellularity with myeloid predominance, often with increased monocytes and in a proportion of cases monoblasts and/or blast equivalents (i.e. promonocytes) and/or dysplasia in at least one lineage.

### 5.2.3 Atypical chronic myeloid leukaemia (aCML) or MDS/MPN with neutrophilia

aCML is a *BCR::ABL1*-negative, leukaemic disorder with myelodysplastic as well as myeloproliferative features present at the time of initial diagnosis. It is characterised by principal involvement of the neutrophil lineage, with leukocytosis resulting from an increase of morphologically dysplastic neutrophils and their precursors. However, multilineage dysplasia is common, and reflects the stem cell origin of this entity.

Because the absence of *BCR*::*ABL1* is a requirement for diagnosing all MDS/MPN entities, the ICC 2022 classification has dropped the notation ‘*BCR*::*ABL1* negative’ from the name aCML. In contrast, the WHO 2022 classification has renamed the aCML entity ‘MDS/MPN with neutrophilia’ to underscore the nature of the disease and avoid potential confusion with CML.

The WHO 2017 blue book provided diagnostic criteria for aCML that remain unchanged in the WHO 2022 classification. However, these criteria have been updated in the ICC 2022 classification (Table 17).

Table 17. Diagnostic criteria for aCML (or MDS/MPN with neutrophilia) as defined by the WHO 2017 blue book, ICC 2022 and WHO 2022 classifications

| **ICC 2022** | **WHO 2017** | **WHO 2022** |
| --- | --- | --- |
| - Leukocytosis ≥13 × 10^9^/L, due to increased numbers of neutrophils and their precursors (promyelocytes, myelocytes and metamyelocytes), the latter constituting ≥10% of the leukocytes - Cytopenia:  anaemia, haemoglobin  <13 g/dL (males),  <12 g/dL (females); neutropenia,  ANC <1.8 × 10^9^/L; thrombocytopenia, platelets <150 × 10^9^/L[*](javascript:;) - Dysgranulopoiesis, including the presence of abnormal hyposegmented and/or hypersegmented neutrophils ± abnormal chromatin clumping      - No eosinophilia; eosinophils constitute <10% of the peripheral blood leukocytes      - No or minimal absolute monocytosis; monocytes constitute <10% of the peripheral blood leukocytes - Hypercellular bone marrow with granulocytic proliferation and granulocytic dysplasia, with or without dysplasia in the erythroid and megakaryocytic lineages - Blasts <20% of the cells in blood and bone marrow - No *BCR::ABL1* or genetic abnormalities of myeloid/lymphoid neoplasms with eosinophilia and tyrosine kinase gene fusions. The absence of MPN-associated driver mutations and the presence of *SETBP1* mutations in association with *ASXL1* provide additional support for a diagnosis of aCML | - Peripheral blood leukocytosis ≥13 × 10^9^/L, due to increased numbers of neutrophils and their precursors (i.e. promyelocytes, myelocytes and metamyelocytes), with neutrophil precursors constituting ≥10% of the leukocytes - Dysgranulopoiesis, which may include abnormal chromatin clumping - No or minimal absolute basophilia; basophils constitute <2% of the peripheral blood leukocytes - No or minimal absolute monocytosis; monocytes constitute <10% of the peripheral blood leukocytes - Hypercellular bone marrow with granulocytic proliferation and granulocytic dysplasia,   with or without dysplasia in the erythroid and megakaryocytic lineages   - <20% blasts in the blood and bone marrow      - WHO criteria for *BCR-ABL1*-positive CML, PMF, PV or ET^a^ are not met - No evidence of *PDGFRA*, *PDGFRB* or *FGFR1* rearrangement, or of *PCM1-JAK2* | **Essential criteria**   - Peripheral blood leukocytosis ≥13 × 10^9^/L, with neutrophilia and ≥10% circulating immature myeloid cells (promyelocytes, myelocytes and metamyelocytes), as well as neutrophilic dysplasia - Hypercellular bone marrow with granulocytic predominance and granulocytic dysplasia, with or without dysplasia in the erythroid and megakaryocytic lineages - <20% blasts in the blood and bone marrow - Not meeting diagnostic criteria for MPN (specifically, exclusion of *BCR::ABL1* fusion)^b^, myeloid neoplasms with eosinophilia and defining gene rearrangement, CMML or MDS/MPN with *SF3B1* mutation and thrombocytosis   **Desirable criteria**   - Detection of *SETBP1* and/or *ETNK1*  mutations - Absence of mutations in *JAK2*, *CALR*, *MPL*, and *CSF3R*^c^ |

Updated since WHO 2017 (terminology/wording aligned between ICC 2022 and WHO 2022)

Updated since WHO 2017 (terminology/wording different between ICC 2022 and WHO 2022)

Minimal or no changes since WHO 2017

**Key**

aCML, atypical leukaemia; ANC, absolute neutrophil count; CML, chronic myeloid leukaemia; CMML, chronic myelomonocytic leukaemia; CNL, chronic neutrophilic leukaemia; ET, essential thrombocythaemia; MDS/MPN, myelodysplastic/myeloproliferative neoplasm; MPN, myeloproliferative neoplasm; PCR, polymerase chain reaction; PMF, primary myelofibrosis;
PV, polycythaemia vera.

^a^MPN, in particular those in accelerated phase and/or in post-PV or post-ET MF, if neutrophilic, may simulate aCML. A history of MPN, the presence of MPN features in the bone marrow, and/or MPN-associated mutations (in *JAK2*, *CALR* or *MPL*) tend to exclude the diagnosis of aCML; conversely, the diagnosis is supported by the presence of *SETBP1* and/or *ETNK1* mutations. *CSF3R* mutation is uncommon and, if detected, should prompt careful morphological review to exclude an alternative diagnosis of CNL or another myeloid neoplasm.

^b^The diagnosis of MDS/MPN with neutrophilia requires exclusion of *BCR::ABL1* fusion, which requires careful evaluation to exclude cryptic rearrangements and/or alternate *BCR::ABL1* transcripts by available methodologies (e.g. cytogenetics, in situ hybridisation or PCR-based assays).

^c^Mutations in these genes are uncommon in MDS/MPN with neutrophilia and should prompt morphological review to exclude alternative diagnoses.

**ICC 2022 changes to aCML diagnostic criteria**

- A criterion relating to cytopenia has been added
- The criterion relating to dysgranulopoiesis has been expanded
- The criterion relating to basophilia has been replaced with one relating to eosinophilia
- The criteria relating to MPN exclusion and genetic abnormalities and have been combined into one updated criterion

**WHO 2022 changes to aCML (or MDS/MPN with neutrophilia) diagnostic criteria**

- Diagnostic criteria have been restructured into essential and desirable criteria
- The criterion relating to leukocytosis has been updated to include neutrophilic dysplasia
- The criteria relating to dysgranulopoiesis, basophilia and monocytosis have been omitted
- The criterion relating to exclusion of MPN has been updated
- Two desirable criteria relating to gene mutations have been added

### 5.2.4 Juvenile myelomonocytic leukaemia (JMML)

JMML is a clonal haematopoietic disorder of childhood characterised by a proliferation principally of the granulocytic and monocytic lineages. The pathogenetic mechanism in at least 90% of cases involves unchecked activation of the RAS pathway.

The ICC 2022 classification moved JMML to be grouped with paediatric and/or germline mutation-associated disorders as this disease typically presents in early childhood. The WHO 2022 classification moved JMML to be grouped with MPN due to its molecular pathogenesis and the virtual absence of stigmata of bona fide myelodysplastic neoplasia in this disease.

The JMML diagnostic criteria have been updated in both the ICC 2022 and WHO 2022 classifications (Table 18).

Table 18. Diagnostic criteria for JMML as defined by the WHO 2017 blue book, ICC 2022 and WHO 2022 classifications

| **ICC 2022** | **WHO 2017** | **WHO 2022** |
| --- | --- | --- |
| Clinical and haematological features (the first two features are present in most cases; the last two are required)   - Peripheral blood monocyte count ≥1 × 10^9^/L - Splenomegaly - Blast percentage in Peripheral blood and bone marrow <20% - Absence of *BCR::ABL1* | Clinical and haematological criteria (all four criteria are required)   - Peripheral blood monocyte count ≥1 × 10^9^/L - Splenomegaly - Blast percentage in peripheral blood and bone marrow of <20% - No Ph chromosome or  *BCR-ABL1* fusion | Clinical, haematological and laboratory criteria (all five criteria are required)   - Peripheral blood monocyte count ≥1 × 10^9^/L - Clinical evidence of organ infiltration, most commonly splenomegaly - Blast and promonocyte percentage in peripheral blood and bone marrow of <20% - No Ph chromosome or  *BCR-ABL1* fusion - No *KMT2A* (*MLL1*) gene rearrangement |
| Genetic studies (one finding required)   - Somatic mutation in *PTPN11*^‡^, *KRAS*^‡^, *NRAS*^‡^ or *RRAS*^‡^ - Germline*NF1* mutation and loss of heterozygosity of *NF1* or clinical diagnosis of neurofibromatosis type 1 - Germline *CBL* mutation and loss of heterozygosity of CBL[^§^](javascript:;) | Genetic criteria (any one criterion is sufficient)   - Somatic mutation^a^ in *PTPN11*, *KRAS* or *NRAS* - Clinical diagnosis of neurofibromatosis type 1 or *NF1* mutation - Germline *CBL* mutation and loss of heterozygosity of *CBL*[^§^](javascript:;) | Genetic criteria (any one criterion is sufficient)   - Mutation in a component or a regulator of the canonical RAS pathway: - Clonal somatic mutation in *PTPN11*, *KRAS* or *NRAS*^b^ - Clonal somatic or germline *NF1* mutation and loss of heterozygosity or compound heterozygosity of *NF1* - Clonal somatic or germline *CBL* mutation and loss of heterozygosity of *CBL*[^§^](javascript:;) - Non-canonical clonal RAS pathway pathogenic variant^c^ or fusions causing activation of genes upstream of the RAS pathway, such as *ALK*, *PDGFR-B*, *ROS1*, among others |
|  | Other criteria  Cases that do not meet any of the genetic criteria above must meet the following criteria in addition to the clinical and haematological criteria above:   - Monosomy 7 or any other chromosomal abnormality   or   - ≥2 of the following: - Increased haemoglobin F for age - Myeloid or erythroid precursors on peripheral blood smear - GM-CSF hypersensitivity in colony assay - Hyperphosphorylation of STAT5 | Other criteria  Cases that do not meet any of the genetic criteria above (or in conditions where genetic testing is not available) must meet the following criteria in addition to the clinical, haematological and laboratory criteria above:   - ≥2 of the following: - Increased haemoglobin F for age - Myeloid or erythroid precursors on peripheral blood smear - Thrombocytopenia with hypercellular marrow often showing decreased number of megakaryocytes. Dysplastic features may or may not be evident. - Hypersensitivity of myeloid progenitors to GM-CSF as tested in clonogenic assays in methylcellulose or by measuring STAT5 phosphorylation in the absence or with low dose of exogenous GM-CSF |

Updated since WHO 2017 (terminology/wording aligned between ICC 2022 and WHO 2022)

Updated since WHO 2017 (terminology/wording different between ICC 2022 and WHO 2022)

Minimal or no changes since WHO 2017

**Key**

GM-CSF, granulocyte-macrophage colony-stimulating factor; JMML, juvenile myelomonocytic leukaemia; RAS, rat sarcoma.

*This monocyte threshold is not reached in approximately 7% of cases.

^†^Splenomegaly is absent in 3% of cases at presentation.

^‡^Germline mutations (indicating Noonan syndrome) need to be excluded.

^§^Occasional cases with heterozygous splice site mutations.

^a^If a mutation is found in *PTPN11*, *KRAS* or *NRAS* it is essential to consider that it might be a germline mutation and the diagnosis of transient abnormal myelopoiesis of Noonan syndrome must be considered.

^b^Germline mutation in *PTPN11, KRAS, NRAS* (Noonan syndrome) may produce JMML-like transient myeloproliferative disorder.

^c^e.g. *RRAS, RRAS2*

**ICC 2022 changes to JMML diagnostic criteria**

- Only two clinical and haematological criteria (blast percentage and absence of *BCR::ABL1*) are required
- RRAS somatic mutation added to genetic criteria
- The genetic criterion relating to *NF1* mutation has been expanded to include loss of heterozygosity of *NF1*
- No ‘other criteria’ are included if cases do not meet the genetic criteria. Cases that phenotypically mimic JMML but do not have a RAS pathway mutation are classified as JMML-like neoplasms or Noonan syndrome-associated myeloproliferative disorder (Table 19)

**WHO 2022 change to JMML diagnostic criteria**

- The criteria relating to splenomegaly and blast percentage have been expanded
- Exclusion of *KMT2A* rearrangements has been added as a criterion
- The genetic criteria have been updated to include non-canonical clonal RAS pathway pathogenic variant or fusions causing activation of genes upstream of the RAS pathway
- Monosomy 7 excluded as a cytogenetic criterion
- Thrombocytopenia added as new ‘other’ criterion
- The ‘other criteria’ relating to GM-CSF hypersensitivity and hyperphosphorylation of STAT5 have been combined into one criterion and updated

Table 19. Diagnostic features of JMML, JMML-like neoplasms and Noonan syndrome-associated myeloproliferative disorder as defined by the ICC 2022 classification

| **ICC diagnostic features** | | | | |
| --- | --- | --- | --- | --- |
|  | **PB/BM** | **Mutation** | **Secondary mutations** | **Karyotype** |
| JMML | <20% PB <20% BM | PTPN11, NRAS, KRAS, RRAS, NF1*, CBL^†^ | Any | Any (monosomy 7 in 25%) |
| JMML-like neoplasms | <20% PB <20% BM | Absence of RAS-pathway mutation | Any | Any |
| Noonan syndrome-associated myeloproliferative disorder | <20% PB <20% BM | PTPN11^‡^, NRAS^‡^, KRAS^‡^, RIT1^‡^ | None | Normal^§^ |

*Germline mutation with additional aberration resulting in biallelic inactivation of the *NF1* gene.

^†^Germline mutation with additional aberration resulting in biallelic inactivation of the *CBL* gene; some cases with heterozygous germline mutation only.

^‡^Germline mutation, patients generally display syndromic features of Noonan syndrome.

^§^In rare instances, monosomy 7 can develop.

### 5.2.5 MDS/MPN with *SF3B1* mutation and thrombocytosis

In the WHO 2017 blue book, this entity was named MDS/MPN with ring sideroblasts and thrombocytosis. However, after the discovery that this disorder is frequently associated with mutations in the spliceosome gene *SF3B1* (which in turn are associated with the presence of ring sideroblasts), both the ICC 2022 and WHO 2022 classifications renamed this disorder based on the *SF3B1* mutation. The WHO 2022 classification states that MDS/MPN with ring sideroblasts and thrombocytosis is an acceptable term to use for cases with wild-type *SF3B1* and ≥15% ring sideroblasts.

The WHO 2017 blue book provided diagnostic criteria for this entity that have been refined in the ICC 2022 and WHO 2022 classifications (Table 20). In addition, the ICC 2022 classification provides diagnostic criteria for a new entity – MDS/MPN with ring sideroblasts and thrombocytosis, not otherwise specified (MDS/MPN-RS-T, NOS) (Table 21).

Table 20. Diagnostic criteria for MDS/MPN with *SF3B1* mutation and thrombocytosis as defined by the WHO 2017 blue book, ICC 2022 and WHO 2022 classifications

| **ICC 2022** | **WHO 2017** | **WHO 2022** |
| --- | --- | --- |
| - Anaemia, haemoglobin <13 g/dL (males),  <12 g/dL (females) - Blasts <1% in blood and <5% in bone marrow - Thrombocytosis, with platelet count ≥450 × 10^9^/L - Presence of *SF3B1* mutation (VAF >10%), isolated or associated with abnormal cytogenetics and/or other myeloid neoplasm-associated mutations - No history of recent cytotoxic or growth factor therapy that could explain the myelodysplastic/ myeloproliferative features - No *BCR::ABL1* or genetic abnormalities of myeloid/lymphoid neoplasms with eosinophilia and tyrosine kinase gene fusions; no t(3;3)(q21.3;q26.2), inv(3)(q21.3q26.2) or del(5q)[*](javascript:;) - No history of MPN, MDS or other MDS/MPN | - Anaemia associated with erythroid-lineage dysplasia, with or without multilineage dysplasia; ≥15% ring sideroblasts^a^, <1% blasts in the peripheral blood and <5% blasts in the bone marrow - Persistent thrombocytosis, with platelet count >450 × 10^9^/L - *SF3B1* mutation or, in the absence of *SF3B1* mutation, no history of recent cytotoxic or growth factor therapy that could explain the myelodysplastic/ myeloproliferative features^b^ - No *BCR-ABL1* fusion; no rearrangement of *PDGFRA*, *PDGFRB* or *FGFR1*: no *PCM1-JAK2* and no t(3;3)(q21.3;q26.2), inv(3)(q21.3q26.2) or del(5q)* - No history of MPN, MDS (except myelodysplastic syndrome with ring sideroblasts), or other MDS/MPN | - Anaemia associated with dysplastic erythropoiesis and ≥15% ring sideroblasts, with or without dysplasia in the megakaryocytic and erythroid lineages - Persistent thrombocytosis, with platelet count ≥450 × 10^9^/L - *SF3B1* mutation and concurrent *JAK2* p.V617F, or, in the absence of these mutations, concurrent biologically similar mutations involving spliceosome factors and cell signalling (e.g. *MPL* or *CBL*) - Not meeting diagnostic criteria for MDS, MPN, CMML, AML with *MECOM*  rearrangement or myeloid/lymphoid neoplasms with eosinophilia |

Updated since WHO 2017 (terminology/wording aligned between ICC 2022 and WHO 2022)

Updated since WHO 2017 (terminology/wording different between ICC 2022 and WHO 2022)

Minimal or no changes since WHO 2017

**Key**

MDS, myelodysplastic syndrome; MDS/MPN, myelodysplastic/myeloproliferative neoplasm; MPN, myeloproliferative neoplasm; VAF, variant allele frequency.

*In a case that otherwise meets the diagnostic criteria for myelodysplastic syndrome with del(5q).

^a^≥15% ring sideroblasts is a required criterion even if *SF3B1* mutation is detected.

^b^The diagnosis of MDS/MPN with ring sideroblasts and thrombocytosis is strongly supported by the presence of *SF3B1* mutation together with a *JAK2* V617F, *CALR* or *MPL* mutation.

**ICC 2022 changes to MDS/MPN with *SF3B1* mutation and thrombocytosis diagnostic criteria**

- The criterion relating to anaemia has been updated
- ≥15% ring sideroblasts has been omitted as a criterion
- Blast percentage has been included as a separate criterion to anaemia
- The criterion relating to SF3B1 mutation and cytotoxic or growth factor therapy has been separated in two updated criteria
- Presence of *SF3B1* mutation is a required criterion. Rare cases of MDS/MPN with thrombocytosis and ≥15% ring sideroblasts that lack *SF3B1* mutation are reclassified as MDS/MPN with ring sideroblasts and thrombocytosis, not otherwise specified (MDS/MPN-RS-T, NOS), (Table 21)
- The criterion relating to gene rearrangements has been refined

**WHO 2022 changes to MDS/MPN with *SF3B1* mutation and thrombocytosis diagnostic criteria**

- Blast percentages have been omitted from the first criterion
- The criterion relating to SF3B1 mutation has been expanded and wording relating to cytotoxic or growth factor therapy has been omitted
- The criterion relating to gene rearrangements has been omitted
- The criterion relating to exclusion of other myeloid neoplasms has been expanded

Table 21. Diagnostic criteria for MDS/MPN with ring sideroblasts and thrombocytosis, not otherwise specified as defined by the ICC 2022 classification

| **ICC 2022 diagnostic criteria for MDS/MPN-RS-T, NOS** |
| --- |
| Thrombocytosis, with platelet count ≥450 × 10^9^/L |
| Anaemia associated with erythroid-lineage dysplasia, with or without multilineage dysplasia, and ≥15% ring sideroblasts |
| Blasts <1% in blood and <5% in bone marrow |
| Presence of clonality: demonstration of a clonal cytogenetic abnormality and/or somatic mutation(s). In their absence, no history of recent cytotoxic or growth factor therapy that could explain the myelodysplastic/myeloproliferative features |
| Absence of *SF3B1* mutation; no *BCR::ABL1* or genetic abnormalities of myeloid/lymphoid neoplasms with eosinophilia and tyrosine kinase gene fusions; no t(3;3)(q21.3;q26.2), inv(3) (q21.3q26.2), or del(5q)[*](javascript:;) |
| No history of MPN, MDS or other myelodysplastic/myeloproliferative neoplasm |

*In a case that otherwise meets the diagnostic criteria for myelodysplastic syndrome with del(5q).

### 5.2.6 MDS/MPN, NOS

This entity covers myeloid neoplasms with mixed myeloproliferative and myelodysplastic features at onset that do not meet the criteria for any other MDS/MPN, MDS or MPN. Both the ICC 2022 and WHO 2022 classifications have updated the ‘unclassifiable’ qualifier used in the WHO 2017 blue book to ‘not otherwise specified’.

The WHO 2017 blue book provided diagnostic criteria for this entity that remain unchanged in the WHO 2022 classification. The ICC have updated these criteria (Table 22).

Table 22. Diagnostic criteria for MDS/MPN, NOS as defined by the WHO 2017 blue book, ICC 2022 and WHO 2022 classifications

| **ICC 2022** | **WHO 2017** | **WHO 2022** |
| --- | --- | --- |
| - Myeloid neoplasm with mixed myeloproliferative and myelodysplastic features, not meeting the criteria for any other MDS/MPN, MDS, MPN[*](javascript:;) - Cytopenia:  anaemia, haemoglobin  <13 g/dL (males),  <12 g/dL (females); neutropenia,  ANC <1.8 × 10^9^/L; thrombocytopenia, platelets <150 × 10^9^/L - Blasts <20% of the cells in blood and bone marrow - A platelet count of ≥450 × 10^9^/L and/or a white blood cell count of ≥13 × 10^9^/L - Presence of clonality: demonstration of a clonal cytogenetic abnormality and/or somatic mutation(s). If clonality cannot be determined, the findings have persisted and all other causes (e.g. history of cytotoxic or growth factor therapy or other primary cause that could explain the myelodysplastic/ myeloproliferative features) have been excluded - No *BCR::ABL1* or genetic abnormalities of myeloid/lymphoid neoplasms with eosinophilia and tyrosine kinase gene fusions; no t(3;3)(q21.3;q26.2), inv(3)(q21.3q26.2),[^†^](javascript:;) or del(5q)[^‡^](javascript:;) | - Myeloid neoplasm with mixed myeloproliferative and myelodysplastic features at onset, not meeting the WHO criteria for any other MDS/MPN, MDS, MPN - <20% blasts in the peripheral blood and bone marrow - Clinical and morphological features of one of the categories of myelodysplastic syndrome^a^ - Clinical and morphological myeloproliferative features manifesting as a platelet count of ≥450 × 10^9^/L associated with bone marrow megakaryocytic proliferation and/or a white blood cell count of  ≥13 × 10^9^/L^a^ - No history of recent cytotoxic or growth factor therapy that could explain the myelodysplastic/ myeloproliferative features - No *PDGFRA*, *PDGFRB* or *FGFR1* rearrangement and no *PCM1-JAK2* | - Myeloid neoplasm with mixed myeloproliferative and myelodysplastic features at onset, not meeting the WHO criteria for any other MDS/MPN, MDS, MPN - <20% blasts in the peripheral blood and bone marrow - Clinical and morphological features of one of the categories of myelodysplastic syndrome^a^ - Clinical and morphological myeloproliferative features manifesting as a platelet count of ≥450 × 10^9^/L associated with bone marrow megakaryocytic proliferation and/or a white blood cell count of  ≥13 × 10^9^/L^a^ - No history of recent cytotoxic or growth factor therapy that could explain the myelodysplastic/ myeloproliferative features - No *PDGFRA*, *PDGFRB* or *FGFR1* rearrangement and no *PCM1-JAK2* |

Updated since WHO 2017 (terminology/wording aligned between ICC 2022 and WHO 2022)

Updated since WHO 2017 (terminology/wording different between ICC 2022 and WHO 2022)

Minimal or no changes since WHO 2017

**Key**

ANC, absolute neutrophil count; CEL, chronic eosinophilic leukaemia; MDS, myelodysplastic syndrome; MDS/MPN, myelodysplastic/myeloproliferative neoplasm; MPN, myeloproliferative neoplasm; NOS, not otherwise specified; VAF, variant allele frequency.

*MPN, in particular those in accelerated phase and/or in post-PV or post-ET myelofibrotic stage, may simulate MDS/MPN, NOS. A history of MPN and/or the presence of MPN-associated mutations (in *JAK2*, *CALR*, or *MPL*) particularly if associated with a high VAF, tend to exclude a diagnosis of MDS/MPN, NOS. The presence of hypereosinophilia would favour a diagnosis of CEL, NOS.

^†^In a case that otherwise meets criteria for MDS, NOS.

^‡^In a case that otherwise meets the diagnostic criteria for MDS with isolated del(5q).

^a^Cases that meet criteria for MDS with isolated del(5q) are excluded irrespective of the presence of thrombocytosis or leukocytosis.

**ICC 2022 changes to MDS/MPN, NOS diagnostic criteria**

- A criterion relating to cytopenia has been added
- The criterion relating to clinical and morphological features of MDS has been omitted
- A criterion relating to the presence of clonality has been added
- The criterion relating to gene rearrangements has been updated

**WHO 2022 changes to MDS/MPN, NOS diagnostic criteria**

- No major changes since the WHO 2017 blue book

# 6. Clonal haematopoiesis (CH)

Clonal haematopoiesis occurs when an expanded population of blood cells is derived from a single clone. The incidence of CH increases with age. Since the WHO 2017 blue book was published, there have been advances in the identification of CH (by the detection of somatic mutations or cytogenetic aberrations or copy number abnormalities on genetic testing) and an increased understanding of its implications. The WHO 2022 classification recognises CH as a category of precursor myeloid disease state.

In addition, the ICC 2022 and WHO 2022 classifications formally define the two main states of CH – clonal haematopoiesis of indeterminate potential (CHIP) and clonal cytopenia of undetermined significance (CCUS). In the ICC 2022 classification, CCUS is included under a new category (premalignant clonal cytopenias).

## 6.1 Cytopenia

**ICC 2022 and WHO 2022 definition**

In the ICC 2022 and WHO 2022 classifications, cytopenia (in the context of clonal cytopenias) is defined as the presence of acquired and sustained anaemia (haemoglobin
<13 g/dL in males, <12 g/dL in females), neutropenia, (absolute neutrophil count
<1.8 × 10^9^/L) and/or thrombocytopenia (platelets <150 × 10^9^/L[),](javascript:;) that is not explained by another condition.

## 6.2 Clonal haematopoiesis of indeterminate potential (CHIP)

**ICC 2022 definition**

- CH with the presence of a somatic mutation in a myeloid neoplasm driver gene (at variant allele fraction ≥2%) or a non-MDS-defining clonal cytogenetic aberration in a patient lacking a myeloid neoplasm or unexplained cytopenia

**WHO 2022 definition**

- CH harbouring somatic mutations of myeloid malignancy-associated genes detected in the blood or bone marrow at a variant allele fraction of ≥2% (≥4% for X-linked gene mutations in males) in individuals without a diagnosed haematological disorder or unexplained cytopenia

## 6.3 Clonal cytopenia of undetermined significance (CCUS)

**ICC 2022 definition**

- CHIP detected when the cytopenia is persistent (4 months or longer in duration), idiopathic, and not caused by another comorbid condition, which must be carefully excluded

**WHO 2022 definition**

- CHIP detected in the presence of one or more persistent cytopenias that are otherwise unexplained by haematological or non-haematological conditions and that do not meet diagnostic criteria for defined myeloid neoplasms

# 7. Myelodysplastic syndromes/neoplasms (MDS)

MDS are clonal haematopoietic neoplasms defined by cytopenia and morphological dysplasia. The threshold for defining dysplasia is recommended as 10% for all lineages.

## 7.1 Categorisation

There have been some key changes to the categorisation of MDS in both the WHO 2022 and ICC 2022 classifications since the WHO 2017 blue book (Table 23).

In addition, the WHO 2022 document has introduced the term *myelodysplastic neoplasms* (abbreviated to MDS) to replace *myelodysplastic syndromes* which is used in the WHO 2017 blue book and the ICC 2022 classification.

Table 23. MDS entities as defined by the WHO 2017 blue book, ICC 2022 and WHO 2022 classifications

| **ICC 2022** | **WHO 2017** | **WHO 2022** |
| --- | --- | --- |
|  |  | **MDS with defining genetic**  **abnormalities** |
| MDS with del(5q) | MDS with isolated del(5q) | MDS with low blasts and isolated 5q deletion |
| MDS with mutated *SF3B1* | MDS with ring sideroblasts and single lineage dysplasia | MDS with low blasts and *SF3B1* mutation |
|  | MDS with ring sideroblasts and multilineage dysplasia |  |
| MDS with mutated *TP53* |  | MDS with biallelic *TP53* inactivation |
|  |  | **MDS, morphologically defined** |
| MDS, NOS without dysplasia |  |  |
| MDS, NOS with single lineage dysplasia | MDS with single lineage dysplasia |  |
| MDS, NOS with multilineage dysplasia | MDS with multilineage dysplasia |  |
|  |  | MDS with low blasts |
|  |  | MDS, hypoplastic |
| MDS with excess blasts | MDS with excess blasts | MDS with increased blasts |
|  | - MDS-EB-1 | - MDS-IB1 |
| MDS/AML  (10–19% blasts) | - MDS-EB-2 | - MDS-IB2 |
|  |  | - MDS with fibrosis |
|  | MDS, unclassifiable |  |
|  | *Refractory cytopenia of childhood* |  |

Italic font indicates provisional tumour entities.

Updated since WHO 2017 (terminology/wording aligned between ICC 2022 and WHO 2022)

Updated since WHO 2017 (terminology/wording different between ICC 2022 and WHO 2022)

Minimal or no changes since WHO 2017

**Key**

**ICC 2022 changes to MDS entities**

- MDS with ring sideroblasts has been replaced by MDS with *SF3B1* mutation irrespective of single or multi-lineage dysplasia
- MDS with mutated *TP53* added as a new entity
- 3 entities of myelodysplastic syndrome, not otherwise specified (MDS, NOS) defined:
  - MDS, NOS without dysplasia (new term)
  - MDS, NOS with single lineage dysplasia
  - MDS, NOS with multilineage dysplasia
- MDS/AML introduced as a new entity to replace the MDS-EB-2 subentity of MDS with excess blasts
- MDS, unclassifiable entity has been eliminated
- Refractory cytopenia of childhood has been moved to a new section of paediatric disorders

**WHO 2022 changes to MDS entities**

- MDS entities are now grouped as those having defining genetic abnormalities and those that are morphologically defined:
  - MDS genetic types include: MDS with low blasts and *SF3B1* mutation, MDS with low blasts and isolated 5q deletion and MDS with biallelic *TP53* inactivation
  - Morphologically defined MDS types include: MDS with low blasts (new term), hypoplastic MDS (now recognised as a distinct disease type) and MDS with increased blasts (new term)
- MDS with increased blasts separated into three subtypes
- MDS, unclassifiable entity has been eliminated
- Refractory cytopenia of childhood is replaced by a new term, ‘Childhood MDS with low blasts’, and moved to a new section of childhood MDS

## 7.2 Diagnostic features

The WHO 2022 and ICC 2022 classifications have also refined the diagnostic features for each MDS type since the WHO 2017 blue book was published (Table 24).

Table 24. Diagnostic features for MDS as defined by the WHO 2017 blue book, ICC 2022 and WHO 2022 classifications

| **Classification document** | **Entity name** | **Dysplastic lineages** | **Cytopenias** | **Cytoses*** | **RS as % of marrow**  **erythroid elements** | **BM and PB blasts** | **Cytogenetics^†^** | **Mutations** |
| --- | --- | --- | --- | --- | --- | --- | --- | --- |
| **ICC 2022** | MDS with del(5q) | Typically ≥1[^‡^](javascript:;) | ≥1 | Thrombo-cytosis allowed |  | <5% BM <2% PB[§](javascript:;) | del(5q), with up to one additional abnormality, except −7/del(7q) | Any, except multi-hit *TP53* |
| **WHO 2017** | MDS with isolated del(5q) | 1–3 | 1–2 |  | None or any | <5% BM  <1% PB  No Auer rods | del(5q) alone or with one additional abnormality, except −7/del(7q) |  |
| **WHO 2022** | MDS with low blasts and isolated 5q deletion |  |  |  |  | <5% BM <2% PB | del(5q) alone or with one additional abnormality, except −7/del(7q) |  |
| **ICC 2022** | MDS with mutated *SF3B1* | Typically ≥1[^‡^](javascript:;) | ≥1 | 0 |  | <5% BM <2% PB | Any, except isolated del(5q), −7/del(7q), abn3q26.2, or complex | *SF3B1* (≥10% VAF), without multi-hit *TP53*, or *RUNX1* |
| **WHO 2017** | MDS with ring sideroblasts and single lineage dysplasia | 1 | 1–2 |  | ≥15% / ≥5%^a^ | <5% BM  <1% PB  No Auer rods | Any, unless fulfils all criteria for MDS with isolated del(5q) |  |
| **WHO 2017** | MDS with ring sideroblasts and multilineage dysplasia | 2–3 | 1–3 |  | ≥15% / ≥5%^a^ | <5% BM  <1% PB  No Auer rods | Any, unless fulfils all criteria for MDS with isolated del(5q) |  |
| **WHO 2022** | MDS with low blasts and *SF3B1* mutation^b^ |  |  |  |  | <5% BM <2% PB | Absence of 5q deletion, monosomy 7, or complex karyotype | *SF3B1* |

| **Classification document** | **Entity name** | **Dysplastic lineages** | **Cytopenias** | **Cytoses*** | **RS as % of marrow**  **erythroid elements** | **BM and PB blasts** | **Cytogenetics^†^** | **Mutations** |
| --- | --- | --- | --- | --- | --- | --- | --- | --- |
| **ICC 2022** | MDS with mutated *TP53* |  | Any |  |  | 0–9% BM or PB |  | Multi-hit *TP53* mutation* or *TP53* mutation (VAF >10%) and complex karyotype often with loss of 17p[^†^](javascript:;) |
| **WHO 2022** | MDS with biallelic *TP53* inactivation |  |  |  |  | <20% BM  <20% PB | Usually complex | Two or more *TP53* mutations, or one  mutation with evidence of *TP53* copy number loss or cnLOH |
| **WHO 2022** | MDS, NOS without dysplasia | 0 | ≥1 | 0 |  | <5% BM <2% PB[^§^](javascript:;) | −7/del(7q) or complex | Any, except multi-hit *TP53* or *SF3B1*  (≥10% VAF) |
| **ICC 2022** | MDS, NOS with single lineage dysplasia | 1 | ≥1 | 0 |  | <5% BM <2% PB[^§^](javascript:;) | Any, except not meeting criteria for MDS with del(5q) | Any, except multi-hit *TP53;* not meeting criteria for MDS with mutated *SF3B1* |
| **WHO 2017** | MDS with single lineage dysplasia | 1 | 1–2 |  | <15% / <5%^a^ | <5% BM  <1% PB  No Auer rods | Any, unless fulfils all criteria for MDS with isolated del(5q) |  |
| **WHO 2017** | MDS, NOS with multilineage dysplasia | ≥2 | ≥1 | 0 |  | <5% BM <2% PB[^§^](javascript:;) | Any, except not meeting criteria for MDS with del(5q) | Any, except multi-hit *TP53*; not meeting criteria for MDS with mutated *SF3B1* |
| **WHO 2022** | MDS with multilineage dysplasia | 2–3 | 1–3 |  | <15% / <5%^a^ | <5% BM  <1% PB  No Auer rods | Any, unless fulfils all criteria for MDS with isolated del(5q) |  |
| **WHO 2022** | MDS with low blasts |  |  |  |  | <5% BM <2% PB |  |  |
| **Classification document** | **Entity name** | **Dysplastic lineages** | **Cytopenias** | **Cytoses*** | **RS as % of marrow**  **erythroid elements** | **BM and PB blasts** | **Cytogenetics^†^** | **Mutations** |
| **WHO 2022** | MDS, hypoplastic^c^ |  |  |  |  | <5% BM <2% PB |  |  |
| **ICC 2022** | MDS with excess blasts | Typically ≥1[^‡^](javascript:;) | ≥1 | 0 |  | 5–9% BM, 2–9% PB[^§^](javascript:;) | Any | Any, except multi-hit *TP53* |
| **WHO 2017** | MDS with excess blasts  MDS-EB-1 | 1–3 | 1–3 |  | None or any | 5–9% BM or 2–4% PB, <10% BM and <5% PB  No Auer rods | Any |  |
| **WHO 2022** | MDS with increased blasts  MDS-IB1 |  |  |  |  | 5–9% BM or 2–4% PB |  |  |
| **ICC 2022** | MDS/AML | Typically ≥1[^‡^](javascript:;) | ≥1 | 0 |  | 10–19% BM or PB[^¶^](javascript:;) | Any, except  AML-defining | Any, except *NPM1*, bZIP *CEBPA* or *TP53* |
| **WHO 2017** | MDS with excess blasts  MDS-EB-2 | 1–3 | 1–3 |  | None or any | 10–19% BM or 5–19% PB or Auer rods, <20% BM and PB | Any |  |
| **WHO 2022** | MDS with increased blasts  MDS-IB2 |  |  |  |  | 10–19% BM or 5–19%  PB or Auer rods |  |  |
| **WHO 2022** | MDS with increased blasts  MDS with fibrosis |  |  |  |  | 5–19% BM; 2–19% PB |  |  |

Updated since WHO 2017 (terminology/wording aligned between ICC 2022 and WHO 2022)

Updated since WHO 2017 (terminology/wording different between ICC 2022 and WHO 2022)

Minimal or no changes since WHO 2017

**Key**

AML, acute myeloid leukaemia; BM, bone marrow; cnLOH, copy neutral loss of heterozygosity; MDS, myelodysplastic syndrome; NOS, not otherwise specified; PB, peripheral blood; RS, ring sideroblasts

*Cytoses: sustained white blood count ≥13 × 10^9^/L, monocytosis (≥0.5 × 10^9^/L and ≥10% of leukocytes) or platelets ≥450 × 10^9^/L; thrombocytosis is allowed in MDS with del(5q) or in any MDS case with inv(3) or t(3;3) cytogenetic abnormality.

^†^*BCR::ABL1* rearrangement or any of the rearrangements associated with myeloid/lymphoid neoplasms with eosinophilia and tyrosine kinase gene fusions exclude a diagnosis of MDS, even in the context of cytopenia.

^‡^Although dysplasia is typically present in these entities, it is not required.

^§^Although 2% PB blasts mandates classification of an MDS case as MDS with excess blasts, the presence of 1% PB blasts confirmed on two separate occasions also qualifies for MDS with excess blasts.

^¶^For paediatric patients (<18 years old), the blast thresholds for MDS with excess blasts are 5–19% in BM and 2–19% in PB, and the entity MDS/AML does not apply.

^a^If *SF3B1* mutation is present.

^b^Detection of ≥15% ring sideroblasts may substitute for *SF3B1* mutation. Acceptable related terminology: MDS with low blasts and ring sideroblasts.

^c^By definition, ≤25% bone marrow cellularity, age adjusted.

## 7.3 MDS/AML

The WHO 2017 blue book stated that ‘progression to AML is the natural course in many cases of MDS’ and specifies a 20% blast cut-off to delineate MDS from AML. The ICC 2022 classification retains the 20% bast threshold to define AML but introduces the term ‘MDS/AML’ (defined as a cytopenic myeloid neoplasm and 10–19% blasts in the blood or bone marrow), to replace the MDS-EB-2 subentity. In the WHO 2022 classification the 20% blast cut-off is also retained but the boundary between MDS and AML is softened. There was broad agreement that the new term ‘MDS-IB2’ in the WHO 2022 classification “may be regarded as AML-equivalent for therapeutic considerations and from a clinical trial design perspective when appropriate.”

## 7.4 *TP53* mutation

The ICC 2022 classification groups together MDS, MDS/AML and AML with mutated *TP53* under a new disease category – myeloid neoplasms with mutated *TP53*. These diseases are grouped together because of their overall similar aggressive behaviour irrespective of blast percentage, warranting a more unified treatment strategy across the blast spectrum. The diagnostic features distinguishing MDS, MDS/AML and AML with mutated *TP53* can be found in Table 25.

In the WHO 2022 classification, patients with MDS with multi-hit *TP53* alterations are categorised under a new entity, MDS with biallelic *TP53*, regardless of blast percentage.

Table 25. Diagnostic features distinguishing MDS, MDS/AML and AML with mutated *TP53* as defined by the ICC 2022 classification

| **ICC 2022 diagnostic features for myeloid neoplasms with mutated *TP53*** | | | |
| --- | --- | --- | --- |
| **Type** | **Cytopenia** | **Blasts** | **Genetics** |
| MDS with mutated *TP53* | Any | 0–9% bone marrow and blood blasts | Multi-hit *TP53* mutation* or *TP53* mutation (VAF >10%) and complex karyotype often with loss of 17p[^†^](javascript:;) |
| MDS/AML with mutated *TP53* | Any | 10–19% bone marrow or blood blasts | Any somatic *TP53* mutation (VAF >10%) |
| AML with mutated *TP53* | Not required | ≥20% bone marrow or blood blasts or meets criteria for pure erythroid leukaemia | Any somatic *TP53* mutation (VAF >10%) |

LOH, loss of heterozygosity; MDS, myelodysplastic syndrome; VAF, variant allele frequency.

*Defined as 2 distinct *TP53* mutations (each VAF >10%) or a single *TP53* mutation with (1) 17p deletion on cytogenetics; (2) VAF of >50%; or (3) copy-neutral LOH at the 17p *TP53* locus.

^†^If *TP53* locus LOH information is not available.

# 8. Paediatric myeloid disorders

There was no specific section in the WHO 2017 blue book that covers paediatric myeloid disorders, instead these disorders were included within the main sections of the blue book e.g. refractory cytopenia of childhood falls under MDS. However, the ICC 2022 and WHO 2022 classifications both have separate sections for paediatric myeloid disorders.

## 8.1 ICC 2022 updates

The ICC 2022 classification includes a section named ‘paediatric disorders and/or germline mutation-associated disorders’ (Table 26). This section of the ICC 2022 classification includes disorders that are unique to childhood as well as those associated with germline genetic pre-disposition as often, they have overlapping features.

Table 26. Paediatric and/or germline mutation-associated disorders as defined by the ICC 2022 classification

| **ICC 2022 paediatric disorders and/or germline mutation-associated disorders** |
| --- |
| Juvenile myelomonocytic leukaemia (JMML) |
| Juvenile myelomonocytic leukaemia-like neoplasms |
| Noonan syndrome-associated myeloproliferative disorder |
| Refractory cytopenia of childhood (RCC) |
| Haematological neoplasms with germline predisposition |

### 8.1.1 JMML, JMML-like neoplasms and Noonan syndrome-associated myeloproliferative disorder

The diagnostic features of these disorders are discussed in the MDS/MPN section of this document.

### 8.1.2 Refractory cytopenia of childhood (RCC)

In the WHO 2017 blue book, RCC was categorised under MDS. This disorder is characterised by persistent cytopenia, the presence of dysplasia and <5% blasts in the bone marrow and <2% blasts in the peripheral blood. The diagnostic criteria for RCC have been updated in the ICC 2022 classification (Table 27).

Table 27. Diagnostic criteria for RCC as defined by the ICC 2022 classification

| **ICC 2022 diagnostic criteria for RCC** | | | | |
| --- | --- | --- | --- | --- |
| 1. Persistent cytopenia  - Number of cytopenias (1–3). Cytopenia is defined according to age-adjusted values for haemoglobin, absolute neutrophil count and platelets | | | | |
| 1. Manifestation of dysplasia  - Dysplastic changes in at least two lineages or in ≥10% in one lineage - Typical dysplastic features of RCC (not all are required) | | | | |
| **Specimen** | **Cellularity** | **Erythropoiesis** | **Granulopoiesis** | **Megakaryopoiesis*** |
| Bone marrow aspirate |  | - Nuclear budding - Multinuclearity - Megaloblastoid changes | - Pseudo-Pelger-Huët cells - Hypo- or agranularity | - Separated nuclear lobes - Round monolobated nucleus - Micromega-karyocytes |
| Bone marrow biopsy | - Patchy pattern in otherwise hypocellular marrow   or   - Rarely diffuse pattern in normo- or hypercellular marrow[^†^](javascript:;) | - Patchy (few multifocal clusters or unifocal cluster) - Left-shift - Increased mitosis | - Marked decrease | - Marked decrease or aplasia - Round monolobated nucleus - Separated nuclear lobes - Micromega-karyocytes |
| 1. Other required criteria  - Blast percentage in peripheral blood <2% and bone marrow <5% - No prior cytotoxic chemotherapy or radiation therapy - No fibrosis | | | | |

*Immunohistochemistry for megakaryocyte markers is required.

^†^Normo- or hypocellular RCC requires significant dysplasia in megakaryocytes (>30%).

### 8.1.3 Haematological neoplasms with germline predisposition

The diagnostic features of these disorders are discussed in the myeloid neoplasms with germline predisposition section of this document.

## 8.2 WHO 2022 updates

The WHO 2022 classification includes a section named ‘childhood myelodysplastic neoplasms (childhood MDS)’ (Table 28). Childhood MDS are clonal haematopoietic stem cell neoplasms arising in children and adolescents (<18 years of age) leading to ineffective haematopoiesis, cytopenia(s) and risk of progression to AML.

Table 28. Categories of childhood MDS as defined by the WHO 2022 classification

| **WHO 2022 childhood MDS** | |
| --- | --- |
|  | **Blasts** |
| **Childhood MDS with low blasts** | <5% bone marrow; <2% peripheral blood |
| Hypocellular |  |
| Not otherwise specified (NOS) |  |
| **Childhood MDS with increased blasts** | 5–19% bone marrow;  2–19% peripheral blood |

### 8.2.1 Childhood MDS with low blasts

This entity replaces the former term ‘refractory cytopenia of childhood’ in the WHO 2017 blue book and includes two subtypes: childhood MDS with low blasts, hypocellular; and childhood MDS with low blasts, not otherwise specified (NOS). Exclusion of non-neoplastic causes of cytopenia such as infections, nutritional deficiencies, metabolic diseases, bone marrow failure syndromes (BMFS) and germline pathogenic variants remains an essential diagnostic prerequisite for childhood MDS with low blasts.

### 8.2.2 Childhood MDS with increased blasts

This entity is defined as having ≥5% blasts in the bone marrow or ≥2% blasts in the peripheral blood. Acquired cytogenetic abnormalities and RAS-pathway mutations are more common in childhood MDS with increased blasts compared with childhood MDS with low blasts.

# 9. Acute myeloid leukaemia (AML)

AML results from the clonal expansion of myeloid blasts in the peripheral blood, bone marrow or other tissue. It is a heterogeneous disease clinically, morphologically and genetically, and can involve a single myeloid lineage or multiple myeloid lineages.

## 9.1 Categorisation

The updated WHO 2022 and ICC 2022 classifications retain many of the previously defined AML entities in the WHO 2017 blue book with additional genetically related entities included. The differences between each classification document are listed in Table 29.

Table 29. AML entities and corresponding blast cut-offs (in blue font) as defined by the WHO 2017 blue book, ICC 2022 and WHO 2022 classifications

| **ICC 2022** | **WHO 2017** | **WHO 2022** |
| --- | --- | --- |
|  | **AML with recurrent genetic abnormalities** | **AML with defining genetic abnormalities** |
| AML with t(8;21)(q22;q22.1)/ *RUNX1::RUNX1T1*  ≥10% | AML with t(8;21)(q22;q22.1); *RUNX1-RUNX1T1*  (Considered as leukaemia without regard to blast amount) | AML with *RUNX1::RUNX1T1* fusion  (may be diagnosed with  <20% blasts) |
| AML with inv(16)(p13.1q22) or t(16;16)(p13.1;q22)/ *CBFB::MYH11*  ≥10% | AML with inv(16)(p13.1q22) or t(16;16)(p13,1;q22);  *CBFB-MYH11*  (Considered as leukaemia without regard to blast amount) | AML with *CBFB::MYH11* fusion  (may be diagnosed with  <20% blasts) |
| Acute promyelocytic leukaemia with t(15;17)(q24.1;q21.2)/  *PML*::*RARA*  ≥10% | Acute promyelocytic leukaemia with *PML-RARA*  (Considered as leukaemia without regard to blast amount) | Acute promyelocytic leukaemia with *PML::RARA* fusion  (may be diagnosed with  <20% blasts) |
| Acute promyelocytic leukaemia with other *RARA* rearrangements[*](javascript:;)  ≥10% |  |  |
| AML with t(9;11)(p21.3;q23.3)/  *MLLT3::KMT2A*  ≥10% | AML with t(9;11)(p21.3;q23.3);  *KMT2A-MLLT3*  ≥20%^a^ | AML with *KMT2A* rearrangement  (may be diagnosed with  <20% blasts) |
| AML with other *KMT2A* rearrangements^†^  ≥10% |  |  |
| AML with t(6;9)(p22.3;q34.1)/  *DEK::NUP214*  ≥10% | AML with t(6;9)(p23;q34.1); *DEK-NUP214*  ≥20%^a^ | AML with *DEK::NUP214* fusion  (may be diagnosed with  <20% blasts) |
|  |  | AML with *NUP98* rearrangement  (may be diagnosed with  <20% blasts) |
| AML with inv(3)(q21.3q26.2) or t(3;3)(q21.3;q26.2)/*GATA2; MECOM(EVI1)*  ≥10% | AML with inv(3)(q21.3q26.2) or t(3;3)(q21.3;q26,2); *GATA2, MECOM*  ≥20%^a^ | AML with *MECOM* rearrangement  (may be diagnosed with  <20% blasts) |
| AML with other *MECOM* rearrangements^‡^  ≥10% |  |  |
| (Categorised under AML with other rare recurring translocations)  ≥10% | AML (megakaryoblastic) with t(1;22)(p13.3;q13.1);  *RBM15-MKL1*  ≥20%^a^ | AML with *RBM15::MRTFA* fusion  (may be diagnosed with  <20% blasts) |
| AML with t(9;22)(q34.1;q11.2)/  *BCR::ABL1*§  ≥20% | *AML with BCR-ABL1*  ≥20%^a^ | AML with *BCR::ABL1* fusion  ≥20% |
| AML with mutated *NPM1*  ≥10% | AML with mutated *NPM1*  ≥20% | AML with *NPM1* mutation  can be diagnosed  irrespective of the blast count |
| AML with in-frame bZIP *CEBPA* mutations  ≥10% | AML with biallelic mutation of *CEBPA*  ≥20% | AML with *CEBPA* mutation located in the basic leucine zipper (bZIP) region (smbZIP-CEBPA)  ≥20% |
| (Categorised under AML or MDS/AML with myelodysplasia-related gene mutations) | *AML with mutated RUNX1*  ≥20% |  |
| AML and MDS/AML with mutated *TP53*[^†^](javascript:;)  10–19% (MDS/AML) and  ≥20% (AML) | (Did not recognise AML or MDS/AML with TP53 mutation as distinct entities) | (Includes MDS with biallelic TP53, regardless of blast percentage) |
| AML with other rare recurring translocations^¶^  ≥10% |  | AML with other defined genetic alterations^¶^  ≥20% |
| AML and MDS/AML with myelodysplasia-related gene mutations:  10–19% (MDS/AML) and  ≥20% AML | **AML with myelodysplasia-related changes**  ≥20% | AML, myelodysplasia-related (AML-MR)  ≥20% |
| AML with myelodysplasia-related cytogenetic abnormalities  10–19% (MDS/AML) and  ≥20% AML |  |  |
| (Now appended as a diagnostic qualifier) | **Therapy-related myeloid neoplasms** | (Now appended as a diagnostic qualifier) |
| AML, not otherwise specified  10–19% (MDS/AML) and  ≥20% (AML) | **AML, not otherwise specified**  ≥20% | **AML, defined by differentiation**  ≥20% |
|  | AML with minimal differentiation | AML with minimal differentiation |
|  | AML without maturation | AML without maturation |
|  | AML with maturation | AML with maturation |
|  | Acute myelomonocytic leukaemia | Acute myelomonocytic leukaemia |
|  | Acute monoblastic and monocytic leukaemia | Acute monocytic leukaemia |
|  | Pure erythroid leukaemia | Acute erythroid leukaemia |
|  | Acute megakaryoblastic leukaemia | Acute megakaryoblastic leukaemia |
|  | Acute basophilic leukaemia | Acute basophilic leukaemia |
|  | Acute panmyelosis with myelofibrosis |  |
| Myeloid sarcoma | Myeloid sarcoma | Myeloid sarcoma |

Italic font indicates provisional tumour entities.

Updated since WHO 2017 (terminology/wording aligned between ICC 2022 and WHO 2022)

Updated since WHO 2017 (terminology/wording different between ICC 2022 and WHO 2022)

Minimal or no changes since WHO 2017

**Key**

*****Includes AMLs with t(1;17)(q42.3;q21.2)/*IRF2BP2::RARA*; t(5;17)(q35.1;q21.2)/*NPM1::RARA*; t(11;17)(q23.2;q21.2)/*ZBTB16::RARA*; cryptic inv(17q) or del(17) (q21.2q21.2)/*STAT5B::RARA*, *STAT3::RARA*; Other genes rarely rearranged with *RARA:TBL1XR1* (3q26.3), *FIP1L1* (4q12), *BCOR* (Xp11.4).

^†^Includes AMLs with t(4;11)(q21.3;q23.3)/*AFF1::KMT2A*^#^; t(6;11)(q27;q23.3)/*AFDN::KMT2A*; t(10;11)(p12.3;q23.3)/*MLLT10::KMT2A*; t(10;11)(q21.3;q23.3)/*TET1::KMT2A*; t(11;19)(q23.3;p13.1)/
*KMT2A::ELL*; t(11;19)(q23.3;p13.3)/*KMT2A::MLLT1* (occurs predominantly in infants and children).

^‡^Includes AMLs with t(2;3)(p11∼23;q26.2)/*MECOM::?*; t(3;8)(q26.2;q24.2)/*MYC*, *MECOM*; t(3;12)(q26.2;p13.2)/*ETV6::MECOM*; t(3;21)(q26.2;q22.1)/*MECOM::RUNX1*.

^§^The category of MDS/AML will not be used for AML with *BCR::ABL1*due to its overlap with progression of CML, *BCR::ABL1*-positive.

^¶^See Supplementary Table 4.

^a^It is controversial whether all cases should be categorised as AML when the blast amount is <20%.

**ICC 2022 changes to AML entities**

- Percentage of blasts required for diagnosis has been added to each entity
- Three new entities are included: Acute promyelocytic leukaemia with other *RARA* rearrangements, AML with other *KMT2A* rearrangements and AML with other *MECOM* rearrangements
- AML (megakaryoblastic) with t(1;22)(p13.3;q13.1);*RBM15-MRTF1* (formerly *MKL1*) entity is now categorised under AML with other rare recurring translocations
- AML with in-frame bZIP CEBPA mutations (≥10%) replaces AML with biallelic mutation of CEBPA
- AML with mutated *RUNX1* omitted as a provisional entity. *RUNX1* mutation included under a new category – AML and MDS/AML with myelodysplasia-related gene mutations (Table 31)
- AML and MDS/AML with mutated *TP53* is now recognised as a separate entity (this is not the case in WHO 2022)
- AML with other rare recurring translocations added as a new entity
- AML with myelodysplasia-related changes category is eliminated and replaced with two new entities: AML and MDS/AML with myelodysplasia-related gene mutations and AML with myelodysplasia-related cytogenetic abnormalities (Table 31)
- Therapy-related myeloid neoplasms omitted as a category and ‘therapy-related’ diagnostic qualifier to be used instead
- Morphological or cytochemical subtypes of AML, not otherwise specified (AML, NOS) e.g. AML with minimal differentiation are not included

**WHO 2022 changes to AML entities**

- AML is arranged into two families: AML with defining genetic abnormalities and AML defined by differentiation
- AML with *NUP98* rearrangement is recognised as a new entity
- The definition of AML with *CEBPA* mutation has changed to include biallelic (biCEBPA) as well as single mutations located in the basic leucine zipper (bZIP) region of the gene (smbZIP-CEBPA, ≥20%)
- AML with somatic *RUNX1* mutation is not recognised as a distinct disease type due to lack of sufficient unifying characteristics
- AML with other defined genetic alterations added as a new category and includes AML with rare fusions
- AML with myelodysplasia-related changes replaced with AML, myelodysplasia-related and included within the AML with defining genetic abnormalities family (Table 31)
- Therapy-related myeloid neoplasms omitted as a category and ‘post cytotoxic therapy’ diagnostic qualifier to be used instead
- AML, not otherwise specified is eliminated (replaced by AML, defined by differentiation)

## 9.2 Percentage of blasts required for AML diagnosis

In the WHO 2017 blue blook, the defining criterion for AML was the presence of ≥20% myeloid blasts in the peripheral blood or bone marrow. However, the blue book also stated that ‘AML with t(8;21)(q22;q22.1), AML with inv(16)(p13.1q22) or t(16;16)(p13.1;q22), and acute promyelocytic leukaemia with PML-RARA are considered to be acute leukaemias without regard to blast cell count’. These blast thresholds for defining AML have since been updated in the ICC 2022 and WHO 2022 classifications.

**ICC 2022 changes to the blast thresholds defining AML**

- AML with recurrent genetic abnormalities require ≥10% blasts (BM or PB)
- Other categories: cases with 10–19% blasts (BM or PB) now designated ‘MDS/AML’ (former MDS-EB2 entity now eliminated), and cases with ≥20% are designated ‘AML’

**WHO 2022 changes to the blast thresholds defining AML**

- Blast cut-offs have been eliminated for AML entities with genetic abnormalities except for AML with *BCR::ABL1* fusion and AML with *CEBPA* mutation which remain at ≥20%
- ≥20% blast cut-off retained for AML, defined by differentiation subtypes

## 9.3 Therapy-related myeloid neoplasms vs. diagnostic qualifiers

The WHO 2017 blue book defined therapy-related cases of AML, and prior MDS and MDS/MPN in a separate group – therapy-related myeloid neoplasms, which occur as a late complication of cytotoxic chemotherapy and/or radiation therapy administered for a prior neoplastic or non-neoplastic disorder. However, the ICC 2022 and WHO 2022 classifications no longer consider therapy-related myeloid neoplasms as a distinct disease entity, the use of diagnostic qualifiers is proposed instead.

The ICC 2022 classification recommends including prior therapy, antecedent myeloid neoplasms (i.e. MDS or MDS/MPN) and underlying germline genetic disorders as qualifiers to the diagnosis rather than as specific disease categories (Table 30).

Table 30. Diagnostic qualifiers as defined by the ICC 2022 classification

| **ICC 2022 diagnostic qualifiers that should be used following a**  **specific MDS, AML (or MDS/AML) diagnosis** |
| --- |
| Therapy-related[*](javascript:;)   - Prior chemotherapy, radiotherapy, immune interventions |
| Progressing from MDS • MDS should be confirmed by standard diagnostics |
| Progressing from MDS/MPN (specify)   - MDS/MPN should be confirmed by standard diagnostics |
| Germline predisposition |

Examples: AML with myelodysplasia-related cytogenetic abnormality, therapy-related; AML with myelodysplasia-related gene mutation, progressed from MDS; AML with myelodysplasia-related gene mutation, germline *RUNX1* mutation.

*Lymphoblastic leukaemia/lymphoma may also be therapy-related, and that association should also be noted in the diagnosis.

The WHO 2022 classification includes a newly segregated category – secondary myeloid neoplasms, encompassing myeloid neoplasms that arise secondary to exposure to cytotoxic therapy and myeloid neoplasms associated with germline predisposition. The WHO 2022 classification recommends adding ‘post cytotoxic therapy’ and ‘associated with germline [gene] variant’ as qualifiers to relevant myeloid disease types whose criteria are fulfilled as defined elsewhere in the classification, e.g. AML with *KMT2A* rearrangement post cytotoxic therapy or MDS with low blasts associated with germline *RUNX1* variant.

## 9.4 AML with myelodysplasia-related changes (AML-MR)

In the WHO 2017 blue book, AML with myelodysplasia-related changes was characterised by ≥20% peripheral blood or bone marrow blasts; multilineage dysplasia, a history of MDS or MPN or MDS-related cytogenetic abnormalities and no specific genetic abnormalities characteristic of AML with recurrent genetic abnormalities. In addition, patients should not have a history of prior cytotoxic or radiation therapy for an unrelated disease. The ICC 2022 and WHO 2022 classifications have since refined this AML subtype.

The ICC 2022 classification has eliminated AML with myelodysplasia-related changes and replaced it with two new categories:

- AML and MDS/AML with myelodysplasia-related gene mutations (10–19% blasts required for MDS/AML and ≥20% for AML)
- AML with myelodysplasia-related cytogenetic abnormalities (10–19% blasts required for MDS/AML and ≥20% for AML)

The specific myelodysplasia-related gene mutations and cytogenetic abnormalities outlined in the ICC classification can be found in Table 31.

The WHO 2022 classification has renamed AML with myelodysplasia-related changes to AML, myelodysplasia-related (AML-MR) and updated the diagnostic criteria. Now, only the presence of one or more cytogenetic or molecular abnormalities listed in Table 31 and/or history of MDS or MDS/MPN are required for a diagnosis of AML-MR. AML transformation of MPN is retained within the MPN category.

Table 31. Myelodysplasia-related cytogenetic abnormalities and gene mutations as defined by the ICC 2022 and WHO 2022 classifications

| **ICC 2022** | **WHO 2022** |
| --- | --- |
| **Myelodysplasia-related cytogenetic abnormalities**   - Complex karyotype (≥3 unrelated clonal chromosomal abnormalities in the absence of other class-defining recurring genetic abnormalities) - del(5q)/t(5q)/add(5q) - −7/del(7q) - +8 - del(12p)/t(12p)/add(12p) - −17/add(17p) or del(17p) - i(17q) - idic(X)(q13) - del(20q) | **Cytogenetic abnormalities defining AML-MR**   - Complex karyotype (≥3 abnormalities) - 5q deletion or loss of 5q due to unbalanced translocation - Monosomy 7, 7q deletion, or loss of 7q due to unbalanced translocation - 11q deletion - 12p deletion or loss of 12p due to unbalanced translocation - Monosomy 13 or 13q deletion - 17p deletion or loss of 17p due to unbalanced translocation - Isochromosome 17q - idic(X)(q13) |
| **Myelodysplasia-related gene mutations**   - *ASXL1* - *BCOR* - *EZH2* - *RUNX1* - *SF3B1* - *SRSF2* - *STAG2* - *U2AF1* - *ZRSR2* | **Somatic mutations defining AML-MR**   - *ASXL1* - *BCOR* - *EZH2* - *SF3B1* - *SRSF2* - *STAG2* - *U2AF1* - *ZRSR2* |

Alignment between ICC 2022 and WHO 2022

Differences between ICC 2022 and WHO 2022

**Key**

## 9.5 Acute myeloid leukaemia, NOS

The WHO 2017 blue book stated that AML, NOS “encompasses the cases that do not fulfil the criteria for inclusion in one of the previously described groups (i.e. AML with recurrent genetic abnormalities, myelodysplasia-related changes or therapy-related AML).” The blue book subclassified AML, NOS by morphological and cytochemical/immunophenotypic features.

In the ICC 2022 classification, the AML, NOS entity is retained for cases when all other genetic categories are excluded. The document states that “previously used morphologic or cytochemical subtypes of AML, NOS have limited prognostic significance, but pathologists may continue to subclassify such cases if desired.”

In the WHO 2022 classification, AML, NOS is renamed to “AML defined by differentiation” and includes cases that lack defining genetic abnormalities. The WHO 2022 classification provide updated diagnostic criteria for AML subtypes defined by differentiation (Table 32).

Table 32. Diagnostic criteria for AML subtypes defined by differentiation according to the WHO 2022 classification

| **WHO 2022 diagnostic criteria for AML subtypes defined by differentiation** | |
| --- | --- |
| **Type** | **Diagnostic criteria*** |
| AML with minimal differentiation | - Blasts are negative (<3%) for MPO and SBB by cytochemistry - Expression of two or more myeloid-associated antigens, such as CD13, CD33, and CD117 |
| AML without maturation | - ≥3% blasts positive for MPO (by immunophenotyping or cytochemistry) or SBB and negative for NSE by cytochemistry - Maturing cells of the granulocytic lineage constitute <10% of the nucleated bone marrow cells - Expression of two or more myeloid-associated antigens, such as MPO, CD13, CD33, and CD117 |
| AML with maturation | - ≥3% blasts positive for MPO (by immunophenotyping or cytochemistry) or SBB by cytochemistry - Maturing cells of the granulocytic lineage constitute ≥10% of the nucleated bone marrow cells - Monocyte lineage cells constitute <20% of bone marrow cells - Expression of two or more myeloid-associated antigens, such as MPO, CD13, CD33, and CD117 |
| Acute basophilic leukaemia | - Blasts and immature/mature basophils with metachromasia on toluidine blue staining - Blasts are negative for cytochemical MPO, SBB, and NSE - No expression of strong CD117 equivalent (to exclude mast cell leukaemia) |
| Acute myelomonocytic leukaemia | - ≥20% monocytes and their precursors - ≥20% maturing granulocytic cells - ≥3% of blasts positive for MPO (by immunophenotyping or cytochemistry) |
| Acute monocytic leukaemia | - ≥80% monocytes and/or their precursors (monoblasts and/or promonocytes) - <20% maturing granulocytic cells - Blasts and promonocytes expressing at least two monocytic markers including CD11c, CD14, CD36 and CD64, or NSE positivity on cytochemistry |
| Acute erythroid leukaemia | - ≥30% immature erythroid cells (proerythroblasts) - Bone marrow with erythroid predominance, usually ≥80% of cellularity |
| Acute megakaryoblastic leukaemia | - Blasts express at least one or more of the platelet glycoproteins: CD41 (glycoprotein llb), CD61 (glycoprotein IIIa), or CD42b (glycoprotein lb) |

BM, bone marrow; MPO, myeloperoxidase; NSE, nonspecific esterase; PB, peripheral blood; SBB, Sudan Black B.

*Shared diagnostic criteria include:

- ≥20% blasts in bone marrow and/or blood (except for acute erythroid leukaemia)
- Criteria for AML types with defined genetic alterations are not met
- Criteria for mixed-phenotype acute leukaemia are not met (relevant for AML with minimal differentiation)
- Not fulfilling diagnostic criteria for myeloid neoplasm post cytotoxic therapy
- No prior history of myeloproliferative neoplasm

## 9.6 Myeloid sarcoma

A myeloid sarcoma is a tumour mass consisting of myeloid blasts, with or without maturation, occurring at an anatomical site other than the bone marrow. Its definition and diagnostic criteria in the WHO 2017 blue book remain unchanged in both the ICC 2022 and WHO 2022 classifications.

# 10. Myeloid neoplasms with germline predisposition

Some cases of myeloid neoplasm, in particular MDS and AML, occur in association with inherited or *de novo* germline mutations characterised by specific genetic and clinical findings. Recognising and diagnosing these disorders that arise from a germline mutation is essential for effective patient management.

## 10.1 Diagnostic qualifiers

In the WHO 2017 blue book ‘myeloid neoplasms with germline predisposition’ were included as a separate section. However, in the ICC 2022 classification, this group of disorders is included within a new section, ‘Paediatric disorders and/or germline mutation-associated disorders.’ The ICC 2022 classification recommends that “any underlying germline predisposition mutation or syndrome should be specified as a qualifier after the MDS, AML, or other malignancy diagnosis and subtype.”

In the WHO 2022 classification, myeloid neoplasms with germline predisposition are also included within a newly segregated category – secondary myeloid neoplasms. Myeloid neoplasms associated with germline predisposition include AML, MDS, MPN, and MDS/MPN that arise in individuals with genetic conditions associated with increased risk of myeloid malignancies. The WHO 2022 classification recommends adding ‘associated with germline [gene] variant’ as a qualifier to relevant myeloid disease types whose criteria are fulfilled as defined elsewhere in the classification, e.g. MDS with low blasts associated with germline RUNX1 variant.

## 10.2 Categorisation

Myeloid neoplasms with germline disposition entities from the WHO 2017 blue book are included within the ICC 2022 and WHO 2022 classifications but new entities have also been discovered and added to the 2022 classification documents. The WHO 2022 classification presents a restructured diagnostic framework for myeloid neoplasms associated with germline predisposition that is scalable and can accommodate future refinement and discoveries. These diseases are now classified using a formulaic approach that couples the myeloid disease phenotype with the predisposing germline genotype, e.g., AML with germline pathogenic variants in *RUNX1*.

The differences between each document are listed in Table 33. In addition, the ICC 2022 document has introduced the term ‘haematological neoplasms’ to replace ‘myeloid neoplasms’ which is used in the WHO 2017 blue book and the WHO 2022 classification.

Table 33. Myeloid (or haematological) neoplasms with germline disposition entities as defined by the WHO 2017 blue book, ICC 2022 and WHO 2022 classifications

| **ICC 2022** | **WHO 2017** | **WHO 2022** |
| --- | --- | --- |
| **Haematological neoplasms with germline predisposition without a constitutional disorder affecting multiple organ systems** |  | **Myeloid neoplasms with germline predisposition without a pre-existing platelet disorder or organ dysfunction** |
| Myeloid neoplasms with germline *CEBPA* mutation | Acute myeloid leukaemia with germline *CEBPA* mutation | Myeloid neoplasms with germline *CEBPA* P/LP variant (CEBPA-associated familial AML) |
| Myeloid or lymphoid neoplasms with germline *DDX41* mutation | Myeloid neoplasms with germline *DDX41* mutation | Myeloid neoplasms with germline *DDX41* P/LP variant^a^ |
| Myeloid or lymphoid neoplasms with germline *TP53* mutation |  | Myeloid neoplasms with germline *TP53* P/LP variant^a^ (Li-Fraumeni syndrome) |
| **Haematological neoplasms with germline predisposition associated with a constitutional platelet disorder** |  | **Myeloid neoplasms with germline predisposition and pre-existing platelet disorder** |
| Myeloid or lymphoid neoplasms with germline *RUNX1* mutation | Myeloid neoplasms with germline *RUNX1* mutation | Myeloid neoplasms with germline *RUNX1* P/LP variant^a^ (familial platelet disorder with associated myeloid malignancy, FPD-MM) |
| Myeloid neoplasms with germline *ANKRD26* mutation | Myeloid neoplasms with germline *ANKRD26* mutation | Myeloid neoplasms with germline *ANKRD26* P/LP variant^a^ (Thrombocytopenia 2) |
| Myeloid or lymphoid neoplasms with germline *ETV6* mutation | Myeloid neoplasms with germline *ETV6* mutation | Myeloid neoplasms with germline *ETV6* P/LP variant^a^ (thrombocytopenia 5) |
| **Haematological neoplasms with germline predisposition associated with a constitutional disorder affecting multiple organ systems** |  | **Myeloid neoplasms with germline predisposition and potential organ dysfunction** |
| Myeloid neoplasms with germline *GATA2* mutation | Myeloid neoplasms with germline *GATA2* mutation | Myeloid neoplasms with germline *GATA2* P/LP variant (GATA2-deficiency) |
| Myeloid neoplasms associated with bone marrow failure syndromes   - Fanconi anaemia - Shwachman-Diamond syndrome - Telomere biology disorders including dyskeratosis congenita - Severe congenital neutropenia - Diamond-Blackfan anaemia |  | Bone marrow failure syndromes   - Severe congenital neutropenia (SCN) - Shwachman-Diamond syndrome (SDS) - Fanconi anaemia (FA) |
|  |  | - Telomere biology disorders |
| JMML associated with neurofibromatosis |  | RASopathies (neurofibromatosis type 1, CBL syndrome, Noonan syndrome or Noonan syndrome-like disorders^a^) |
| JMML associated with Noonan-syndrome-like disorder (CBL-syndrome) |  |  |
| Myeloid or lymphoid neoplasms associated with Down syndrome |  | Down syndrome^a^ |
| Myeloid neoplasms with germline *SAMD9* mutation |  | Myeloid neoplasms with germline *SAMD9* P/LP variant (MIRAGE syndrome) |
| Myeloid neoplasms with germline *SAMD9L* mutation |  | Myeloid neoplasms with germline *SAMD9L* P/LP variant (SAMD9L-related ataxia pancytopenia syndrome)^b^ |
|  |  | Myeloid neoplasms with biallelic germline *BLM* P/LP variant (Bloom syndrome) |
| **Acute lymphoblastic leukaemia (ALL) with germline predisposition**[**^c^**](javascript:;) |  |  |
| Acute lymphoblastic leukaemia with germline *PAX5* mutation |  |  |
| Acute lymphoblastic leukaemia with germline *IKZF1* mutation |  |  |

Updated since WHO 2017 (terminology/wording aligned between ICC 2022 and WHO 2022)

Updated since WHO 2017 (terminology/wording different between ICC 2022 and WHO 2022)

Minimal or no changes since WHO 2017

**Key**

P, pathogenic; LP, likely pathogenic.

^a^Lymphoid neoplasms can also occur.

^b^Ataxia is not always present.

^c^Down syndrome and germline mutations in *ETV6* or *TP53* also predispose to acute lymphoblastic leukaemia.

**ICC 2022 changes to haematological neoplasms with germline predisposition entities**

- Each entity has been organised under four subgroupings
- Myeloid or lymphoid neoplasms with germline *TP53* mutation is added as a new entity of haematological neoplasm with germline predisposition without a constitutional disorder affecting multiple organ systems
- Six new entities of haematological neoplasm with germline predisposition associated with a constitutional disorder affecting multiple organ systems have been added
- Two new entities of ALL with germline predisposition have been added

**WHO 2022 changes to myeloid neoplasms with germline predisposition entities**

- Each entity has been organised under three subgroupings
- Myeloid neoplasms with germline *TP53* P/LP variant (Li-Fraumeni syndrome) is added as a new entity of myeloid neoplasm with germline predisposition without a pre-existing platelet disorder or organ dysfunction
- Seven new entities of myeloid neoplasms with germline predisposition and potential organ dysfunction have been added

# 11. Myeloid proliferations associated with Down syndrome

The WHO 2017 blue book defined two types of myeloid proliferation associated with Down syndrome – transient abnormal myelopoiesis (TAM), which is confined to the first 6 months of life and myeloid leukaemia of Down syndrome (ML-DS). These entities and definitions remain unchanged in both the ICC 2022 and WHO 2022 classifications.

# 12. Conclusion

In summary, this paper has compared the ICC and WHO 2022 classifications for myeloid neoplasms and acute leukaemias and examined the updates to different entities that have been introduced since the WHO 2017 blue book was published.

The following references were used in the development of this paper and can provide further information, as required:

- Swerdlow SH, Campo E, Harris NL, et al., editors. WHO Classification of Tumours of Haematopoietic and Lymphoid Tissues. Revised 4th ed. IARC; 2017
- Arber DA, Orazi A, Hasserjian RP, et al. International Consensus Classification of Myeloid Neoplasms and Acute Leukemias: integrating morphologic, clinical, and genomic data. Blood 2022;140:1220–28
- Khoury JD, Solary E, Abla O, et al. The 5th edition of the World Health Organization Classification of Haematolymphoid Tumours: Myeloid and Histiocytic/Dendritic Neoplasms. Leukemia 2022;36:1703–19
- WHO Classification of Tumours Editorial Board. Haematolymphoid Tumours. 5th ed. [Internet; beta version ahead of print]. Available at: https://tumourclassification.iarc.who.int/chapters/63 [cited 17 October 2023]
- Leguit RJ, Wang SA, George TI, et al. The international consensus classification of mastocytosis and related entities. Virchows Arch 2023;482:99–112
- Valent P, Horny HP, Escribano L, et al. Diagnostic criteria and classification of mastocytosis: a consensus proposal. Leuk Res 2001;25:603–25
- Valent P, Akin C, Hartmann K, et al. Updated Diagnostic Criteria and Classification of Mast Cell Disorders: A Consensus Proposal. Hemasphere 2021;5:e646

# 13. Acknowledgements

The paper was co-authored by Konstanze Döhner, Kirsten Grønbæk, and António Medina Almeida. Medical writing support was provided by Elements Communications Ltd., UK and funded by the European Hematology Association.

# 14. Supplementary appendix

Supplementary Table 1. Features in chronic phase CML associated with increased risk of disease progression as defined by the WHO 2022 classification.

| **At diagnosis** | | |
| --- | --- | --- |
| - High ELTS score - 10–19% blasts in the peripheral blood and/or bone marrow^a,b^ - ≥20% basophils in the peripheral blood - Additional chromosomal abnormalities in Ph+ cells, including 3q26.2 rearrangements, monosomy 7, isochromosome 17q and complex karyotype - Additional chromosomal abnormalities in Ph+ cells, including trisomy 8, 11q23 rearrangements, trisomy 19, trisomy 21, additional Ph+ (evidence of association with disease progression less clear) - Clusters of small megakaryocytes (including true micromegakaryocytes similar to those seen in myelodysplastic syndromes), associated with significant reticulin and/or collagen fibrosis, which is best assessed in biopsy sections | | |
|  | | |
| **ELTS score** | 0.0025 × (**age/**10)^3^  + 0.0615 × **spleen size**  + 0.1052 × **peripheral blood blasts**  + 0.4104 × (**platelet count**/1000)^-0.5^ | Low- risk: <1.5680  Intermediate risk: 1.5680–2.2185  High-risk: >2.2185 |
|  | | |
| **Emerging on treatment** | | |
| Resistance to TKI as defined by ELN 2020, including loss of prior responses, emergence of ACA and BCR::ABL1 kinase domain mutations | | |

ACA, additional chromosome abnormality; ELN, European LeukemiaNet; ELTS, EUTOS long-term survival; EUTOS, European Treatment and Outcome Study; Ph, Philadelphia chromosome; TKI, tyrosine kinase inhibitor.

^a^The finding of bona fide lymphoblasts in the peripheral blood or bone marrow (even if <10%) is consistent with the diagnosis of blast phase.

^b^≥20% blasts in the peripheral blood or bone marrow, or an infiltrative proliferation of blasts in an extramedullary site, is diagnostic of blast phase.

Supplementary Table 2. Semiquantitative bone marrow fibrosis (MF) grading system proposed by Thiele J, et al. with minor modifications concerning collagen and osteosclerosis^a^ as defined by the WHO 2017 blue book.

| **Grade** | **Definition** |
| --- | --- |
| MF-0 | Scattered linear reticulin with no intersections (cross-overs), corresponding to normal bone marrow |
| MF-1 | Loose network of reticulin with many intersections, especially in perivascular areas |
| MF-2 | Diffuse and dense increase in reticulin with extensive intersections, occasionally with focal bundles of thick fibres mostly consistent with collagen and/or associated with focal osteosclerosis^b^ |
| MF-3 | Diffuse and dense increase in reticulin with extensive intersections and coarse bundles of thick fibres consistent with collagen, usually associated with osteosclerosis |

^a^Fibre density should be assessed only in haematopoietic areas; if the pattern is heterogeneous, the final grade is determined by the highest grade present in ≥30% of the marrow area.

^b^In grades MF-2 and MF-3, an additional trichrome stain is recommended.

Supplementary Table 3. B ('burden of disease’) and C ('cytoreduction-requiring') findings in systemic mastocytosis as defined by the WHO 2017 blue book, ICC 2022 and WHO 2022 classifications

| **B findings in SM** | | |
| --- | --- | --- |
| **ICC 2022** | **WHO 2017** | **WHO 2022** |
| 1. High mast cell burden, >30% of BM cellularity by mast cell aggregates (assessed on BM biopsy) and serum tryptase >200 ng/mL | 1. High mast cell burden (shown on BM biopsy); >30% infiltration of cellularity by  mast cells (focal, dense aggregates) and serum total tryptase >200 ng/mL | 1. High mast cell burden: infiltration grade mast cells in BM ≥30% in histology (IHC) and/or serum tryptase ≥200 ng/mL^a^ and/or *KIT* p.D816V VAF ≥10% in BM or PB leukocytes |
| 2. Cytopenia (not meeting criteria for C findings) or cytosis. Reactive causes are excluded, and criteria for other myeloid neoplasms are not met. | 2. Signs of dysplasia or myeloproliferation in non-mast cell lineage(s), but criteria are not met for definitive diagnosis of an associated haematological neoplasm, with normal or only slightly abnormal blood counts | 2. Signs of myeloproliferation and/or myelodysplasia^b^: hypercellular BM with loss of fat cells and prominent myelopoiesis ± left shift and eosinophilia ± leukocytosis and eosinophilia and/or discrete signs of myelodysplasia (<10% neutrophils, erythrocytes, and megakaryocytes) |
| 3. Hepatomegaly without impairment of liver function, or splenomegaly without features of hypersplenism including thrombocytopenia, and/or lymphadenopathy  (>1 cm size) on palpation or imaging | 3. Hepatomegaly without impairment of liver function, palpable splenomegaly without hypersplenism and/or  lymphadenopathy on palpation or imaging | 3. Organomegaly: Palpable (or documented by US, CT or MRI) hepatomegaly without ascites or other signs of organ damage or/and palpable splenomegaly without hypersplenism and without weight loss or/and lymphadenopathy palpable or visceral LN-enlargement found in ULS or CT (>20 mm) |
| **C findings in SM** | | |
| **ICC 2022** | **WHO 2017** | **WHO 2022** |
| 1. Bone marrow dysfunction caused by neoplastic mast cell infiltration, manifested by  ≥1 cytopenia:  ANC <1.0 × 10^9^/L,  Hb level <10 g/dL,  and/or PLT <100 × 10^9^/L | 1. Bone marrow dysfunction caused by neoplastic mast cell infiltration, manifested by  ≥1 cytopenia:  ANC <1.0 × 10^9^/L,  Hb level <10 g/dL,  and/or PLT <100 × 10^9^/L | 1. Cytopenia/s (one or more found):           ANC <1 × 10^9^/L           Hb <10 g/dL           PLT <1.0 × 10^9^/L |
| 2. Palpable hepatomegaly with impairment of liver function, ascites and/or portal hypertension | 2. Palpable hepatomegaly with impairment of liver function, ascites and/or portal hypertension | 2.  Hepatopathy: ascites and elevated liver enzymes^c^ ± hepatomegaly or cirrhotic liver ± portal hypertension |
| 3. Skeletal involvement, with large osteolytic lesions with or without pathological fractures (pathological  fractures caused by osteoporosis do not qualify as a C finding) | 3. Skeletal involvement, with large osteolytic lesions with or without pathological fractures (pathological  fractures caused by osteoporosis do not qualify as a C finding) | 3. Spleen: palpable splenomegaly with hypersplenism ± weight loss ± hypalbuminaemia |
| 4. Palpable splenomegaly with hypersplenism | 4. Palpable splenomegaly with hypersplenism | 4.  GI tract: malabsorption with hypoalbuminaemia ± weight loss |
| 5. Malabsorption with weight loss due to gastrointestinal mass cell infiltrates | 5. Malabsorption with weight loss due to gastrointestinal mass cell infiltrates | 5. Bone: large-sized osteolysis (≥20 mm) ± pathological fracture ± bone pain |

Updated since WHO 2017 (terminology/wording aligned between ICC 2022 and WHO 2022)

Updated since WHO 2017 (terminology/wording different between ICC 2022 and WHO 2022)

Minimal or no changes since WHO 2017

**Key**

AHN, associated haematological neoplasm; ANC, absolute neutrophil count; BM, bone marrow; CT, computed tomography; GI, gastrointestinal; HαT, hereditary alpha-tryptasaemia; Hb, haemoglobin; IHC, immunohistochemistry; LN, lymph node; MDS, myelodysplastic syndrome; MPN, myeloproliferative neoplasm; PB, peripheral blood; PLT, platelet count; SM, systemic mastocytosis; SSM, smoldering systemic mastocytosis; ULS, ultrasound; VAF, variant allele frequency.

^a^In the case of a known HαT, the basal serum tryptase level could be adjusted. Although the optimal way of adjustment still needs to be defined, one way is to divide the basal tryptase level by 1 plus the extra copy numbers of the alpha tryptase gene. For example, when the tryptase level is 300 and two extra copies of the alpha tryptase gene are found in a patient with HαT, the HαT-corrected tryptase level is 100 (300/3 = 100) and would thus not qualify as a B-finding.

^b^Signs of myeloproliferation and/or myelodysplasia must be discrete and stable (neither disappear nor progress) and must not reach diagnostic criteria of an MPN, MDS, or MPN/MDS in which case the diagnosis changes to SM-AHN. The presence of a myeloid AHN excludes B-findings and SSM by definition.

Supplementary Table 4. Rare genetic alterations in AML as defined by the ICC 2022 and WHO 2022 classifications

| **ICC 2022** | **WHO 2022** |
| --- | --- |
| **AML with other rare recurring translocations**   - AML with t(1;3)(p36.3;q21.3)/PRDM16::RPN1 - AML with t(3;5)(q25.3;q35.1)/NPM1::MLF1 - AML with t(8;16)(p11.2;p13.3)/KAT6A::CREBBP - AML (megakaryoblastic) with t(1;22)(p13.3;q13.1)/RBM15::MRTF1* - AML with t(5;11)(q35.2;p15.4/ NUP98::NSD1* - AML with t(11;12)(p15.4;p13.3)/NUP98::KMD5A* - AML with NUP98 and other partners* - AML with t(7;12)(q36.3;p13.2)/ETV6::MNX1* - AML with t(10;11)(p12.3;q14.2)/PICALM::MLLT10 - AML with t(16;21)(p11.2;q22.2)/FUS::ERG - AML with t(16;21)(q24.3;q22.1)/RUNX1::CBFA2T3 - AML with inv(16)(p13.3q24.3)/CBFA2T3::GLIS2* | **AML with other defined genetic alterations**   - AML with t(3;5)(q25.3;q35.1)/NPM1::MLF1 - AML with t(8;16)(p11.2;p13.3)/KAT6A::CREBBP - AML with t(7;12)(q36.3;p13.2)/ETV6::MNX1* - AML with t(16;21)(p11.2;q22.2)/FUS::ERG - AML with inv(16)(p13.3q24.3)/CBFA2T3::GLIS2* |

Alignment between ICC 2022 and WHO 2022 classification

Differences between ICC 2022 and WHO 2022 classification

**Key**

*Occurs predominantly in infants and children
